# Shenling Qige Granules inhibit esophageal squamous cell carcinoma progression in TE-1 xenograft and cell-based models by modulating the DCN–TGF-β/Smad signaling axis

**DOI:** 10.1371/journal.pone.0359405

**Published:** 2026-10-06

**Authors:** Zihui Liu, Yiting Gu, Huanfang Fan, Mao Huang, Dehui Li, Wen Guo, Yun He, Shuang Zhang, Zhihua Du

**Affiliations:** 1 Graduate School, Hebei University of Chinese Medicine, Shijiazhuang, Hebei, P.R. China; 2 Second Department of Oncology, The First Affiliated Hospital of Hebei University of Chinese Medicine, Shijiazhuang, Hebei, P.R. China; 3 Department of Pediatric Rehabilitation, The First Affiliated Hospital of Hebei University of Chinese Medicine, Shijiazhuang, Hebei, P.R. China; The University of Illinois at Chicago, UNITED STATES OF AMERICA

## Abstract

Shenling Qige Granules (SLQG), derived from the Qing Dynasty formula Qige San recorded in *Yi Xue Xin Wu*, has been used in clinical practice in China as an adjuvant traditional Chinese medicine preparation for esophageal squamous cell carcinoma (ESCC), but its molecular basis remains unclear. This study investigated the antitumor effects and mechanisms of SLQG using TE-1 xenograft mice and SLQG-containing serum-treated TE-1 cells. Transcriptome sequencing, network pharmacology, molecular docking, molecular dynamics simulation, and public-database analysis were integrated to prioritize candidate mechanisms. Histological, molecular, cellular, and siRNA-mediated decorin (*DCN*) knockdown assays were performed for validation. SLQG, especially at the medium dose, inhibited xenograft tumor growth and increased DCN expression. Transcriptomic analysis highlighted the transforming growth factor-β (TGF-β)/Smad pathway. In silico analyses suggested that several SLQG-derived compounds, particularly ursolic acid, may be associated with DCN-related regulation. Public-database analysis indicated lower *DCN* expression in esophageal carcinoma tumor samples than in normal samples and an association with pathological stage. In vivo and in vitro, SLQG increased DCN expression, reduced Smad2/3 phosphorylation, promoted pro-apoptotic signaling, and induced G_0_/G_1_ arrest; these effects were attenuated by *DCN* knockdown. These findings suggest that SLQG inhibited ESCC progression in TE-1 xenograft and cell-based models, potentially through modulation of the DCN-associated TGF-β/Smad signaling axis.

## 1. Introduction

Esophageal cancer (EC) is an aggressive malignancy originating from the esophageal epithelium. Esophageal squamous cell carcinoma (ESCC) is the predominant histological subtype in China, accounting for approximately 85.96% of EC cases [1]. According to GLOBOCAN 2022, EC remains a major cancer burden in China, which accounts for nearly half of global EC incidence and mortality [2]. Although surgery, radiotherapy, chemotherapy, immunotherapy and multimodal treatment have improved disease management, the prognosis of EC remains unsatisfactory, with a 5-year survival rate below 20% and frequent postoperative recurrence [3,4]. These limitations highlight the need to identify additional therapeutic strategies and biologically supported adjuvant interventions.

Shenling Qige Granules (SLQG) is a hospital preparation derived from Qige San, a classical traditional Chinese medicine (TCM) formula recorded in *Yi Xue Xin Wu* for “Yege”-related obstructive esophageal symptoms. The major manifestations of “Yege”, especially dysphagia and food obstruction, overlap with the obstructive symptoms commonly observed in ESCC. This traditional and clinical background supports the investigation of SLQG as an adjuvant TCM preparation in ESCC-related preclinical models. Previous studies and reviews on Qige San-derived or related TCM formulations have reported potential benefits in symptom relief, reduced treatment-related toxicity, improved quality of life and regulation of tumor-related biological processes in ESCC [5–8]. However, the pharmacological basis and molecular mechanism underlying the antitumor activity of SLQG remain insufficiently defined.

The same prescription was previously reported under the name Huazhuo Runzao Jiangqi Formula and has since been approved as the hospital preparation SLQG, with no change in formulation composition. Our group previously characterized this prescription using ultra-high-performance liquid chromatography-quadrupole-Orbitrap high-resolution mass spectrometry (UHPLC-Q-Orbitrap HRMS) and identified 46 constituents, thereby providing the phytochemical basis for the present study [9]. Building on this previous chemical characterization, the present study investigated the pharmacological basis of SLQG at the formula level, with particular emphasis on DCN-associated signaling regulation identified through transcriptomic and integrative analyses. To this end, we integrated newly generated transcriptome sequencing data, network pharmacology, molecular docking, molecular dynamics simulation, Gene Expression Profiling Interactive Analysis (GEPIA)-based public-database analysis, TE-1 xenograft experiments, SLQG-containing serum-treated TE-1 cell assays, and siRNA-mediated decorin (*DCN*) knockdown to investigate the potential involvement of the DCN-associated TGF-β/Smad signaling axis [10,11].

## 2. Materials and methods

### 2.1. Cells and animals

Human ESCC TE-1 cells were obtained from Procell Life Science & Technology Co., Ltd. (Wuhan, China; Cat. No. CL-0231). According to the supplier’s certificate, the cells were authenticated by short tandem repeat profiling and tested negative for mycoplasma contamination using a PCR-based assay before shipment. In addition, mycoplasma contamination was routinely monitored during cell culture, and no positive results were observed throughout the experimental period. Cells were cultured in RPMI 1640 medium supplemented with 10% fetal bovine serum (FBS) and 1% penicillin–streptomycin in a humidified incubator at 37 °C with 5% CO_2_.

Male BALB/c-nu nude mice (6 weeks old, 20 ± 2 g; *n* = 56) were used for xenograft experiments. Male Sprague-Dawley rats (200 ± 20 g; *n* = 20) were used for the preparation of SLQG-containing serum. All animals were purchased from Silaike Jingda Laboratory Animal Co., Ltd. (Hunan, China) and housed in the Experimental Animal Center of the First Affiliated Hospital of Hebei University of Chinese Medicine under a 12-h light/dark cycle with free access to food and water.

All animal procedures were reviewed and approved by the Institutional Animal Care and Use Committee of the First Affiliated Hospital of Hebei University of Chinese Medicine (approval No. IACUC-HPHCM-2024065; approval date: 20 July 2024). All experiments were performed in accordance with the approved protocol, relevant institutional guidelines and regulations, and the ARRIVE guidelines. This study did not involve the recruitment of human participants, collection of human tissues or specimens, or use of identifiable human participant data; therefore, written or verbal informed consent was not applicable. The public-database analyses used only publicly available, de-identified data. Death was not used as a planned experimental endpoint in either the nude mouse xenograft experiment or the Sprague-Dawley rat serum-preparation experiment.

Humane endpoints were predefined for all animals. Animals were to be humanely euthanized before the scheduled endpoint if any of the following criteria occurred: loss of more than 20% of initial body weight; food intake reduced to less than 50% of normal intake or anorexia lasting longer than 48 h; severe weakness or prolonged inability to stand; obvious infection; or signs consistent with organ failure. All remaining animals were euthanized at the scheduled completion of the predefined scientific objective. General health status, food intake, posture, mobility, body condition, and signs of distress were monitored daily. Body weight was recorded every other day in nude mice and during the serum-preparation period in rats. Tumor size in tumor-bearing nude mice was measured every other day as an indicator of tumor burden. Animals meeting humane endpoint criteria were to be euthanized immediately after confirmation by trained personnel under deep isoflurane anesthesia. No animals died before the scheduled experimental endpoint or before meeting criteria for euthanasia.

### 2.2. Drugs and reagents

Shenling Qige Granules (SLQG) consisted of Glehniae Radix [the dried root of *Glehnia littoralis* (J.G.Cooper) F.Schmidt ex Miq., 10 g], Fritillariae Thunbergii Bulbus [the dried bulb of *Fritillaria thunbergii* Miq., 10 g], Poria [the dried sclerotium of *Wolfiporia cocos* (F.A.Wolf) Ryvarden & Gilb., 10 g], Amomi Fructus [the dried ripe fruit of *Wurfbainia villosa* (Lour.) Škorničk. & A.D.Poulsen, synonym *Amomum villosum* Lour., 6 g], Curcumae Radix [the dried tuberous root of *Curcuma wenyujin* Y.H.Chen & C.Ling, 6 g], Salviae Miltiorrhizae Radix et Rhizoma [the dried root and rhizome of *Salvia miltiorrhiza* Bunge, 10 g], Scorpio [the dried body of *Buthus martensii* Karsch, 3 g], Pogostemonis Herba [the dried aerial part of *Pogostemon cablin* (Blanco) Benth., 10 g], Eupatorii Herba [the dried aerial part of *Eupatorium japonicum* Thunb., synonym *Eupatorium fortunei* Turcz., 10 g], Rabdosiae Rubescentis Herba [the dried aerial part of *Isodon rubescens* (Hemsl.) H.Hara, 30 g], and Nelumbinis Folium [the dried leaf of *Nelumbo nucifera* Gaertn., 3 g].

The botanical names of all plant-derived materials were verified using World Flora Online (WFO, http://www.worldfloraonline.org; accessed on 31 May 2026). Non-plant biological materials were reported using their full scientific names with authorities.

All components were supplied as granule formulations by Guangdong Yifang Pharmaceutical Co., Ltd. (Guangdong, China). All granules were manufactured under standardized quality-control procedures established by the manufacturer. Because commercially prepared granules rather than field-collected crude medicinal materials were used in this study, voucher specimens were not deposited. The batch numbers of the granule components were as follows: Glehniae Radix, 8062173; Fritillariae Thunbergii Bulbus, 8121873; Poria, 9051723; Amomi Fructus, 8091753; Curcumae Radix, 8122063; Salviae Miltiorrhizae Radix et Rhizoma, 9042003; Scorpio, 8110063; Pogostemonis Herba, 8066963; Eupatorii Herba, 8125563; Rabdosiae Rubescentis Herba, 9025253; and Nelumbinis Folium, 8090223.

The formulation investigated in the present study is currently named SLQG after approval as a hospital preparation. Prior to this name change, the same prescription, with unchanged formulation composition, was reported by our group as Huazhuo Runzao Jiangqi Formula. The UHPLC-Q-Orbitrap HRMS-based chemical characterization reported in our previous study therefore represents the chemical profile of SLQG and provides the phytochemical basis for the present DCN-centered mechanistic investigation. The 46 previously identified constituents are summarized in S1 Table in S1 File based on our previous chemical characterization study [9].

Cisplatin (DDP) and the transforming growth factor-β (TGF-β) receptor inhibitor SB431542 were purchased from MedChemExpress (MCE, USA). RPMI 1640 medium, phosphate-buffered saline (PBS), and penicillin-streptomycin were purchased from Servicebio (Wuhan, China). FBS and 0.25% trypsin–EDTA were purchased from Gibco (USA). Primary antibodies against GAPDH, β-actin, cleaved caspase-3, BAX, BCL2, Cyclin D1, and p21 were purchased from Proteintech (Wuhan, China; Cat. Nos. 10494-1-AP, 20536-1-AP, 25128-1-AP, 50599-2-Ig, 12789-1-AP, 60186-1-Ig, and 10355-1-AP, respectively). The anti-decorin antibody was purchased from Abcam (UK; Cat. No. ab277636). The anti-Smad2/3 antibody was purchased from Selleck (USA; Cat. No. F0363). Anti-phospho-Smad2/3 (Thr8) and anti-phospho-Smad2 (Ser465/467)/Smad3 (Ser423/425) antibodies were purchased from Immunoway (USA; Cat. No. YM8550) and ABclonal (Wuhan, China; Cat. No. AP0548), respectively. HRP-conjugated goat anti-mouse and goat anti-rabbit secondary antibodies were purchased from Proteintech (Wuhan, China; Cat. Nos. SA00001-1 and SA00001-2, respectively).

### 2.3. In vivo experiment and integrative analyses

#### 2.3.1. Model establishment, grouping and drug administration.

A total of 56 BALB/c-nu nude mice were acclimated for 1 week. Eight mice were randomly assigned to the Blank group without tumor inoculation, and the remaining 48 mice were used to establish TE-1 xenograft models. TE-1 cells in the logarithmic growth phase were harvested, washed with PBS, digested with trypsin, centrifuged, resuspended in PBS, and counted. Each mouse was subcutaneously inoculated in the right axillary region with 100 μL of cell suspension containing 1 × 10^6^ TE-1 cells [12]. Tumor growth and general condition were monitored daily. When tumors reached approximately 5 mm in diameter, mice were enrolled for treatment allocation [13].

The tumor-bearing mice were randomly divided into six groups (*n* = 8 per group): Model, SLQG-L, SLQG-M, SLQG-H, DDP, and SB431542. The mouse dose of SLQG was calculated from the clinical dose using the body surface area normalization method. SLQG-M was prepared at a crude drug concentration of 0.820 g/mL, whereas SLQG-L and SLQG-H were prepared at 0.410 and 1.640 g/mL, respectively. Mice in the SLQG groups received the corresponding SLQG solution by oral gavage at 0.2 mL/10 g body weight once daily. Mice in the Blank and Model groups received an equal volume of 0.9% saline by oral gavage. Mice in the DDP group received cisplatin (5 mg/kg, i.p.) once every 5 days for a total of three administrations, whereas mice in the SB431542 group received SB431542 (10 mg/kg, i.p.) once daily. All treatments were continued for 14 consecutive days. This experiment was designed as a short-term tumor-growth inhibition study rather than a survival study, and death was not used as a planned experimental endpoint. The xenograft experiment and subsequent transcriptomic analysis were performed using the same experimental cohort. After completion of treatment, tumor tissues were collected, and representative samples from the Model, SLQG-L, SLQG-M and SLQG-H groups were randomly selected for RNA sequencing.

#### 2.3.2. Sample collection.

On day 15 after treatment initiation, mice were deeply anesthetized with isoflurane for terminal blood collection and tissue harvesting. Adequate anesthesia was confirmed by the absence of response to toe pinch before blood collection. Blood was collected by retro-orbital puncture under deep anesthesia, allowed to clot at 4 °C for 2 h, and centrifuged at 3000 rpm for 15 min to obtain serum, which was stored at −80 °C. Mice were then euthanized under deep isoflurane anesthesia according to the approved animal protocol, and death was confirmed before tumor excision. Tumor tissues were rapidly excised and weighed. A portion of each tumor was fixed in 4% paraformaldehyde, and the remaining tissue was snap-frozen in liquid nitrogen for subsequent analyses. No animals were excluded from the final analysis, and no animals died before the scheduled endpoint or required unscheduled euthanasia.

#### 2.3.3. Transcriptome sequencing.

Tumor tissues from four mice in each of the Model, SLQG-L, SLQG-M and SLQG-H groups were randomly selected for transcriptome sequencing. Total RNA was isolated and purified using TRIzol reagent (Invitrogen, Carlsbad, CA, USA) according to the manufacturer’s instructions. RNA quantity and purity were assessed using a NanoDrop ND-1000 spectrophotometer (NanoDrop, Wilmington, DE, USA). RNA integrity was evaluated using a Bioanalyzer 2100 system (Agilent, CA, USA), and samples with an RNA integrity number (RIN) > 7.0, A260/A280 ratio > 1.8 and total RNA amount > 1 μg were used for library construction.

Poly(A) RNA was purified from total RNA using Dynabeads Oligo(dT)25 (Thermo Fisher Scientific, USA) and fragmented using the Magnesium RNA Fragmentation Module (NEB, USA). The fragmented RNA was reverse-transcribed to generate cDNA, followed by second-strand synthesis, end repair, A-tailing, indexed adapter ligation, and PCR amplification. The final cDNA libraries had an average insert size of approximately 300 ± 50 bp. Paired-end sequencing (2 × 150 bp, PE150) was performed on an Illumina NovaSeq 6000 platform according to the manufacturer’s protocol.

Raw reads were filtered using fastp, and clean reads were aligned to the human reference genome using HISAT2. Gene expression was quantified as fragments per kilobase of transcript per million mapped reads (FPKM). Differential expression analysis was performed using edgeR. The initial differential expression screening was conducted as an exploratory analysis using |log_2_FC| > 1 and nominal *P* < 0.05 as screening criteria. Gene Ontology (GO), Kyoto Encyclopedia of Genes and Genomes (KEGG) and gene set enrichment analysis (GSEA) were performed. GSEA thresholds were |NES| > 1, nominal *P* < 0.05 and false discovery rate (FDR) *q*-value < 0.25.

The raw transcriptome sequencing data generated in this study have been deposited in the NCBI Sequence Read Archive (SRA) under BioProject accession number PRJNA1477327.

#### 2.3.4. Network pharmacology.

Candidate compounds of SLQG were retrieved from the TCMSP (https://www.tcmsp-e.com/; accessed on 18 June 2025) and BATMAN-TCM (http://bionet.ncpsb.org/batman-tcm/; accessed on 18 June 2025) databases [14,15]. In TCMSP, compounds with oral bioavailability (OB) ≥ 30% and drug-likeness (DL) ≥ 0.18 were retained. In BATMAN-TCM, compounds with a score ≥ 20 and *P* < 0.05 were selected. Putative targets of the retained compounds were collected, deduplicated, and used to construct the “SLQG–candidate compound–target” network in Cytoscape 3.10.2. Candidate compounds were ranked according to degree centrality, and the top 10 compounds were selected for subsequent molecular docking with DCN. The network pharmacology analysis was independently performed in the present study to support the DCN-centered mechanistic investigation. These commonly used thresholds were selected to balance compound screening efficiency and the retention of potentially bioactive candidates in traditional Chinese medicine-related pharmacological studies.

#### 2.3.5. Molecular docking.

Molecular docking was performed to explore the potential computational associations between DCN, which was prioritized based on transcriptomic analysis of SLQG-treated tumor tissues, and the top 10 candidate compounds identified by network pharmacology. The docking analysis was considered exploratory and was used to generate hypotheses regarding possible compound–DCN interactions rather than to validate direct target engagement. The DCN structure was obtained from the AlphaFold database (https://alphafold.ebi.ac.uk/; accessed on 12 August 2025). Candidate compound structures were downloaded from PubChem (https://pubchem.ncbi.nlm.nih.gov/; accessed on 12 August 2025). Protein and ligand structures were prepared and converted into PDBQT format using AutoDockTools 1.5.6. Docking was performed using AutoDock Vina, and conformations were visualized using PyMOL 2.6.0. Docking energy was used to evaluate predicted binding affinity, with values <−7.0 kcal/mol considered relatively favorable.

#### 2.3.6. Molecular dynamics simulation.

Molecular dynamics simulations were performed using GROMACS 2020.3 for protein–ligand complexes with docking energies <−7.0 kcal/mol. A 100-ns production simulation was selected as a commonly used timescale for evaluating the conformational stability and dynamic behavior of protein–ligand complexes in computational pharmacology studies. The CHARMM36 force field and TIP3P water model were used. Each system was placed in a cubic box with a minimum distance of 1.0 nm from the box boundary and neutralized with Na⁺/Cl^−^ counterions. Energy minimization was performed using the steepest descent algorithm, followed by 100-ps equilibration under the NVT and NPT ensembles. Production simulations were then conducted for 100 ns at 300 K and 1 atm using the V-rescale thermostat and Parrinello-Rahman barostat. Van der Waals interactions were calculated using the Lennard–Jones potential with a 1.4-nm cutoff, and electrostatic interactions were computed using the particle mesh Ewald method with a Fourier spacing of 0.16 nm. Bond lengths were constrained using the LINCS algorithm. Trajectory analyses included root-mean-square deviation (RMSD), root-mean-square fluctuation (RMSF), hydrogen bonds (H-bonds), radius of gyration (Rg), and solvent-accessible surface area (SASA).

#### 2.3.7. GEPIA-based analysis of *DCN* expression in the ESCA cohort.

*DCN* expression and its association with pathological stage were analyzed using the GEPIA database (http://gepia.cancer-pku.cn/; accessed on 26 August 2025) [16]. Because GEPIA provides esophageal cancer data under the TCGA/GTEx esophageal carcinoma (ESCA) cohort rather than an ESCC-specific option, ESCA was used for this public-database analysis. Differential expression between tumor and normal tissues was analyzed using thresholds of |log_2_FC| > 1 and *P* < 0.01. Stage-specific *DCN* expression was evaluated across pathological stages I–IV. Gene expression levels were normalized as log_2_(TPM + 1), and statistical analysis was performed using the default GEPIA settings.

#### 2.3.8. Hematoxylin and eosin (H&E) staining.

H&E staining was used to evaluate tumor histopathology. Fresh tumor tissues were fixed in 4% paraformaldehyde for 36 h, dehydrated, paraffin-embedded, and sectioned at 4 μm. After deparaffinization, sections were stained using an H&E staining kit (Solarbio, China), mounted, and observed under a light microscope (Nikon, Japan) at 200× magnification.

#### 2.3.9. Body weight measurement.

Body weight was measured every other day from the day of model establishment.

#### 2.3.10. Tumor volume and growth curve.

The longest diameter (a) and shortest diameter (b) of each subcutaneous tumor were measured every other day using a vernier caliper. Tumor volume was calculated as V (mm^3^) = a × b^2^ / 2, and tumor growth curves were plotted accordingly.

#### 2.3.11. Tumor weight and tumor inhibition rate.

Tumors were weighed after excision. The tumor inhibition rate was calculated as follows: tumor inhibition rate (%) = [(mean tumor weight of the Model group − mean tumor weight of the treatment group) / mean tumor weight of the Model group] × 100%.

#### 2.3.12. Efficacy validation assays.

**2.3.12.1. TUNEL staining:** TUNEL staining was used to assess apoptosis in tumor tissues from the Model, SLQG-L, SLQG-M, SLQG-H and DDP groups. Paraffin sections were deparaffinized, rehydrated and treated with Proteinase K. The TUNEL reaction mixture (Roche, Switzerland) was applied for 1 h at 37 °C. Sections were counterstained with DAPI, mounted with anti-fade medium and imaged using a laser scanning confocal microscope (Nikon, Japan). The TUNEL-positive fluorescence ratio was quantified using ImageJ.

**2.3.12.2. Reverse transcription quantitative PCR (RT-qPCR):** Total RNA was extracted from frozen tumor tissues of the Model, SLQG-L, SLQG-M, SLQG-H and DDP groups using a total RNA extraction kit (Simgen, China). cDNA was synthesized using a reverse transcription kit (TIANGEN, China). qPCR was performed using FastUniversal qPCR PreMix (TIANGEN, China) on a real-time PCR system (Thermo Fisher Scientific, USA). The amplification program was 95 °C for 2 min, followed by 40 cycles of 95 °C for 5 s and 60 °C for 15 s. Relative gene expression was calculated using the 2^-ΔΔCt^ method. Primer sequences are listed in S2 Table in S1 File.

**2.3.12.3. Western blotting:** Frozen tumor tissues from the Model, SLQG-L, SLQG-M, SLQG-H and DDP groups were lysed in RIPA buffer (SevenBio, China) containing protease and phosphatase inhibitors (MCE, USA). Protein concentration was determined using a BCA kit (SevenBio, China). Equal amounts of protein were separated by electrophoresis and transferred onto membranes. After blocking, membranes were incubated overnight at 4 °C with primary antibodies against BAX (1:20,000), BCL2 (1:2,000), cleaved caspase-3 (1:500) and GAPDH (1:5,000), followed by HRP-conjugated secondary antibodies (1:5,000) for 1 h at room temperature. Bands were visualized using a chemiluminescence imaging system (Biolight, China), and band intensities were quantified using ImageJ. For each target protein, samples from all experimental groups were analyzed on the same gel and membrane to minimize inter-gel variability. Target proteins and corresponding loading controls (GAPDH or β-actin) were detected on the same membrane. Chemiluminescent images were acquired under non-saturated conditions, and the same acquisition criteria were applied for comparative analysis within each experiment. Relative protein expression levels were calculated by normalizing the intensity of each target band to the corresponding loading control. Total protein normalization methods, such as stain-free technology, were not applied in the present study.

#### 2.3.13. Mechanism validation assays.

**2.3.13.1. Immunohistochemistry (IHC):** IHC was performed using an IHC kit (Vector Laboratories, USA) to detect DCN, p-Smad2/3 and Cyclin D1 expression in tumor tissues from the Model, SLQG-L, SLQG-M, SLQG-H and SB431542 groups. Paraffin sections were deparaffinized, subjected to antigen retrieval in citrate buffer, and incubated with 3% H_2_O_2_ to block endogenous peroxidase activity. After serum blocking, sections were incubated overnight at 4 °C with primary antibodies against DCN (1:2,000), p-Smad2/3 (1:50) and Cyclin D1 (1:100), followed by secondary antibody incubation. Color development was performed using 3,3′-diaminobenzidine (DAB). Sections were counterstained with hematoxylin, mounted and quantified for mean optical density using ImageJ.

**2.3.13.2. Reverse transcription quantitative PCR (RT-qPCR):** Total RNA was extracted from frozen tumor tissues of the Model, SLQG-L, SLQG-M, SLQG-H and SB431542 groups. Reverse transcription and qPCR were performed as described in Section 2.3.12.2. Primer sequences are listed in S3 Table in S1 File.

**2.3.13.3. Western blotting:** Frozen tumor tissues from the Model, SLQG-L, SLQG-M, SLQG-H and SB431542 groups were processed as described in Section 2.3.12.3. Primary antibodies against DCN (1:1,000), Smad2/3 (1:1,000), p-Smad2/3 (1:2,000), p21 (1:2,000), Cyclin D1 (1:5,000), GAPDH (1:5,000) and β-actin (1:5,000) were used. Relative protein levels were normalized to the corresponding loading control (GAPDH or β-actin), which was detected on the same membrane as the target proteins. For each target protein, samples from all experimental groups were analyzed on the same gel and membrane to minimize inter-gel variability. Chemiluminescent images were acquired under non-saturated conditions using consistent imaging criteria.

### 2.4. In vitro experimental procedures

#### 2.4.1. Cell culture.

TE-1 cells were maintained as described in Section 2.1. Passage-3 cells at 80–90% confluence were used for in vitro experiments.

#### 2.4.2. Serum preparation.

Twenty male Sprague-Dawley rats were randomly divided into SLQG-containing serum and blank serum groups (*n* = 10 per group). The rat dose of SLQG was calculated from the clinical dose using the body surface area normalization method. SLQG was prepared at a crude drug concentration of 1.134 g/mL and administered by oral gavage at 1 mL/100 g body weight for 7 consecutive days. Rats in the blank serum group received an equal volume of 0.9% saline. Rats were fasted for 12 h before the final administration with free access to water.

The serum-preparation experiment was not designed as a survival study, and death was not used as a planned experimental endpoint. During the 7-day dosing period, rats were monitored daily for general appearance, food intake, posture, mobility, grooming, body condition, and signs of pain or distress. Humane endpoint criteria were predefined as follows: loss of more than 20% of initial body weight; food intake reduced to less than 50% of normal intake or anorexia lasting longer than 48 h; severe weakness or prolonged inability to stand; obvious infection; or signs consistent with organ failure. All remaining rats were euthanized at the scheduled completion of the predefined scientific objective. Animals meeting humane endpoint criteria were to be euthanized immediately after confirmation by trained personnel under deep isoflurane anesthesia. No rat reached humane endpoint criteria before scheduled terminal blood collection, and no rat died before the scheduled endpoint.

At 2 h after the final gavage, rats were deeply anesthetized with isoflurane for terminal blood collection. Adequate anesthesia was confirmed by the absence of response to toe pinch before abdominal aorta blood collection. Rats were euthanized by exsanguination under deep anesthesia according to the approved animal protocol, and death was confirmed before carcass disposal. Blood samples were allowed to clot at 4 °C for 2 h and centrifuged at 3,000 rpm for 15 min. Serum was collected, heat-inactivated at 56 °C for 30 min, filtered through a sterile 0.22-μm filter, aliquoted, and stored at −80 °C.

#### 2.4.3. Cell viability assay.

TE-1 cells were seeded into 96-well plates at 5 × 10^3^ cells/mL, with 200 μL per well. Cells were assigned to the following groups: Control, 5%, 10%, 15%, and 20% SLQG-containing serum, and DDP (2 μg/mL). After 24 h of treatment, CCK-8 reagent (10 μL; MCE, USA) was added to each well, and cells were incubated for 40 min at 37 °C. Absorbance at 450 nm was measured using a microplate reader (Tecan, Austria). Cell viability (%) was calculated as [(absorbance of treated wells − absorbance of blank wells) / (absorbance of control wells − absorbance of blank wells)] × 100%. Based on these results, low and high concentrations of SLQG-containing serum were selected for subsequent experiments.

#### 2.4.4. EdU staining for cell proliferation.

TE-1 cells were seeded into 24-well plates at 5 × 10⁴ cells per well and divided into four groups: control group (15% blank serum), low-dose SLQG-containing serum group (5% SLQG-containing serum + 10% blank serum), high-dose SLQG-containing serum group (15% SLQG-containing serum), and SB431542 group (15% blank serum + 20 μM SB431542). The final serum concentration was maintained consistently at 15% in all groups by supplementing SLQG-containing serum with blank serum when required, thereby minimizing potential effects caused by differences in total serum concentration. After 24 h of treatment, cells were incubated with EdU working solution from an EdU proliferation assay kit (MCE, USA) for 2 h, fixed, permeabilized, and subjected to Click reaction. Nuclei were stained with Hoechst 33342. Images were captured using a fluorescence microscope (Zeiss, Germany), and EdU-positive cells were quantified using ImageJ.

#### 2.4.5. Flow cytometric analysis of apoptosis.

TE-1 cells were seeded in 6-well plates at 5 × 10^5^ cells per well. The grouping and serum normalization strategy were the same as described in Section 2.4.4. After 24 h of treatment, cells were collected, washed with ice-cold PBS, and resuspended in binding buffer. Cells were stained with Annexin V-FITC (5 μL) and propidium iodide (PI; 10 μL) using an Annexin V-FITC/PI apoptosis kit (Lianke, China), incubated in the dark for 5 min, and analyzed using a flow cytometer (Beckman Coulter, USA). Data were processed using FlowJo 10.6.

#### 2.4.6. Flow cytometric analysis of cell cycle.

TE-1 cells were cultured in T25 flasks at 1 × 10^6^ cells per flask. The grouping and serum normalization strategy were the same as described in Section 2.4.4. After 24 h of treatment, cells were collected, fixed overnight in 75% ethanol at 4 °C, and stained with DNA staining solution from a cell cycle staining kit (Lianke, China) for 30 min in the dark. Cell cycle distribution was analyzed by flow cytometry (Beckman Coulter, USA), and data were processed using FlowJo 10.6.

#### 2.4.7. Western blotting.

TE-1 cells were seeded in 6-well plates at 5 × 10^5^ cells per well. The grouping and serum normalization strategy were the same as described in Section 2.4.4. After 24 h of treatment, cells were lysed in RIPA buffer containing protease and phosphatase inhibitors and processed as described in Section 2.3.12.3. Primary antibodies against BAX (1:20,000), DCN (1:1,000), Smad2/3 (1:1,000), BCL2 (1:2,000), p-Smad2/3 (1:2,000), p21 (1:2,000), Cyclin D1 (1:5,000) and β-actin (1:5,000) were used. Relative protein levels were normalized to β-actin, which was detected on the same membrane as the target proteins. For each target protein, samples from all experimental groups were analyzed on the same gel and membrane to minimize inter-gel variability. Chemiluminescent images were acquired under non-saturated conditions using consistent imaging criteria.

#### 2.4.8. siRNA transfection.

TE-1 cells were seeded into 6-well plates at 1 × 10^5^ cells per well and transfected at 50–60% confluence. siRNA was prepared at 20 μM in RNase-free water. For each well, 5 μL siRNA stock and 6 μL transfection reagent were separately diluted in 125 μL Opti-MEM (Gibco, USA), mixed, incubated for 10 min and added to the cells. Cells were collected at 24 and 48 h after transfection to evaluate DCN knockdown efficiency at the mRNA and protein levels. For mechanism validation, cells were divided into four groups: si-NC + 15% blank serum (si-NC-BS), si-NC + 15% SLQG-containing serum (si-NC-CS), si-DCN + 15% blank serum (si-DCN-BS), and si-DCN + 15% SLQG-containing serum (si-DCN-CS). Western blotting was performed to detect p-Smad2/3, BAX and Cyclin D1 expression. siRNA and primer sequences are listed in S4 Table in S1 File.

### 2.5. Statistical analysis

Statistical analyses and data visualization were performed using SPSS 26.0 (IBM, USA) and GraphPad Prism 10.4.2 (GraphPad Software, USA). Data are presented as mean ± SD. The assumptions of parametric tests, including data distribution and variance homogeneity, were considered before performing ANOVA analyses. When applicable, appropriate statistical approaches were selected based on experimental design and data characteristics. Comparisons among multiple groups were performed using one-way ANOVA followed by Tukey’s or Dunnett’s post hoc test, as appropriate. Pairwise comparisons were performed using two-tailed Student’s t-test. *P* < 0.05 was considered statistically significant. The number of biological replicates for each experiment is indicated in the corresponding figure legends. Sample sizes were selected based on the requirements of the experimental design, previous experience with similar experimental models, and ethical considerations for animal studies. The numerical data underlying the quantitative results are provided in S1 Data.

## 3. Results

### 3.1. Transcriptomic analysis of tumor tissues

The animal grouping and experimental timeline are shown in Fig 1A. Transcriptome sequencing was performed to characterize SLQG-induced molecular changes in tumor tissues. Using |log_2_FC| > 1 and nominal *P* < 0.05 as exploratory screening criteria, 761 candidate DEGs were identified in the SLQG-L group compared with the Model group, including 313 upregulated and 448 downregulated genes. The SLQG-M group showed 1,008 DEGs, with 327 upregulated and 681 downregulated genes, whereas the SLQG-H group showed 327 DEGs, with 133 upregulated and 194 downregulated genes (Fig 1B). PCA showed clear separation between the Model and SLQG-M groups (Fig 1C), and hierarchical clustering further indicated distinct gene-expression profiles between these two groups (Fig 1D). Among the candidate DEGs identified in the exploratory transcriptomic analysis, DCN showed significant upregulation in the SLQG-M group compared with the Model group (nominal *P* = 0.0191) (Fig 1E).

**Fig 1:**
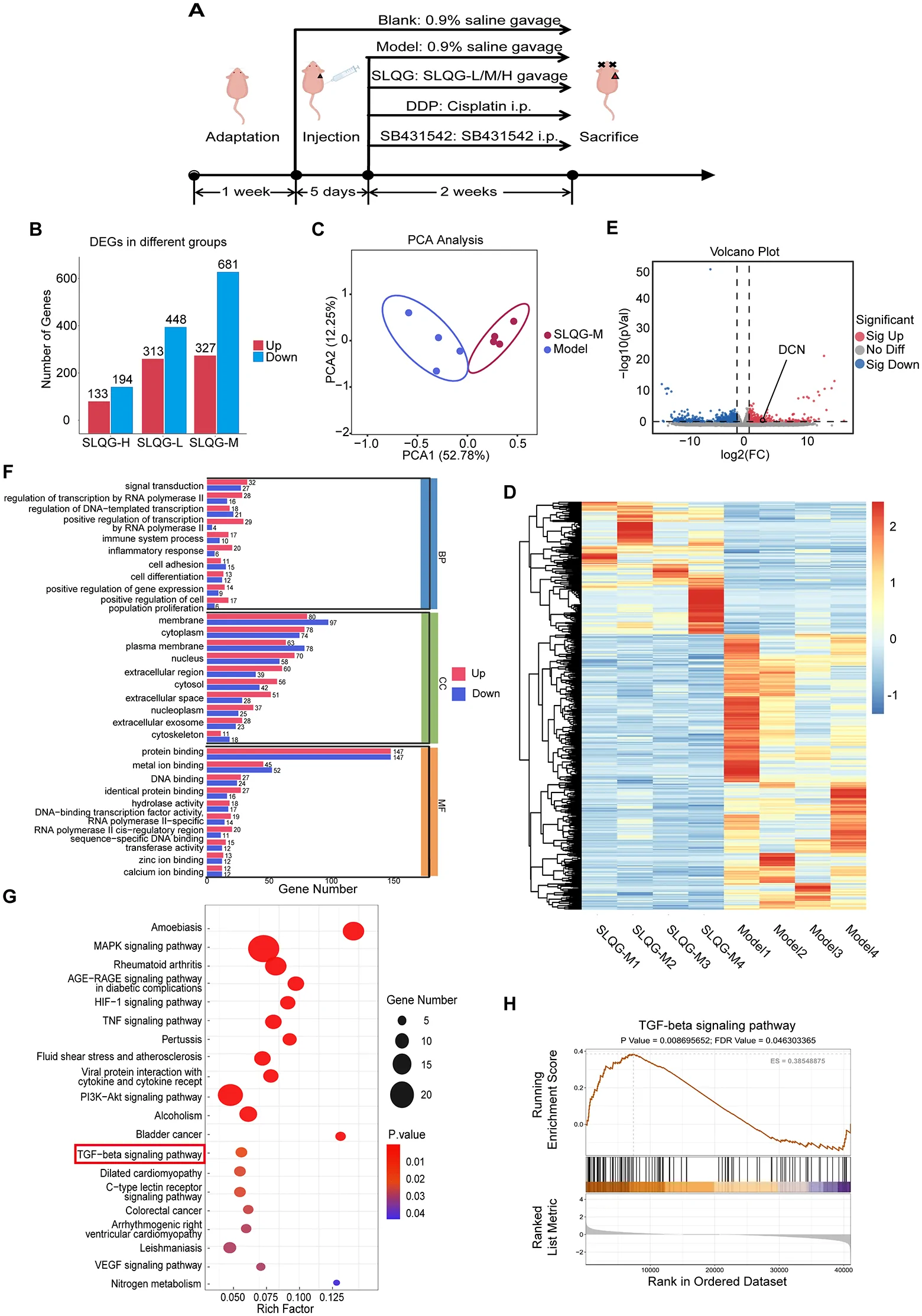
Transcriptomic profiling of SLQG-treated TE-1 xenograft tumors. (A) Schematic diagram of the animal grouping and experimental timeline. (B) Numbers of candidate DEGs identified using exploratory transcriptomic screening criteria in the SLQG-L, SLQG-M and SLQG-H groups compared with the Model group. (C) PCA of the Model and SLQG-M groups. (D) Heatmap of DEGs between the SLQG-M and Model groups. (E) Volcano plot of DEGs between the SLQG-M and Model groups. (F) GO enrichment analysis of DEGs. (G) KEGG enrichment analysis of DEGs. (H) GSEA of the TGF-β signaling pathway. RNA sequencing was performed using tumor tissues from the Model, SLQG-L, SLQG-M, and SLQG-H groups (*n* = 4 per group).

GO enrichment analysis identified 811 significantly enriched terms (*P* < 0.05), including 564 biological process terms, 55 cellular component terms and 192 molecular function terms. These terms were mainly associated with signal transduction, transcriptional regulation, membrane and cytoplasmic components, protein binding and metal ion binding (Fig 1F). KEGG enrichment analysis identified 55 significantly enriched pathways (*P* < 0.05), with cancer-related pathways, TGF-β signaling and PI3K/Akt signaling among the prominent pathways (Fig 1G). GSEA further showed significant enrichment of the TGF-β signaling pathway (*P* = 0.0087, FDR = 0.0463, ES = 0.3855) (Fig 1H). Together, these transcriptomic results identified *DCN* upregulation and TGF-β signaling enrichment as prominent molecular features of SLQG-treated tumor tissues.

### 3.2. Network pharmacology analysis

The candidate compounds of SLQG and their predicted targets were imported into Cytoscape 3.10.2 to construct the “SLQG–candidate compound–target” network. The network contained 123 candidate compounds, 511 predicted targets, 634 nodes and 2,756 edges (Fig 2A). Based on degree centrality, the top 10 candidate compounds were quercetin, β-sitosterol, luteolin, α-limonene, D-limonene, β-pinene, stigmasterol, elemicin, ursolic acid and tanshinone IIA (S5 Table in S1 File). α-Limonene and D-limonene were retained according to the original database annotations. These candidate compounds were considered potential contributors to the pharmacological effects of SLQG; however, they should not be interpreted as representing the sole active constituents responsible for the anti-ESCC activity of the whole formulation.

**Fig 2:**
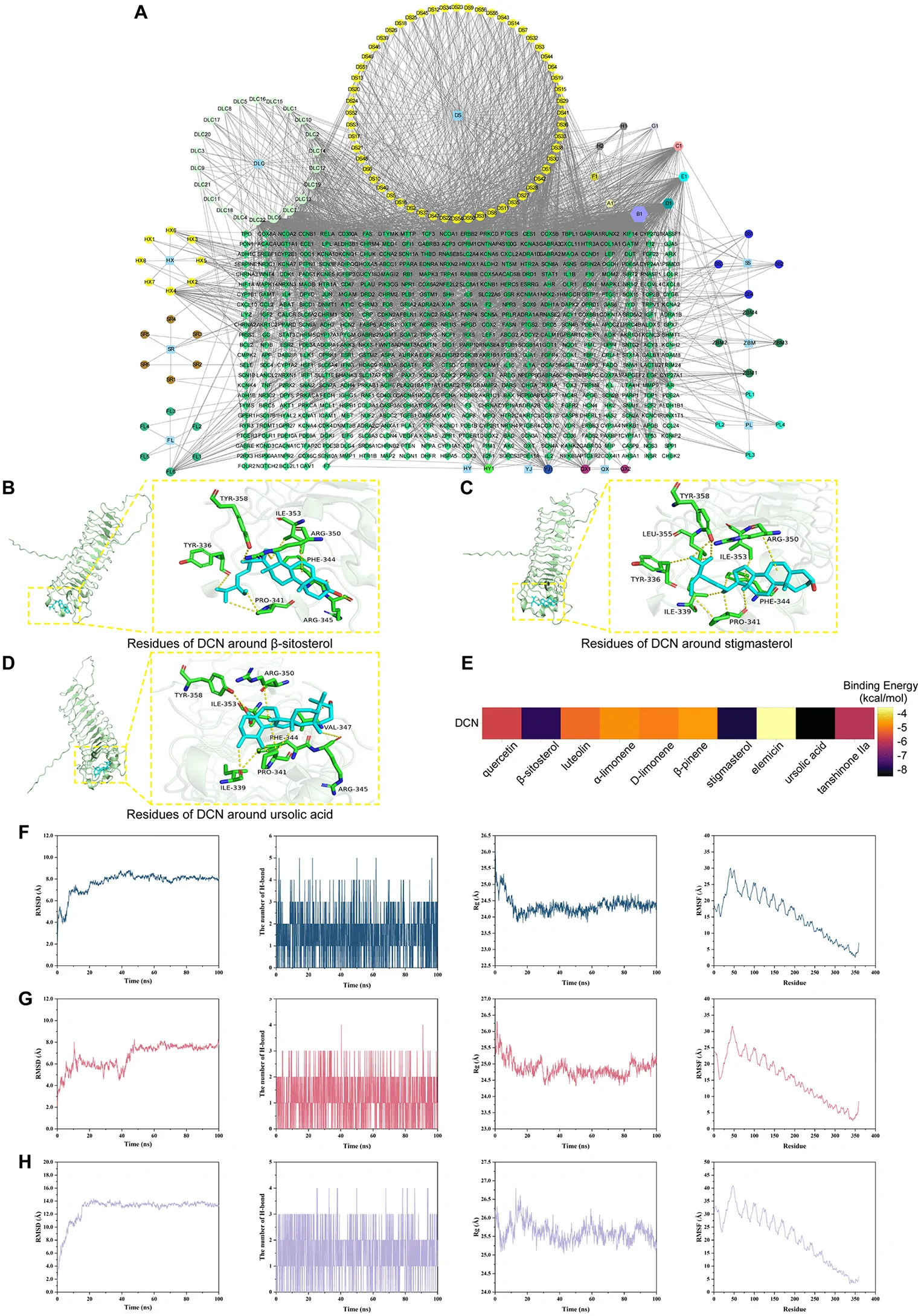
Network pharmacology, molecular docking, and molecular dynamics simulation analyses. (A) “SLQG–candidate compound–target” network constructed using Cytoscape 3.10.2. (B–D) Representative molecular docking conformations and interaction modes of β-sitosterol, stigmasterol, and ursolic acid with DCN. (E) Heatmap of binding energies between DCN and the top 10 candidate compounds ranked by degree centrality. (F–H) Molecular dynamics simulation analyses of the β-sitosterol–DCN, stigmasterol–DCN, and ursolic acid–DCN complexes, including RMSD, number of H-bonds, Rg, and RMSF.

### 3.3. Molecular docking analysis

Molecular docking was performed to explore the potential interaction patterns between candidate compounds and DCN. Docking energies ranged from −3.51 to −8.43 kcal/mol. Among the tested compounds, β-sitosterol, stigmasterol and ursolic acid showed docking energies below −7.0 kcal/mol, suggesting potentially favorable computational associations with DCN. Representative docking conformations and interaction modes are shown in Fig 2B–2D and S1 Fig in S1 File. The docking energy heatmap is shown in Fig 2E, and the corresponding docking energy values are summarized in S6 Table in S1 File.

### 3.4. Molecular dynamics simulation

Because β-sitosterol, stigmasterol and ursolic acid showed relatively favorable predicted docking affinities with DCN, 100-ns molecular dynamics simulations were performed to evaluate the stability of the corresponding protein–ligand complexes. The β-sitosterol–DCN and stigmasterol–DCN complexes reached relative equilibrium at approximately 40 ns, with mean RMSD values of 7.68 and 6.70 Å, respectively. The ursolic acid–DCN complex reached relative equilibrium at approximately 20 ns, with a mean RMSD value of 12.84 Å. The RMSF values ranged from 2.51 to 30.05 Å, 2.47 to 31.60 Å, and 3.15 to 40.98 Å for the β-sitosterol–DCN, stigmasterol–DCN, and ursolic acid–DCN complexes, respectively, with corresponding mean values of 15.82, 15.47, and 21.37 Å. The Rg values fluctuated within 23.82–26.02 Å, 24.32–26.31 Å, and 25.01–26.80 Å, with mean values of 24.34, 24.86, and 25.62 Å, respectively. The number of H-bonds ranged from 0 to 4, with average values of 1.54, 1.17, and 1.53, respectively. The SASA values fluctuated within 154.90–208.18, 148.22–184.76, and 146.93–184.35 nm^2^, with mean values of 167.59, 157.82, and 162.05 nm^2^, respectively. These findings suggested that the predicted compound–DCN complexes exhibited relatively stable conformational behavior during the simulation period in silico (Fig 2F–2H and S2 Fig in S1 File).

### 3.5. GEPIA analysis of *DCN* expression in the ESCA cohort

Because GEPIA does not provide an ESCC-specific query option, the TCGA/GTEx ESCA cohort was used as an exploratory public-database reference. *DCN* expression was analyzed in 182 ESCA tumor samples and 286 normal tissue samples. *DCN* mRNA expression was significantly lower in ESCA tumors than in normal tissues (*P* < 0.05) (Fig 3A). *DCN* expression also differed across pathological stages I–IV (*F* = 4.48, *P* = 0.00467), suggesting an association with tumor stage in this cohort (Fig 3B). These results provide supportive public-database evidence for *DCN* dysregulation in the TCGA/GTEx ESCA cohort.

**Fig 3:**
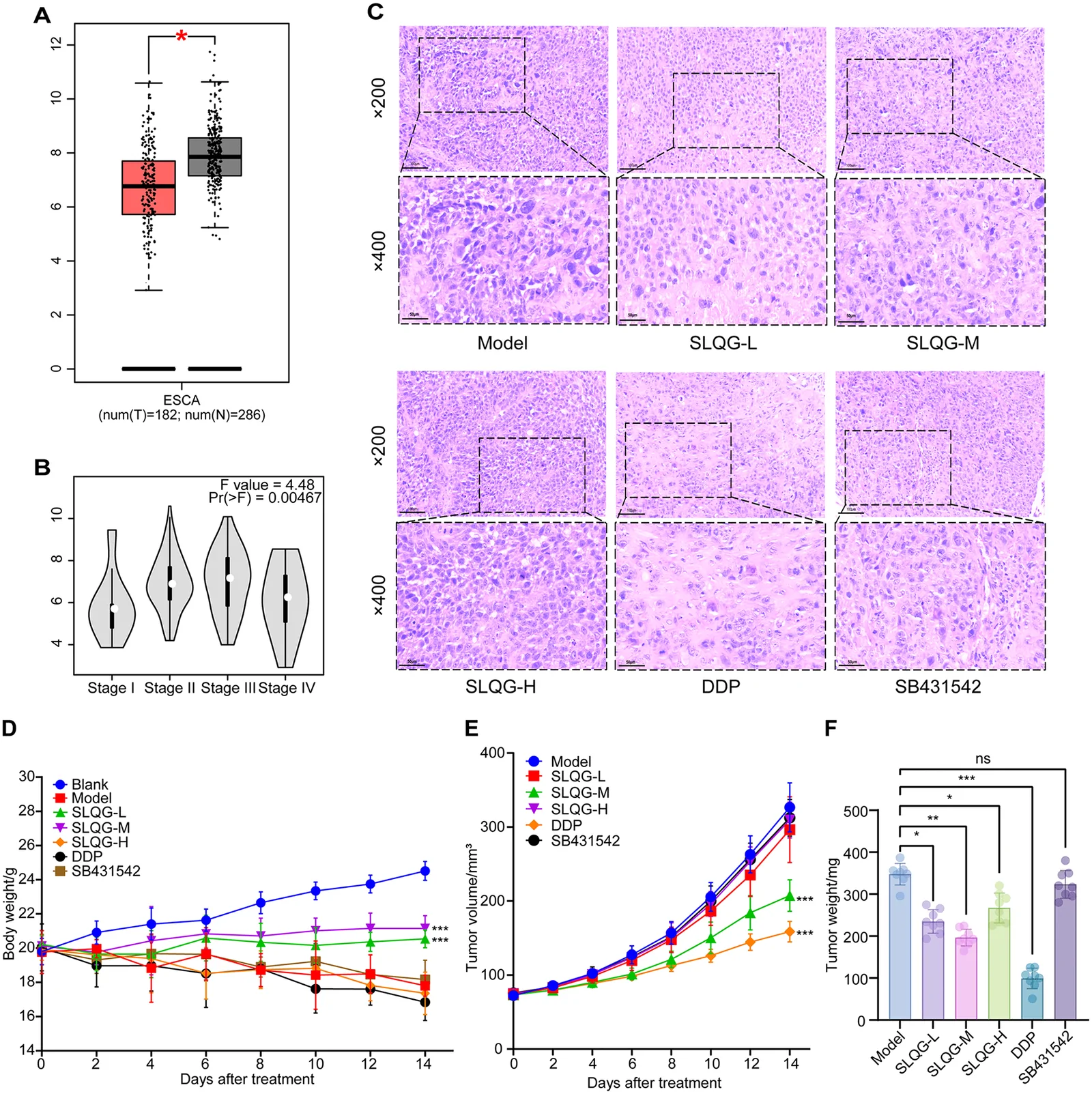
GEPIA-based analysis of *DCN* expression in the ESCA cohort and in vivo validation of the antitumor effects of SLQG. (A) *DCN* mRNA expression in tumor and normal tissues from the GEPIA ESCA cohort integrating TCGA and GTEx datasets. (B) Stage-specific *DCN* expression in the GEPIA ESCA cohort. (C) Hematoxylin and eosin (H&E) staining of tumor tissues in each group (scale bars: 100 μm and 50 μm for 200× and 400× magnification, respectively) (*n* = 3). (D) Body weight changes in each group. (E) Tumor growth curves. (F) Tumor weights and tumor inhibition rates in each group (*n* = 8). Data are presented as mean ± SD. Statistical significance was determined using one-way ANOVA followed by Dunnett’s multiple comparisons test. *P < 0.05, **P < 0.01, ***P < 0.001 versus the Model group.

### 3.6. Morphological changes in tumor tissues

Hematoxylin and eosin staining showed that tumors in the Model group exhibited disordered cellular arrangement, enlarged hyperchromatic nuclei, marked atypia and abundant mitotic figures, without obvious necrosis. In the DDP and SLQG-M groups, tumor tissues showed nuclear pyknosis and focal necrosis. Other treatment groups also showed varying degrees of structural disruption and necrosis, with fewer mitotic figures than the Model group (Fig 3C).

### 3.7. Body weight, tumor volume, and tumor weight

Baseline body weight did not differ significantly among groups before treatment (*P* > 0.05). During treatment, body weight increased gradually in the Blank group, remained relatively stable in the SLQG-L and SLQG-M groups, and decreased in the Model, SLQG-H, SB431542 and DDP groups. After treatment, body weight was significantly higher in the SLQG-L and SLQG-M groups than in the Model group (*P* < 0.001) (Fig 3D).

Baseline tumor volume did not differ significantly among tumor-bearing groups (*P* > 0.05). After treatment, tumor volume was significantly reduced in the SLQG-M and DDP groups compared with the Model group (*P* < 0.001), whereas the SLQG-L, SLQG-H and SB431542 groups showed no significant difference (Fig 3E). Tumor weight showed a similar pattern, with significant reductions in the SLQG-M and DDP groups (*P* < 0.01 and *P* < 0.001, respectively). The tumor inhibition rates were 71.50% in the DDP group, 43.43% in the SLQG-M group, 32.38% in the SLQG-L group, 23.13% in the SLQG-H group and 6.93% in the SB431542 group. Among the SLQG-treated groups, the medium dose showed the most pronounced antitumor effect (Fig 3F and Table 1).

**Table 1:** Effects of SLQG on tumor weight and tumor inhibition rate in tumor-bearing nude mice.

Group	Tumor weight (mg)	Tumor inhibition rate (%)
Model	347.51 ± 25.66	–
SLQG-L	234.99 ± 27.93^*^	32.38
SLQG-M	196.59 ± 20.07^**^	43.43
SLQG-H	267.15 ± 35.57^*^	23.13
DDP	99.04 ± 24.36^***^	71.50
SB431542	323.44 ± 33.17	6.93

Data are presented as mean ± SD (*n* = 8 per group). Statistical significance was determined using one-way ANOVA followed by Dunnett’s multiple comparisons test. *P < 0.05, **P < 0.01, ***P < 0.001 versus the Model group.

### 3.8. Efficacy validation

#### 3.8.1. Effects of SLQG on tumor cell apoptosis in nude mice.

TUNEL staining showed higher TUNEL-positive fluorescence ratios in the SLQG-L and SLQG-M groups than in the Model group (*P* < 0.05 and *P* < 0.01, respectively), whereas no significant difference was observed in the SLQG-H group. The DDP group showed the highest TUNEL-positive fluorescence ratio among the treatment groups (*P* < 0.001) (Fig 4A, 4B).

**Fig 4:**
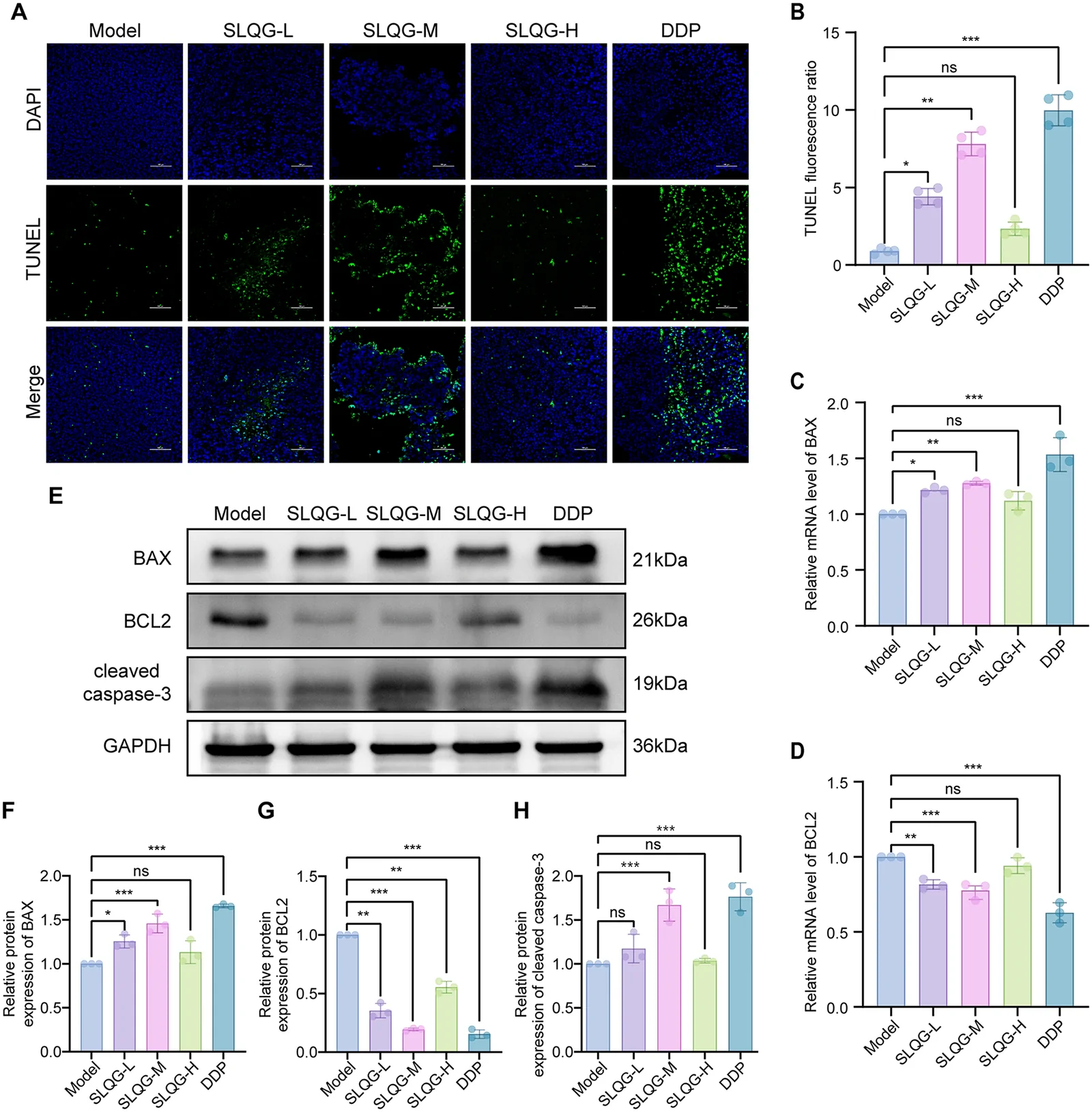
Effects of SLQG on apoptosis in tumor tissues of nude mice. (A) TUNEL staining of tumor tissues in the Model, SLQG-L, SLQG-M, SLQG-H, and DDP groups (scale bar: 100 μm, 200× magnification) (*n* = 4). (B) Quantitative analysis of the TUNEL-positive fluorescence ratio in tumor tissues (*n* = 4). (C, D) Relative mRNA expression levels of BAX and BCL2 in tumor tissues of nude mice (*n* = 3). (E) Representative Western blot images of BAX, BCL2, and cleaved caspase-3 protein expression. (F–H) Relative protein expression levels of BAX, BCL2, and cleaved caspase-3 in tumor tissues of nude mice (*n* = 3). Data are presented as mean ± SD. Statistical significance was determined using one-way ANOVA followed by Dunnett’s multiple comparisons test. *P < 0.05, **P < 0.01, ***P < 0.001 versus the Model group. The original uncropped and minimally adjusted Western blot images are provided in S1 Raw images.

#### 3.8.2. Effects of SLQG on *BAX* and *BCL2* mRNA expression in tumor tissues.

Compared with the Model group, *BAX* mRNA expression was significantly increased in the SLQG-L, SLQG-M and DDP groups (*P* < 0.05, *P* < 0.01 and *P* < 0.001, respectively), whereas *BCL2* mRNA expression was significantly decreased in these groups (*P* < 0.01, *P* < 0.01 and *P* < 0.001, respectively). No significant changes in *BAX* or *BCL2* mRNA expression were observed in the SLQG-H group (Fig 4C, 4D).

#### 3.8.3. Effects of SLQG on BAX, BCL2, and cleaved caspase-3 protein expression.

Compared with the Model group, BAX and cleaved caspase-3 protein expression was significantly increased in the SLQG-M and DDP groups (*P* < 0.001), whereas BCL2 protein expression was significantly decreased (*P* < 0.001). In the SLQG-L group, BAX protein expression was increased (*P* < 0.05), and BCL2 protein expression was decreased (*P* < 0.01). In the SLQG-H group, only BCL2 protein expression was significantly reduced (*P* < 0.01) (Fig 4E–4H).

### 3.9. Mechanism validation

#### 3.9.1. Effects of SLQG on DCN, p-Smad2/3, and Cyclin D1 expression in tumor tissues.

IHC staining showed higher DCN expression in the SLQG-L and SLQG-M groups than in the Model group (*P* < 0.05 and *P* < 0.001, respectively). In contrast, p-Smad2/3 expression was decreased in the SLQG-L, SLQG-M and SB431542 groups (*P* < 0.05, *P* < 0.001 and *P* < 0.001, respectively). Cyclin D1 expression was also decreased in these groups (*P* < 0.05, *P* < 0.001 and *P* < 0.001, respectively). Among the SLQG-treated groups, SLQG-M showed the most pronounced regulatory effect on these markers (Fig 5A–5D).

**Fig 5:**
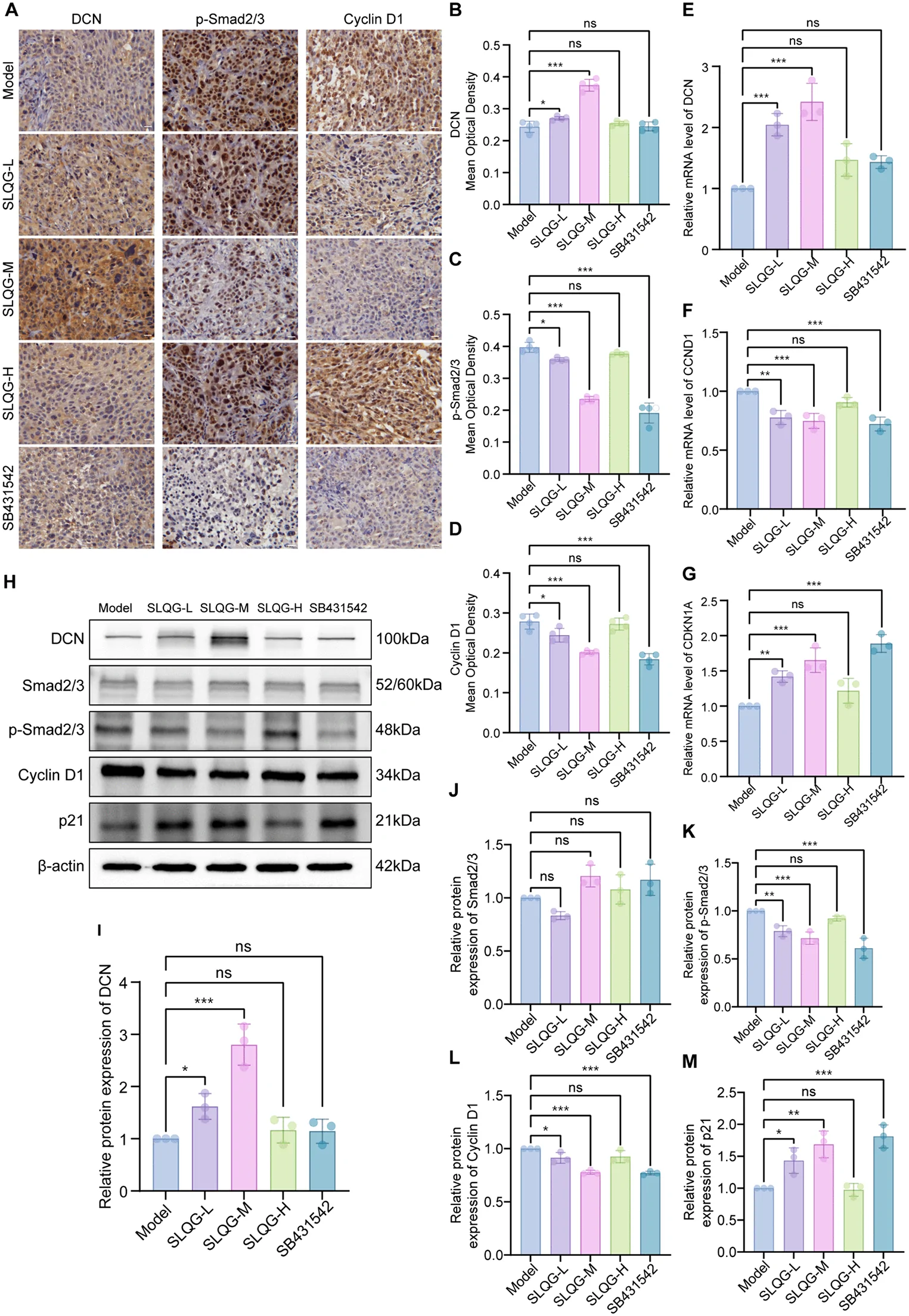
Mechanism validation of SLQG in tumor tissues. (A) Representative immunohistochemical (IHC) staining of DCN, p-Smad2/3, and Cyclin D1 in the Model, SLQG-L, SLQG-M, SLQG-H, and SB431542 groups (scale bar: 20 μm, 400× magnification) (*n* = 4). (B–D) Quantitative analysis of mean optical density (MOD) for DCN, p-Smad2/3, and Cyclin D1 in IHC staining (*n* = 4). (E–G) Relative mRNA expression levels of *DCN*, *CCND1*, and *CDKN1A* in tumor tissues (*n* = 3). (H) Representative Western blot images of DCN, Smad2/3, p-Smad2/3, Cyclin D1, and p21. (I–M) Relative protein expression levels of DCN, Smad2/3, p-Smad2/3, Cyclin D1, and p21 in tumor tissues (*n* = 3). Data are presented as mean ± SD. Statistical significance was determined using one-way ANOVA followed by Dunnett’s multiple comparisons test. *P < 0.05, **P < 0.01, ***P < 0.001 versus the Model group. The original uncropped and minimally adjusted Western blot images are provided in S1 Raw images.

#### 3.9.2. Effects of SLQG on *DCN*, *CCND1*, and *CDKN1A* mRNA expression in tumor tissues.

Compared with the Model group, *DCN* mRNA expression was significantly increased in the SLQG-L and SLQG-M groups (*P* < 0.001). *CCND1* mRNA expression was decreased in the SLQG-L, SLQG-M and SB431542 groups (*P* < 0.01, *P* < 0.001 and *P* < 0.001, respectively), whereas *CDKN1A* mRNA expression was increased in these groups (*P* < 0.01, *P* < 0.001 and *P* < 0.001, respectively) (Fig 5E–5G).

#### 3.9.3. Effects of SLQG on DCN, p-Smad2/3, Smad2/3, Cyclin D1, and p21 protein expression.

Compared with the Model group, DCN protein expression was increased in the SLQG-L and SLQG-M groups (*P* < 0.05 and *P* < 0.001, respectively). p-Smad2/3 protein expression was decreased in the SLQG-L, SLQG-M and SB431542 groups (*P* < 0.01, *P* < 0.001 and *P* < 0.001, respectively), whereas total Smad2/3 expression showed no significant difference among groups (*P* > 0.05). Cyclin D1 protein expression was also decreased in the SLQG-L, SLQG-M and SB431542 groups (*P* < 0.05, *P* < 0.001 and *P* < 0.001, respectively), while p21 expression was increased in these groups (*P* < 0.05, *P* < 0.01 and *P* < 0.001, respectively). Among the SLQG-treated groups, SLQG-M showed the most pronounced effects on DCN, p-Smad2/3, Cyclin D1 and p21 protein expression (Fig 5H–5M).

### 3.10. Effects of SLQG-containing serum on TE-1 cell viability

Compared with the control group, all SLQG-containing serum groups and the DDP group showed significantly reduced TE-1 cell viability. The DDP group showed the lowest viability (70.91 ± 6.46%, *P* < 0.001). Among the SLQG-containing serum groups, cell viability decreased from 5% to 15%, with viability rates of 88.92 ± 6.63% (*P* < 0.05), 86.11 ± 6.62% (*P* < 0.01) and 79.06 ± 8.67% (*P* < 0.001), respectively. The 20% SLQG-containing serum group showed a viability rate of 85.70 ± 3.98% (*P* < 0.01), indicating that increasing the concentration beyond 15% did not further enhance growth inhibition. Therefore, 5% and 15% SLQG-containing serum were selected as the low- and high-concentration conditions for subsequent experiments (Fig 6A).

**Fig 6:**
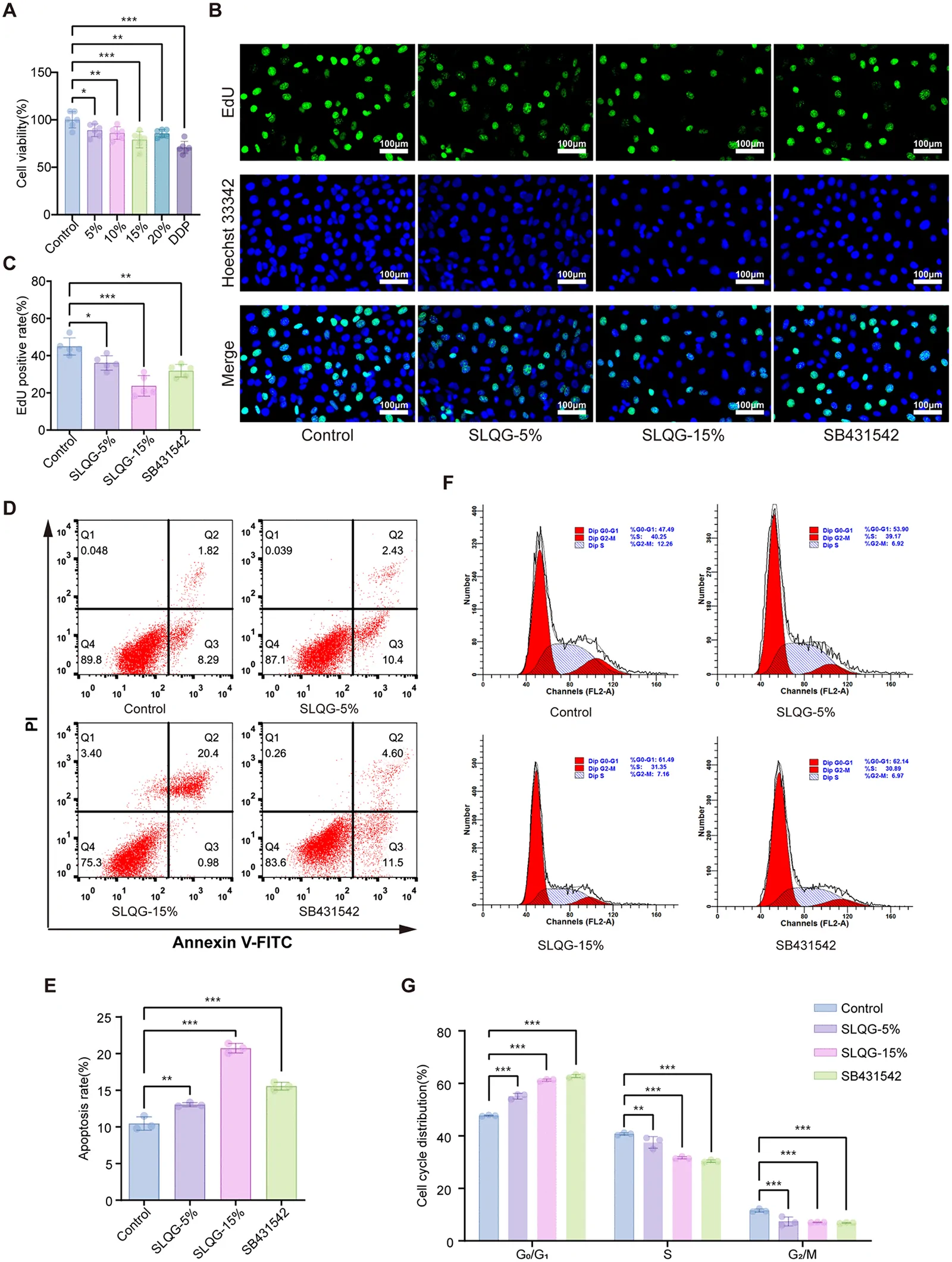
Effects of SLQG-containing serum on TE-1 cell function. (A) TE-1 cell viability detected by CCK-8 assay after treatment with SLQG-containing serum (*n* = 6). (B) Representative EdU staining images of TE-1 cells after treatment with SLQG-containing serum (scale bar: 100 μm, 200× magnification) (*n* = 5). (C) Quantitative analysis of TE-1 cell proliferation detected by EdU assay (*n* = 5). (D) Representative flow cytometry plots of apoptosis in TE-1 cells (*n* = 3). (E) Quantitative analysis of apoptosis in TE-1 cells (*n* = 3). (F) Representative flow cytometry plots of cell cycle distribution in TE-1 cells (*n* = 3). (G) Quantitative analysis of cell cycle distribution in TE-1 cells (*n* = 3). Data are presented as mean ± SD. Statistical significance was determined using one-way ANOVA followed by Dunnett’s multiple comparisons test. *P < 0.05, **P < 0.01, ***P < 0.001 versus the control group.

### 3.11. Effects of SLQG-containing serum on TE-1 cell proliferation

Compared with the control group, cell proliferation was significantly reduced in the SLQG-5%, SLQG-15%, and SB431542 groups (*P* < 0.05, *P* < 0.001, and *P* < 0.01, respectively). The inhibitory effect was more pronounced in the SLQG-15% group than in the SLQG-5% group (Fig 6B, 6C).

### 3.12. Effects of SLQG-containing serum on apoptosis of TE-1 cells

Compared with the control group, the apoptosis rate was significantly increased in the SLQG-5%, SLQG-15%, and SB431542 groups (*P* < 0.01, *P* < 0.001, and *P* < 0.01, respectively). The increase in apoptosis was more pronounced in the SLQG-15% group than in the SLQG-5% group (Fig 6D, 6E).

### 3.13. Effects of SLQG-containing serum on the cell cycle of TE-1 cells

Compared with the control group, the proportion of cells in the G_0_/G_1_ phase was significantly increased in the SLQG-5%, SLQG-15%, and SB431542 groups (*P* < 0.001). In contrast, the proportion of cells in S phase was significantly decreased in these groups (*P* < 0.01, *P* < 0.001, and *P* < 0.001, respectively), as was the proportion of cells in G_2_/M phase (*P* < 0.001). The effect on cell cycle distribution was more pronounced in the SLQG-15% group than in the SLQG-5% group (Fig 6F, 6G).

### 3.14. Effects of SLQG-containing serum on protein expression in TE-1 cells

Compared with the control group, BAX protein expression was increased in the SLQG-15% and SB431542 groups (*P* < 0.001 and *P* < 0.01, respectively), whereas BCL2 protein expression was decreased in these groups (*P* < 0.001 and *P* < 0.01, respectively). DCN protein expression was increased in the SLQG-5% and SLQG-15% groups (*P* < 0.05 and *P* < 0.001, respectively). p-Smad2/3 protein expression was decreased in the SLQG-5%, SLQG-15% and SB431542 groups (*P* < 0.05, *P* < 0.001 and *P* < 0.001, respectively), whereas total Smad2/3 expression did not differ significantly among groups (*P* > 0.05). Cyclin D1 protein expression was decreased in the SLQG-15% and SB431542 groups (*P* < 0.001 for both), while p21 protein expression was increased in these groups (*P* < 0.01 and *P* < 0.001, respectively) (Fig 7A–7H).

**Fig 7:**
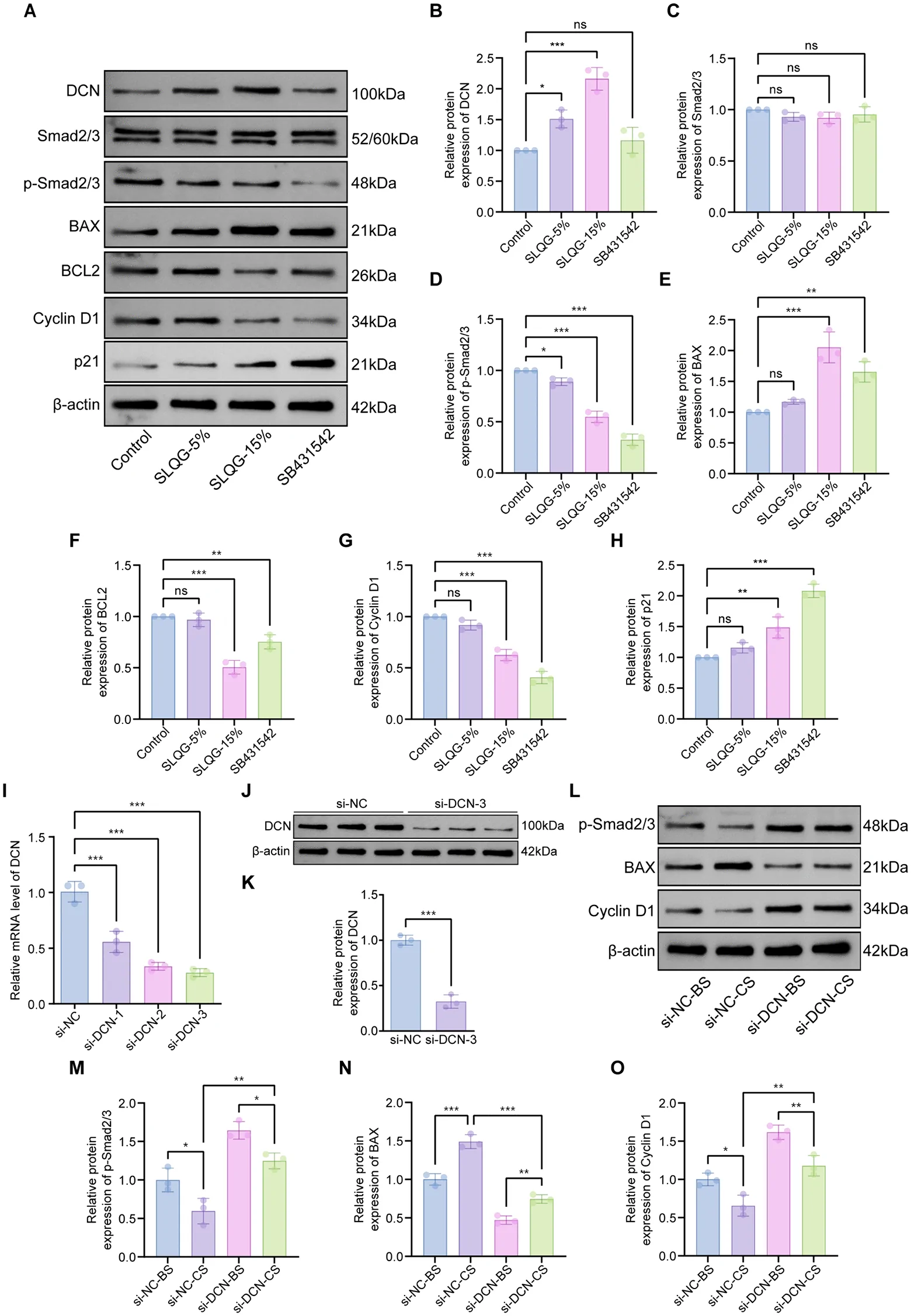
Effects of SLQG-containing serum and *DCN* knockdown on key protein expression in TE-1 cells. (A) Representative Western blot images of DCN, p-Smad2/3, Smad2/3, BAX, BCL2, Cyclin D1, and p21 protein expression in TE-1 cells (*n* = 3). (B–H) Relative protein expression levels of DCN, Smad2/3, p-Smad2/3, BAX, BCL2, Cyclin D1, and p21 in TE-1 cells after treatment with SLQG-containing serum (*n* = 3). (I) Validation of *DCN* knockdown efficiency in TE-1 cells by RT-qPCR (*n* = 3). (J, K) Validation of *DCN* knockdown efficiency at the protein level in TE-1 cells (*n* = 3). (L) Representative Western blot images of p-Smad2/3, BAX, and Cyclin D1 expression after *DCN* knockdown and SLQG-containing serum treatment (*n* = 3). (M–O) Relative protein expression levels of p-Smad2/3, BAX, and Cyclin D1 in the si-NC-BS, si-NC-CS, si-DCN-BS, and si-DCN-CS groups (*n* = 3). SLQG-containing serum decreased p-Smad2/3 and Cyclin D1 expression and increased BAX expression, whereas *DCN* knockdown partially reversed these effects, indicating that DCN contributes to SLQG-mediated regulation of TGF-β/Smad signaling, apoptosis-associated signaling, and cell cycle control. Data are presented as mean ± SD. Statistical significance was determined using one-way ANOVA followed by Tukey’s multiple comparisons test. *P < 0.05, **P < 0.01, ***P < 0.001, as indicated by the brackets. The original uncropped and minimally adjusted Western blot images are provided in S1 Raw images.

### 3.15. Effects of *DCN* knockdown on key molecules in vitro

*DCN* knockdown markedly reduced DCN expression at both the mRNA and protein levels, confirming the efficiency of siRNA-mediated silencing (Fig 7I–7K). Compared with the si-NC-BS group, the si-NC-CS group showed decreased p-Smad2/3 expression (*P* < 0.05), increased BAX expression (*P* < 0.001) and decreased Cyclin D1 expression (*P* < 0.05). After *DCN* knockdown, p-Smad2/3 expression was partially restored in the si-DCN-CS group compared with the si-NC-CS group (*P* < 0.01), whereas the increase in BAX expression and the decrease in Cyclin D1 expression were attenuated (*P* < 0.001 and *P* < 0.01, respectively) (Fig 7L–7O). These results indicate that *DCN* knockdown attenuated the effects of SLQG-containing serum on p-Smad2/3, BAX and Cyclin D1 expression in TE-1 cells.

## 4. Discussion

This study investigated the anti-ESCC activity and molecular mechanism of SLQG, a hospital preparation derived from Qige San, by integrating a previously established UHPLC-Q-Orbitrap HRMS-based chemical profile with transcriptomic analysis, network pharmacology, molecular docking, molecular dynamics simulations, public-database analysis, and in vivo and in vitro validation. The results suggest that SLQG inhibited ESCC progression in TE-1 xenograft and cell-based models. The in vivo xenograft findings further complemented the cellular observations by demonstrating that SLQG-associated molecular and phenotypic changes were accompanied by suppression of tumor growth within an in vivo biological environment. Mechanistically, SLQG increased DCN expression, reduced Smad2/3 phosphorylation, promoted apoptotic signaling, and induced G_0_/G_1_ cell cycle arrest. *DCN* knockdown partially attenuated the effects of SLQG-containing serum on p-Smad2/3, BAX, and Cyclin D1 expression, providing functional evidence that DCN contributes to the regulation of these molecular events. However, whether restoration of DCN expression alone is sufficient to reproduce the biological effects of SLQG requires further investigation using DCN overexpression or rescue approaches. Importantly, SLQG reduced phosphorylated Smad2/3 levels without altering total Smad2/3 abundance, suggesting that its regulatory effect on the TGF-β/Smad pathway may occur primarily through modulation of pathway activation rather than changes in Smad2/3 expression.

Transcriptomic analysis showed marked changes in tumor gene-expression profiles after SLQG treatment, with *DCN* identified as a candidate upregulated DEG. GO enrichment suggested changes in signal transduction, transcriptional regulation, and protein-interaction networks, whereas KEGG and GSEA analyses highlighted the TGF-β signaling pathway. These results provided the basis for focusing on DCN and TGF-β/Smad signaling in subsequent validation experiments.

DCN is a member of the small leucine-rich proteoglycan family and is mainly distributed in the extracellular matrix. Through its protein core and glycosaminoglycan side chains, DCN interacts with multiple growth factors and cell-surface receptors, thereby exerting broad regulatory effects within the tumor microenvironment. One important function of DCN is its ability to bind and neutralize TGF-β1. By interfering with the interaction between TGF-β and its receptors, DCN can form inactive TGF-β–DCN complexes, limit the activation of TGF-β receptor types I and II, and reduce Smad2/3 phosphorylation. DCN also antagonizes several receptor tyrosine kinases, including epidermal growth factor receptor, insulin-like growth factor-1 receptor and hepatocyte growth factor receptor, thereby affecting receptor stability, downstream signaling, proliferation, apoptosis and differentiation [17]. Previous studies in breast cancer [18], prostate cancer [19], pancreatic cancer [20] and glioma [21] models have shown that DCN-based interventions, including viral vector-mediated *DCN* gene therapy and recombinant DCN protein or core-domain administration, can suppress tumor growth or prolong survival. DCN has also been reported to exert antimetastatic activity [22]. In the present study, GEPIA-based analysis of the TCGA/GTEx ESCA cohort showed lower *DCN* expression in ESCA tumor samples than in normal samples and an association with pathological stage. Because this analysis was based on the ESCA cohort rather than an ESCC-specific dataset, these findings should be interpreted as supportive public-database evidence for *DCN* dysregulation rather than definitive evidence specifically representing ESCC. Importantly, ESCC and esophageal adenocarcinoma exhibit distinct molecular characteristics and biological behaviors; therefore, inclusion of both histological subtypes within the ESCA cohort may introduce heterogeneity and potentially obscure subtype-specific expression patterns. Future validation using independent ESCC-specific datasets, such as GEO-derived cohorts, will be valuable to further clarify the clinical relevance of DCN dysregulation in ESCC.

The TGF-β/Smad signaling pathway regulates a wide range of cellular processes and is closely associated with tumor initiation and progression. In the canonical pathway, activated TGF-β ligands bind to type II receptors, which recruit and phosphorylate type I receptors, thereby promoting Smad2/3 phosphorylation. Phosphorylated Smad2/3 associates with Smad4, translocates into the nucleus and regulates downstream gene transcription [23]. Aberrant or sustained activation of this pathway is a recognized contributor to malignant progression [24], partly by suppressing pro-apoptotic and cell-cycle-inhibitory programs and activating proliferation-associated genes [25].

Although transcriptomic analysis highlighted enrichment of the TGF-β signaling pathway, the present study focused on DCN-associated regulation of Smad2/3 phosphorylation based on the integrative prioritization strategy combining transcriptomic analysis and experimental validation. Other components within the pathway, including Smad4, inhibitory Smads, and TGF-β receptors, were not prioritized in the current integrative analysis and were therefore not further experimentally validated. Future studies incorporating broader pathway profiling may provide additional insights into the regulatory network underlying SLQG-mediated effects.

The present study built on the previously reported UHPLC-Q-Orbitrap HRMS-based chemical characterization of the same prescription, which provided the phytochemical basis for SLQG [9]. The 123 candidate compounds identified through network pharmacology represent database-derived candidates for mechanistic exploration, whereas the 46 compounds identified by UHPLC-Q-Orbitrap HRMS represent experimentally detected constituents of SLQG. Rather than attributing the pharmacological effects of SLQG to individual constituents, the present study investigated its anti-ESCC activity at the formula level by integrating network pharmacology, molecular docking, molecular dynamics simulation, transcriptomic analysis, and experimental validation. Among the top 10 predicted candidate compounds, quercetin, luteolin, ursolic acid and tanshinone IIA have been experimentally reported to exert anti-ESCC effects. Quercetin inhibits ESCC cell proliferation, invasion and angiogenesis [26]; luteolin induces cell cycle arrest and mitochondrial apoptosis in ESCC cells [27]; ursolic acid enhances paclitaxel-induced apoptosis and antitumor activity by targeting Akt/FOXM1 signaling [28]; and tanshinone IIA induces apoptosis through endoplasmic reticulum stress and the mitochondrial pathway [29]. The repeated identification of ursolic acid is noteworthy, as previous work has shown that ursolic acid upregulates DCN expression and acts through DCN-mediated IGF-1R and HIF-1 signaling [30]. However, the repeated identification of ursolic acid does not necessarily indicate that it represents the predominant active component of SLQG, given the synergistic and network-based characteristics of multi-component herbal formulations. Therefore, the pharmacological effects of SLQG should be interpreted within the context of its multi-component nature, in which multiple constituents may collectively influence diverse molecular targets and biological processes. The compounds identified through network pharmacology and computational analyses should thus be considered potential contributors and mechanistic clues rather than definitive determinants of the pharmacological activity of SLQG. It should be noted that the biological interpretation of small molecule–DCN docking requires caution. Given that DCN is an extracellular matrix proteoglycan, the present docking analysis was not intended to demonstrate direct pharmacological binding or validated target engagement between SLQG-derived compounds and DCN. Instead, it was performed as an exploratory computational approach to identify potential molecular associations that may provide mechanistic insights into SLQG-mediated regulation of DCN-related signaling. Nevertheless, these computational predictions should be interpreted as mechanistic hypotheses rather than definitive evidence of direct compound–DCN binding. Further biophysical validation will be required to determine whether these predicted interactions represent biologically relevant compound–DCN associations underlying the pharmacological effects of SLQG.

The in vivo experiments showed that SLQG reduced xenograft tumor volume and tumor weight, indicating inhibition of tumor growth. Compared with the Model group, the SLQG-L and SLQG-M groups showed less body-weight loss, suggesting that these doses were relatively well tolerated under the present short-term experimental conditions. Histological examination showed that SLQG treatment disrupted tumor tissue architecture and induced pathological changes such as nuclear pyknosis and focal necrosis. TUNEL staining and apoptosis-related markers further indicated that SLQG enhanced apoptotic signaling, as reflected by increased BAX and cleaved caspase-3 expression and decreased BCL2 expression. At the molecular level, SLQG increased DCN and p21 expression while reducing p-Smad2/3 and Cyclin D1 expression, consistent with inhibition of TGF-β/Smad signaling and induction of G_0_/G_1_ cell cycle arrest. Notably, the medium dose produced the most consistent antitumor response, whereas the high dose did not further enhance the effect, suggesting a non-linear dose–response pattern that warrants further pharmacokinetic and toxicity evaluation. Importantly, the antitumor response observed with SB431542 did not completely parallel the efficacy of SLQG treatment in vivo. Although SB431542 effectively reduced p-Smad2/3 expression and Cyclin D1 levels in xenograft tissues, it produced only a limited tumor growth inhibitory effect under the present experimental conditions. This discrepancy suggests that direct pharmacological blockade of TGF-β receptor signaling alone may not fully reproduce the biological effects induced by SLQG. Several factors may contribute to this difference. First, the pharmacodynamic profile, tissue exposure, and duration of action of SB431542 in vivo may influence its ability to achieve sustained pathway inhibition within tumor tissues. Second, TE-1 xenograft growth may be regulated by multiple signaling networks, and inhibition of TGF-β/Smad signaling alone may be insufficient to markedly suppress tumor progression in this model. Notably, DCN, identified as a key molecule induced by SLQG, is not only a regulator of TGF-β activity but also interacts with multiple receptor tyrosine kinase pathways, including EGFR, IGF-1R, and MET signaling, which are involved in tumor proliferation, survival, and progression [17]. Therefore, the antitumor effects of SLQG should be interpreted as the consequence of coordinated regulation of DCN-associated signaling networks, with TGF-β/Smad inhibition representing an important but not exclusive component of its mechanism. The non-linear dose–response relationship observed between SLQG-M and SLQG-H also warrants careful interpretation. Although the high-dose group did not achieve greater tumor inhibition than the medium-dose group, this finding does not necessarily indicate the presence of systemic toxicity caused by SLQG. In the present study, body-weight reduction was also observed in the Model, DDP, and SB431542 groups, suggesting that changes in body weight may reflect the complex physiological consequences of tumor burden and treatment intervention rather than being solely attributable to SLQG administration. Several pharmacological factors, including exposure–response relationships and interactions among multiple constituents, may contribute to this non-linear response. Therefore, the bell-shaped dose–response pattern observed in this short-term xenograft study should be interpreted cautiously. Further pharmacokinetic and toxicological investigations are required to clarify the relationship between SLQG exposure, biological response, and safety profiles.

In vitro, SLQG-containing serum reduced TE-1 cell viability. EdU staining further showed that SLQG-containing serum suppressed DNA synthesis and proliferative capacity, suggesting that the growth inhibition observed in the CCK-8 assay was not merely attributable to reduced metabolic activity. Flow cytometry showed increased apoptosis and G_0_/G_1_ cell cycle arrest after SLQG-containing serum treatment. Consistently, western blotting showed increased DCN, BAX and p21 expression and decreased p-Smad2/3, BCL2 and Cyclin D1 expression in TE-1 cells. These findings were consistent with the transcriptomic and in vivo results, supporting the involvement of DCN upregulation and TGF-β/Smad inhibition.

To further examine the functional contribution of DCN, siRNA-mediated *DCN* knockdown was performed in TE-1 cells. Under *DCN* knockdown conditions, the effects of SLQG-containing serum on p-Smad2/3, BAX, and Cyclin D1 were attenuated, supporting the contribution of DCN to the antitumor activity of SLQG. However, the incomplete reversal of these molecular changes after *DCN* silencing indicates that DCN is unlikely to function as the sole mediator of SLQG activity. Given the multi-component and multi-target characteristics of herbal formulations, SLQG may exert its antitumor effects through coordinated regulation of DCN-associated pathways and additional molecular networks. This interpretation is also consistent with the network pharmacology analysis, which identified multiple candidate compounds, including quercetin, luteolin, ursolic acid, and tanshinone IIA, with reported antitumor activities. Therefore, DCN should be considered an important regulatory node involved in SLQG-mediated ESCC inhibition rather than the only determinant of its pharmacological effects. Future studies using gain-of-function approaches, such as *DCN* overexpression, and more comprehensive pathway analyses are warranted to further define the contribution of DCN and related signaling networks.

This study has several limitations. First, although transcriptome sequencing provided an important basis for identifying candidate molecular changes associated with SLQG treatment, the initial DEG screening was performed using nominal *P* values without incorporation of multiple-testing correction. Therefore, the possibility of false-positive findings cannot be completely excluded. However, DCN was not interpreted solely based on transcriptomic screening; rather, its biological relevance was further supported by consistent findings from public-database analysis, in vivo validation, in vitro experiments, and DCN knockdown assays. Future studies using larger transcriptomic cohorts and stricter FDR-controlled analyses will further validate the transcriptomic signatures associated with SLQG treatment. Network pharmacology, molecular docking and molecular dynamics simulations provided hypothesis-generating evidence for potential compound–target associations but did not demonstrate direct target engagement. Further validation using surface plasmon resonance, cellular thermal shift assays, pull-down assays or related biophysical methods is needed. In addition, the present study used a subcutaneous nude mouse xenograft model and a single TE-1 cell line, which cannot fully recapitulate the immune contexture, histological heterogeneity or organ-specific microenvironment of clinical ESCC. Although these models, together with SLQG-containing serum treatment and *DCN* knockdown experiments, provided mechanistic evidence supporting the involvement of DCN-associated regulation, the findings remain limited to preclinical models. Validation in additional ESCC cell lines, orthotopic or immunocompetent models, patient-derived organoids and clinical tumor specimens with comprehensive molecular and pathological information would further strengthen the translational relevance of the findings. Although *DCN* knockdown supported the functional involvement of DCN, *DCN* overexpression or rescue experiments were not performed; therefore, the extent to which the effects of SLQG depend on DCN remains to be clarified. Moreover, because GEPIA does not provide an ESCC-specific query option, the ESCA cohort was used for public-database analysis, and these data should be viewed as exploratory evidence rather than definitive ESCC-specific clinical validation. Finally, the non-linear dose–response pattern observed in vivo requires further pharmacokinetic, exposure–response and safety studies.

## 5. Conclusions

This study suggests that SLQG inhibits ESCC progression in TE-1 xenograft and cell-based models. Mechanistically, SLQG was associated with increased DCN expression, attenuation of Smad2/3 phosphorylation, enhanced apoptosis, and induction of G_0_/G_1_ cell cycle arrest. *DCN* knockdown partially attenuated these effects, supporting the involvement of DCN-associated signaling regulation in the biological response to SLQG. These findings provide preclinical evidence that DCN-associated signaling networks, including modulation of TGF-β/Smad pathway activation, may contribute to the anti-ESCC effects of SLQG.

## Supporting information

S1 File Supplementary figures and tables. This file includes S1 Fig, showing representative docking conformations of DCN with the remaining seven major active compounds of SLQG; S2 Fig, showing solvent-accessible surface area analysis of DCN–ligand complexes during 100-ns molecular dynamics simulations; S1 Table, listing the previously identified constituents of SLQG based on UHPLC-Q-Orbitrap HRMS analysis; S2 Table, listing primer sequences used for apoptosis-related efficacy validation assays; S3 Table, listing primer sequences used for DCN/TGF-β/Smad pathway validation assays; S4 Table, listing siRNA and primer sequences used for *DCN* knockdown experiments; S5 Table, listing predicted candidate compounds of SLQG identified by network pharmacology; and S6 Table, listing molecular docking energy values of the top 10 candidate compounds with DCN.
(DOCX)

S1 Raw images Original uncropped and minimally adjusted Western blot images. This file includes the original Western blot images corresponding to the cropped Western blot panels shown in Figs 4, 5, and 7.
(PDF)

S1 Data Underlying numerical data and statistical results. This file includes the individual data points and statistical results supporting the quantitative graphs and Table 1.
(XLSX)
