# Supplementary material for: Shenling Qige Granules inhibit esophageal squamous cell carcinoma progression in TE-1 xenograft and cell-based models by modulating the DCN–TGF-β/Smad signaling axis
Source: PLoS One. 2026 Oct 6;21(10):e0359405. doi: 10.1371/journal.pone.0359405 (PMC13641691; doi:10.1371/journal.pone.0359405)

Fig. 4E–H

BAX-1

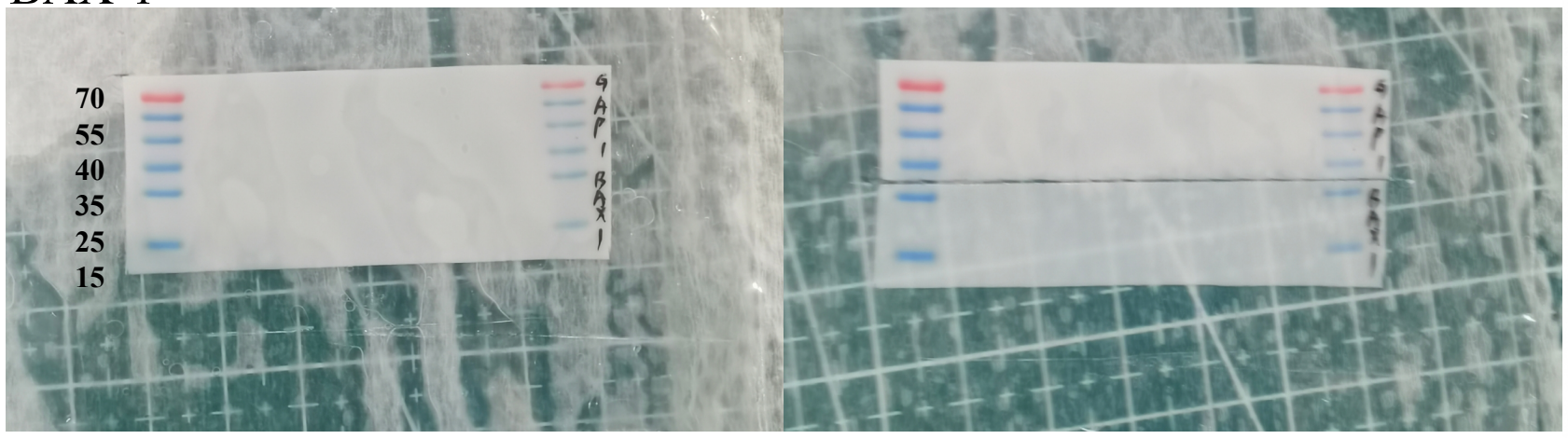

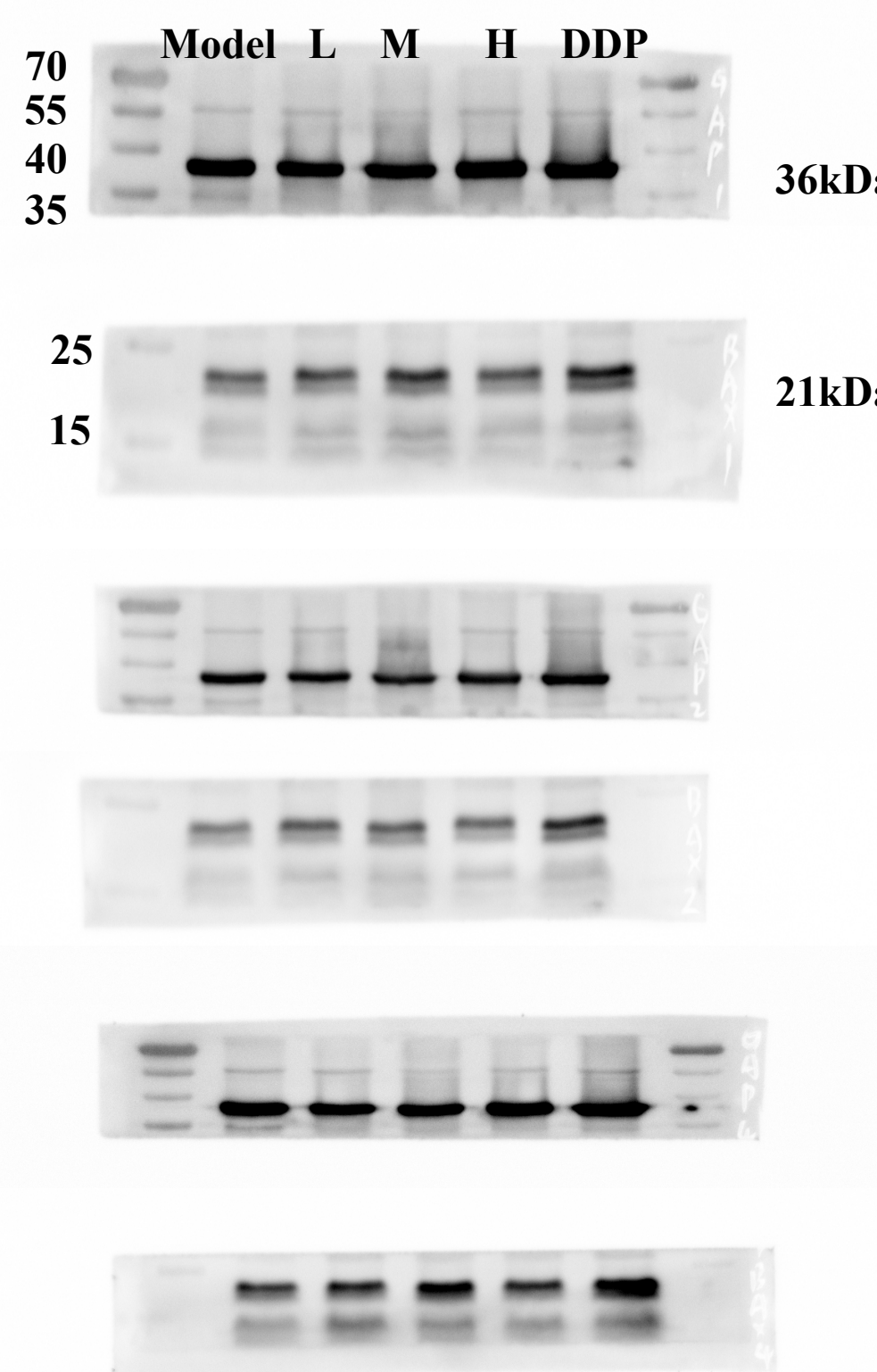

BAX-2

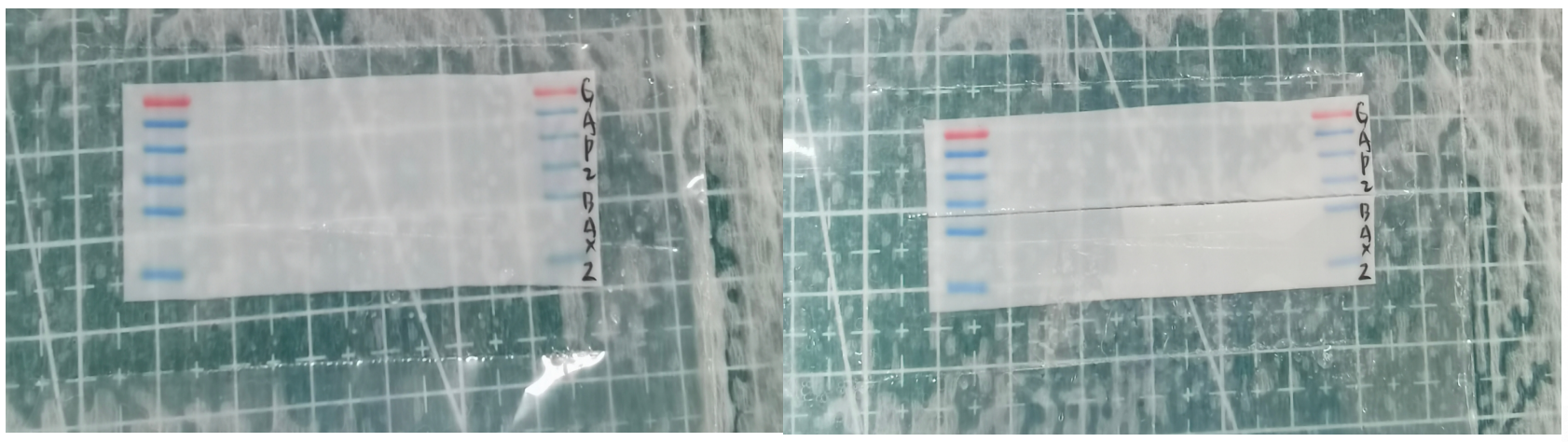

BAX-3

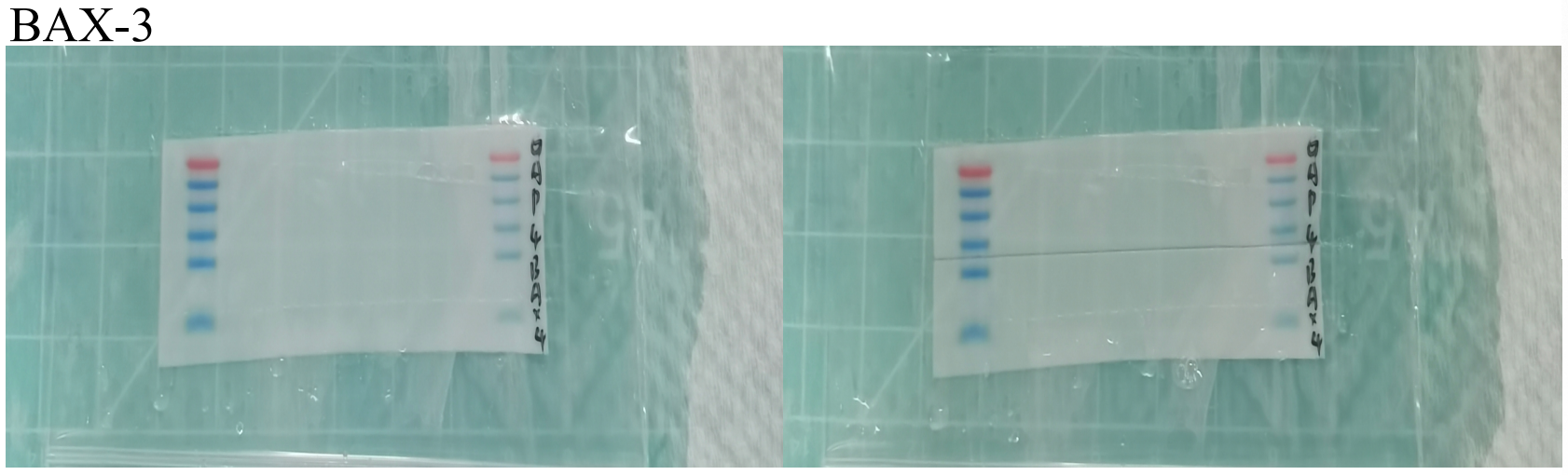

BCL2-1

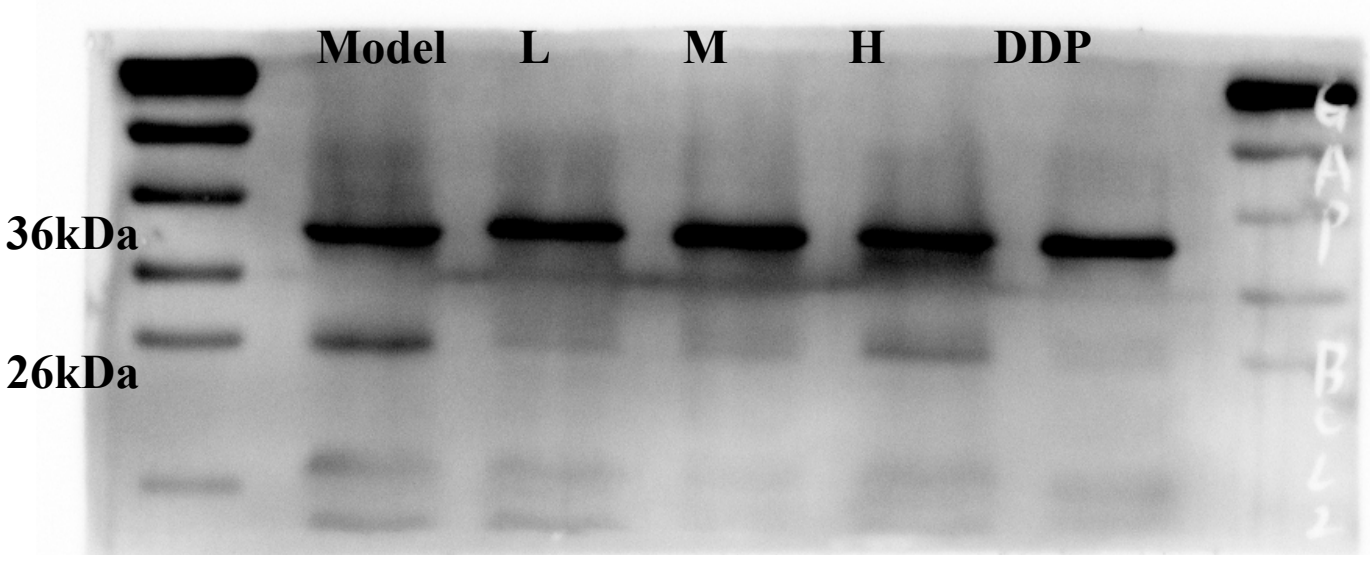

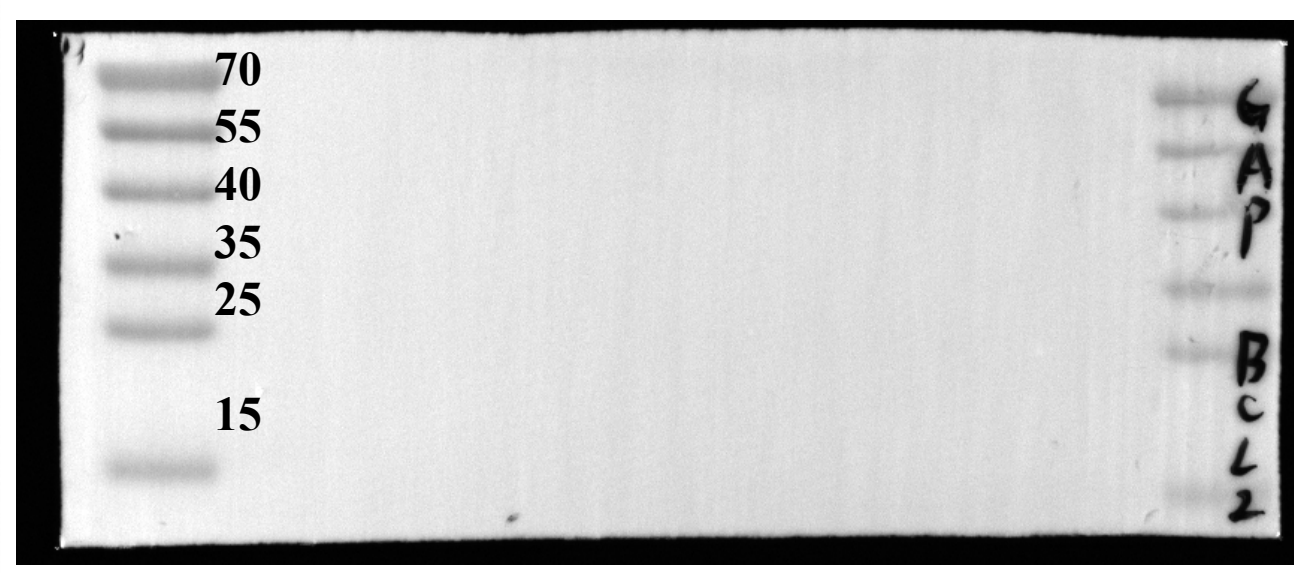

BCL2-2

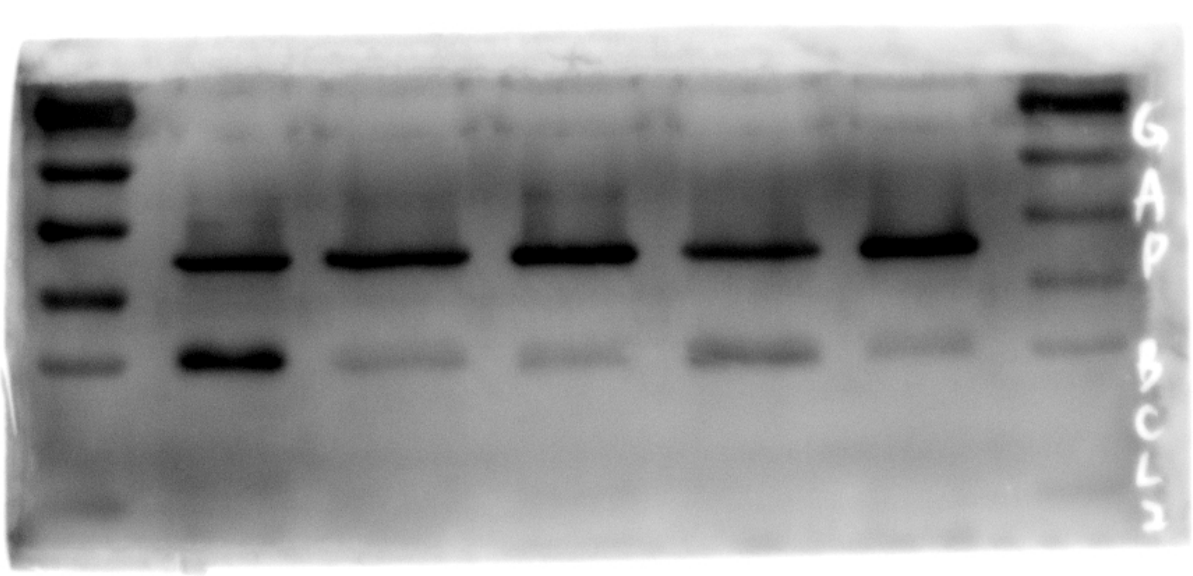

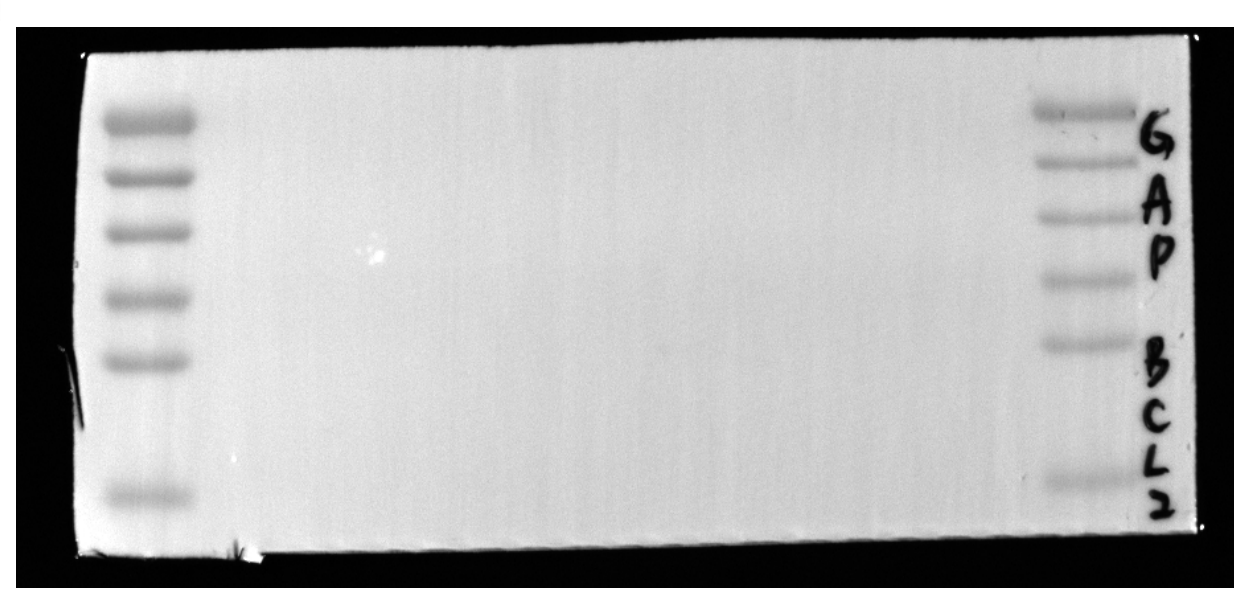

BCL2-3

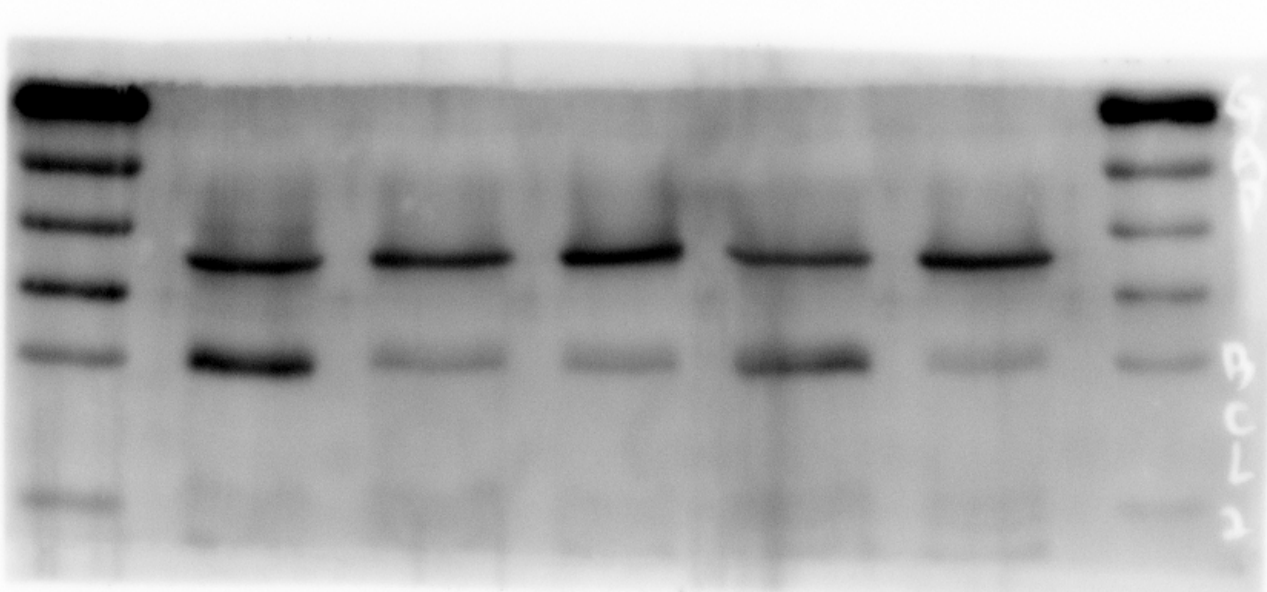

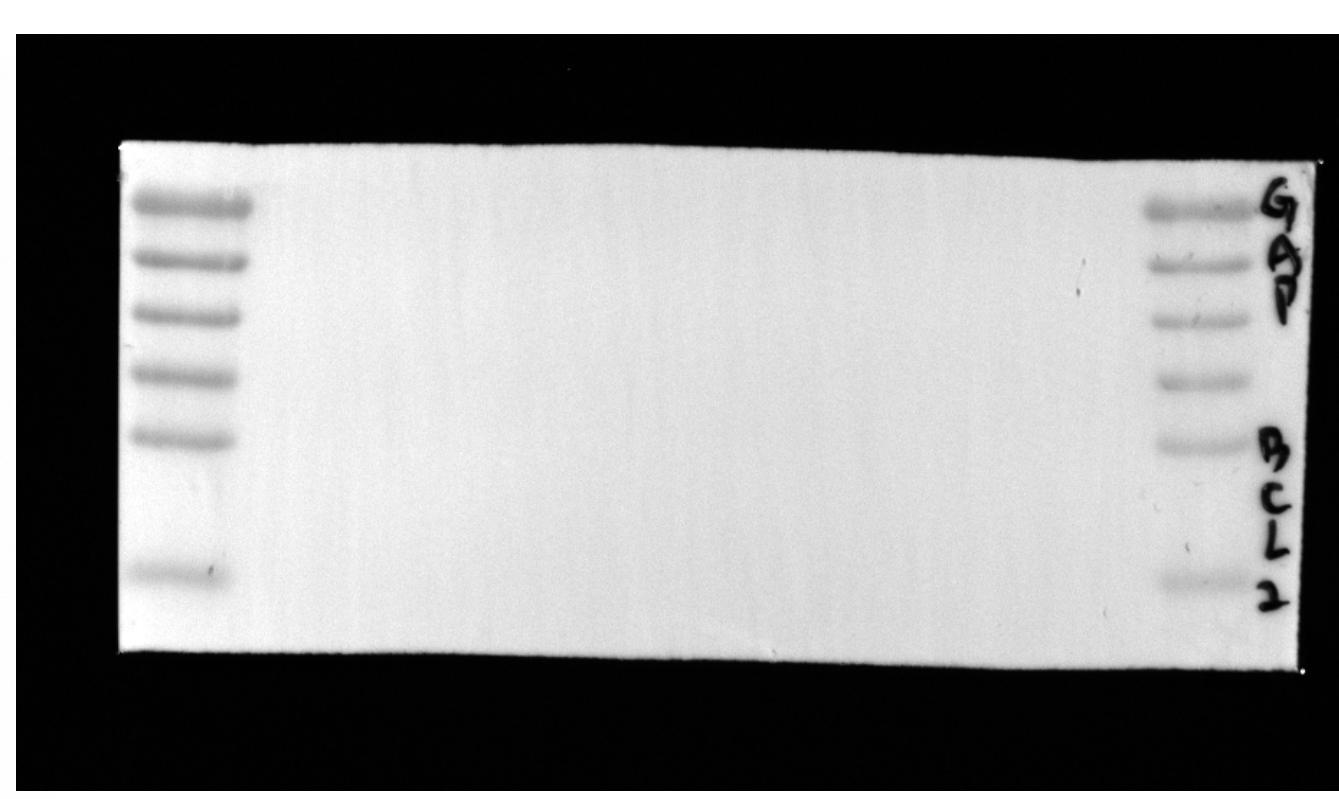

Fig. 4E–H

cleaved caspase-3-1

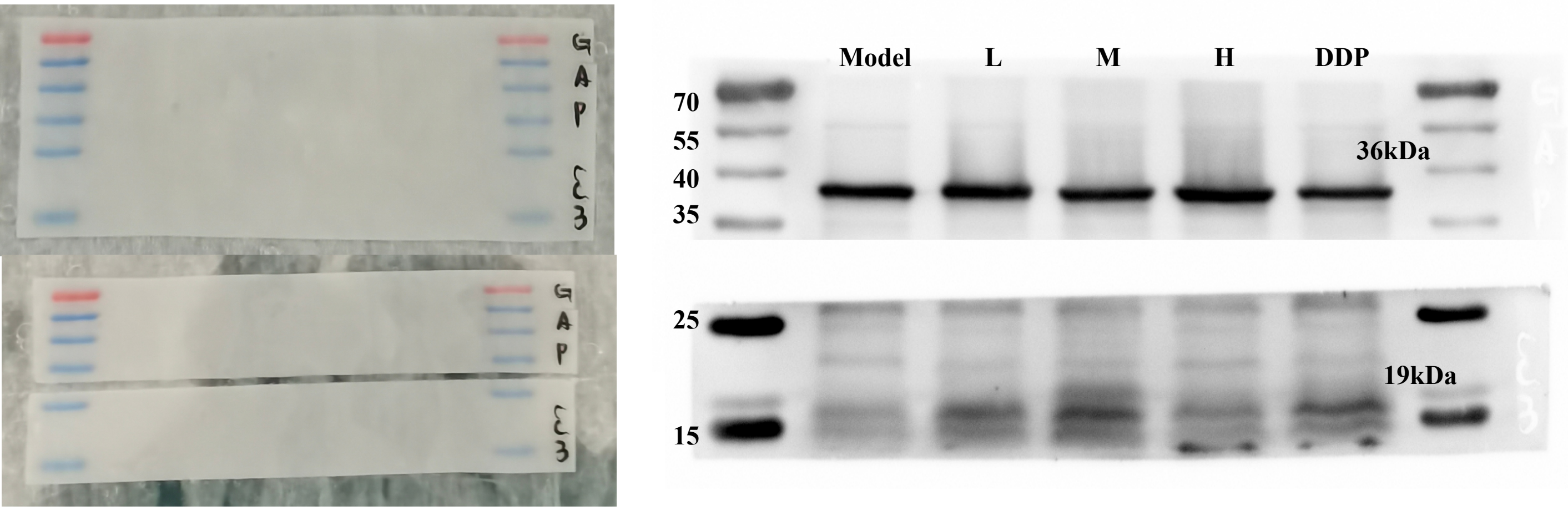

cleaved caspase-3-2

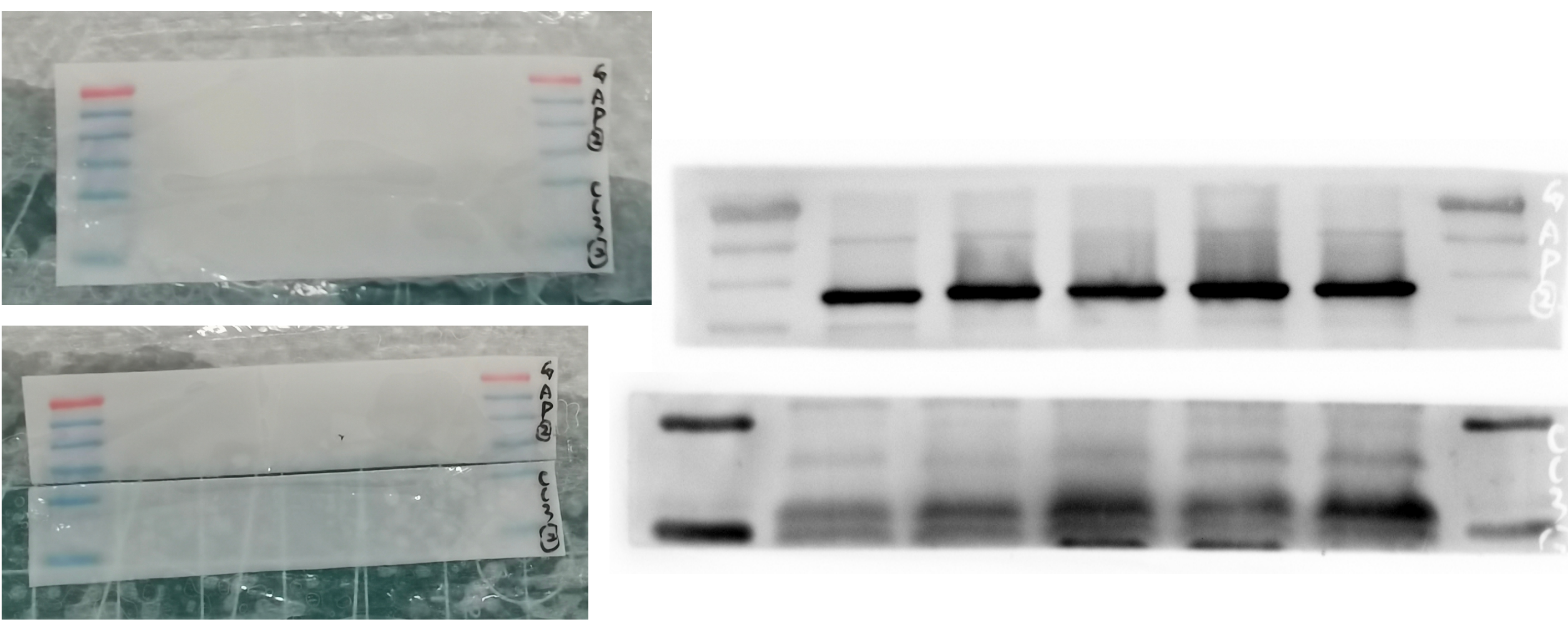

cleaved caspase-3-3

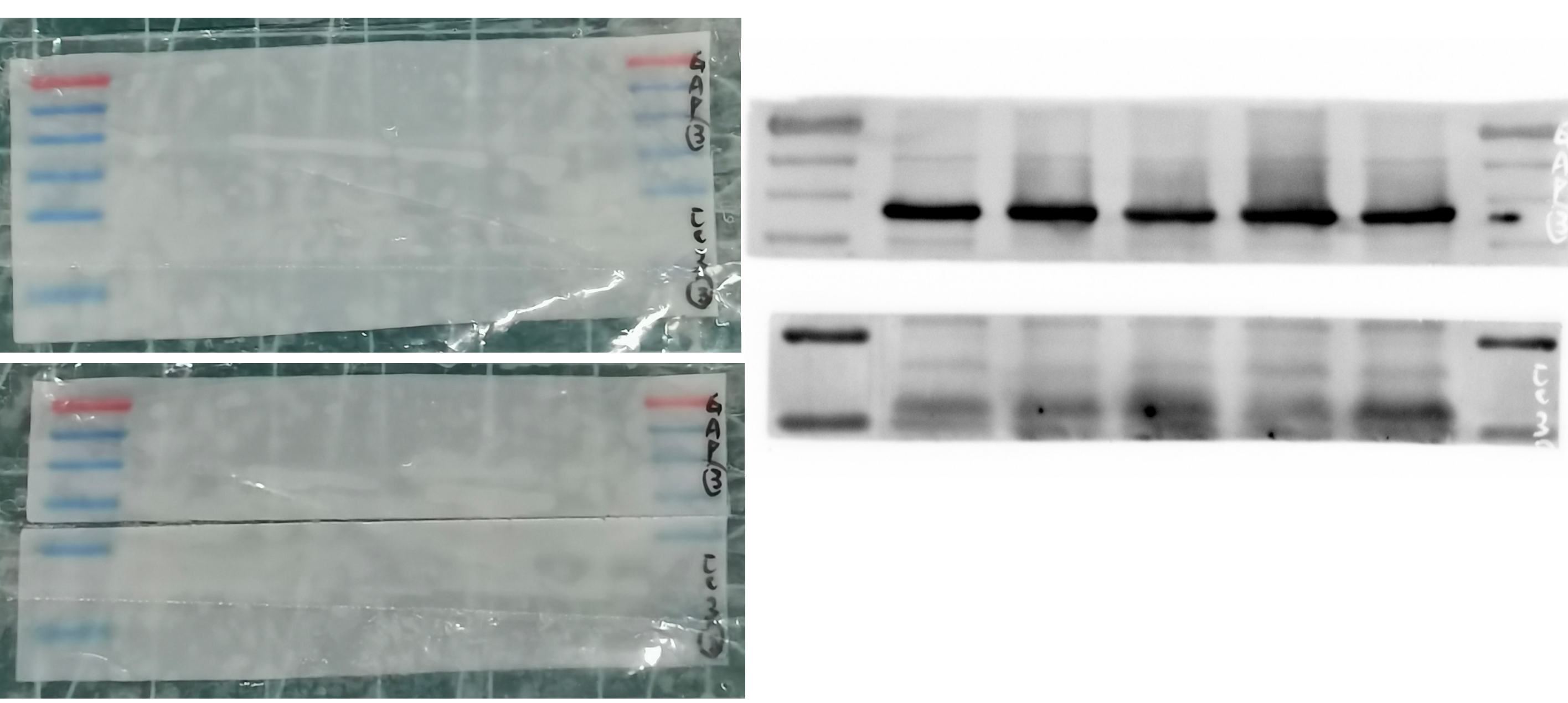

Fig. 5H–M

DCN-1

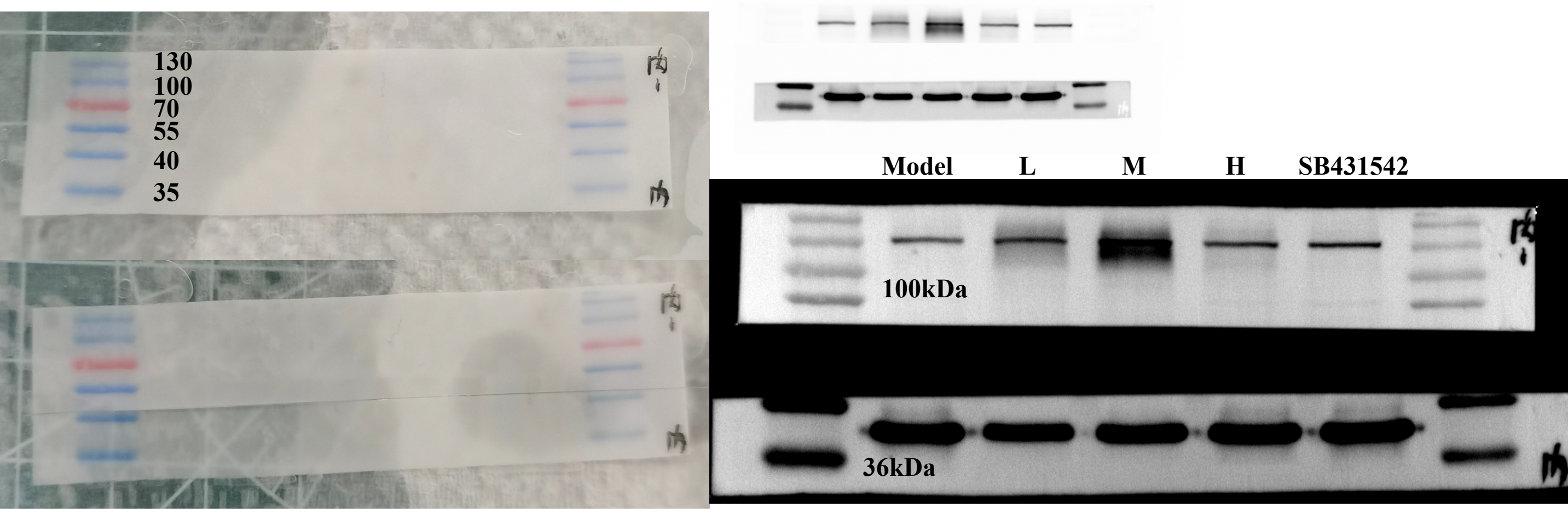

DCN-2

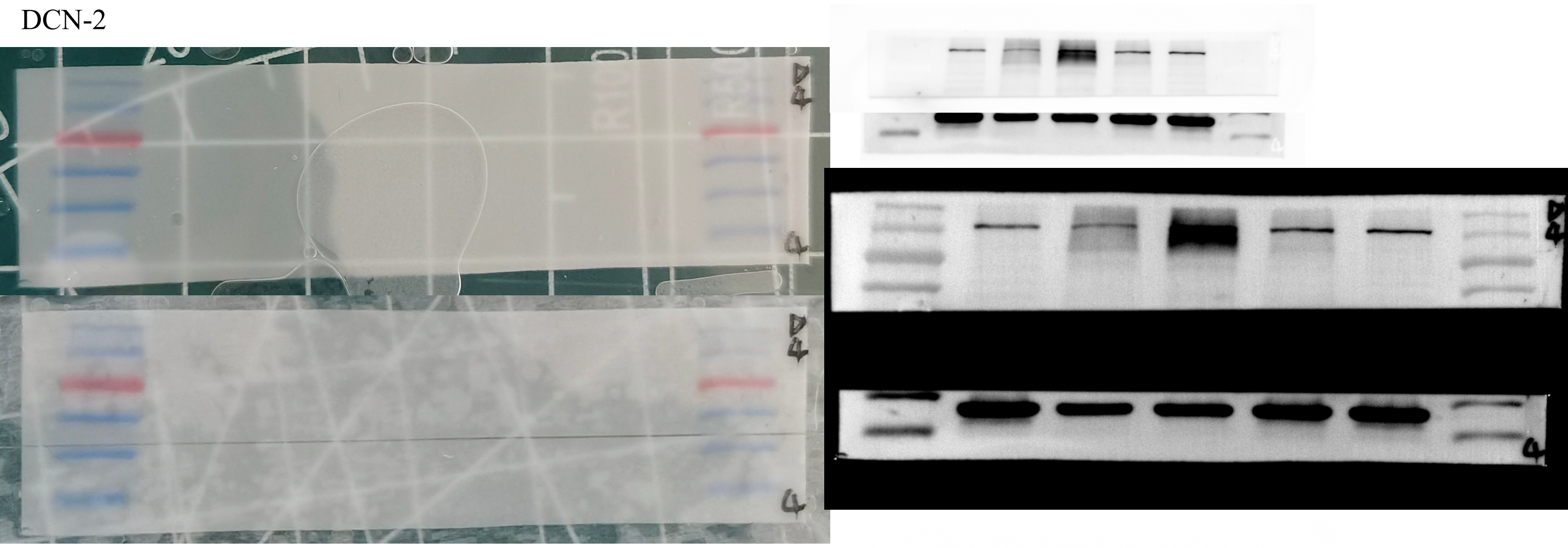

DCN-3

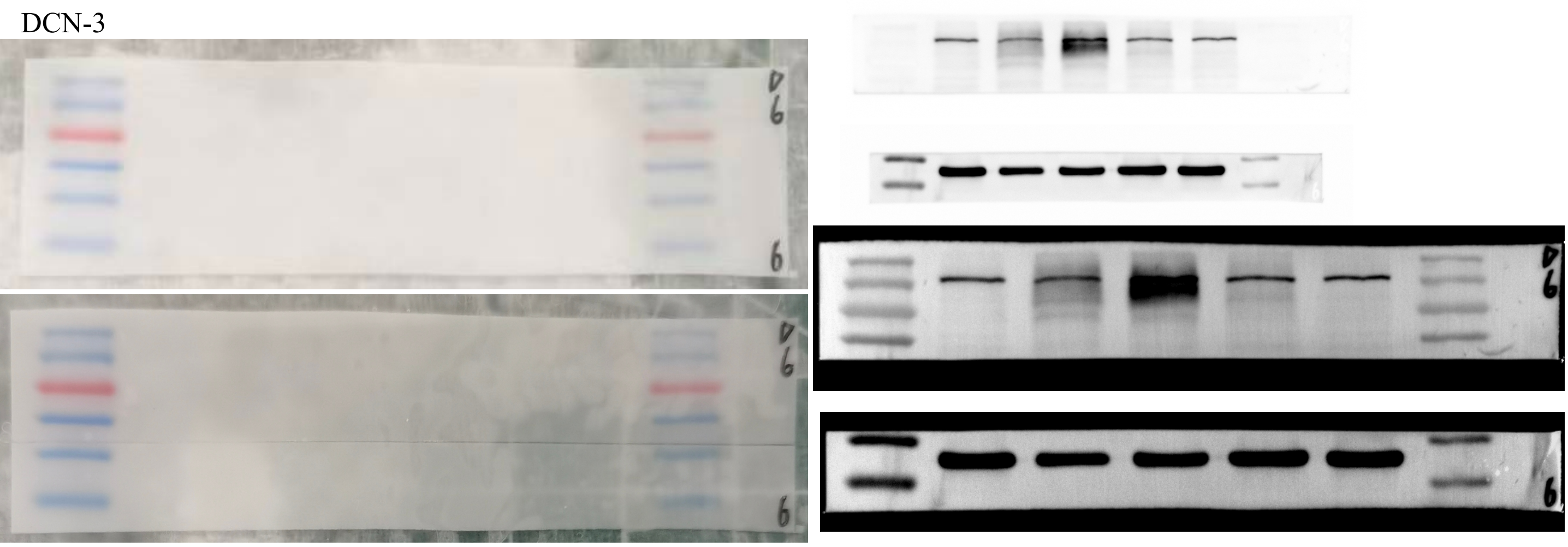

Fig. 5H–M

Smad2/3-1

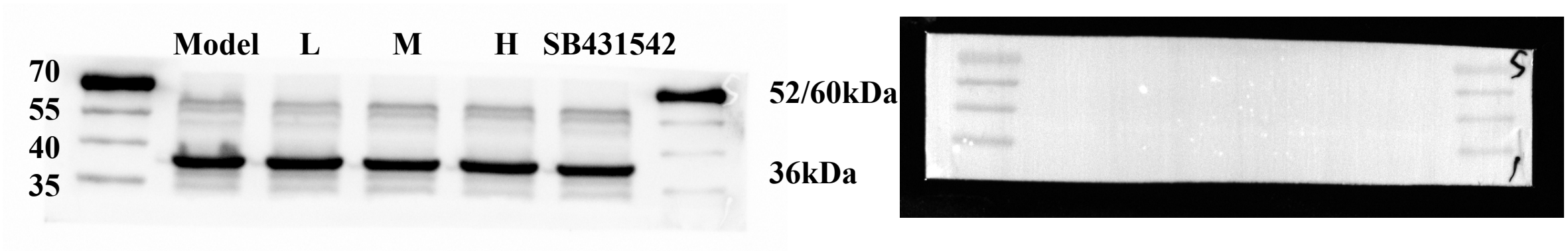

Smad2/3-2

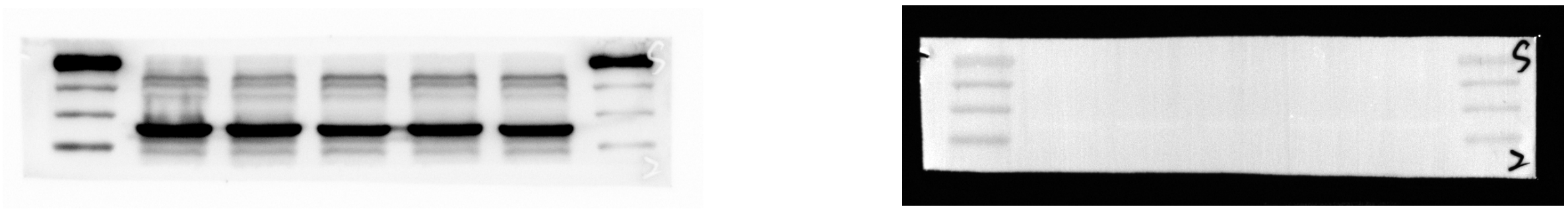

Smad2/3-3

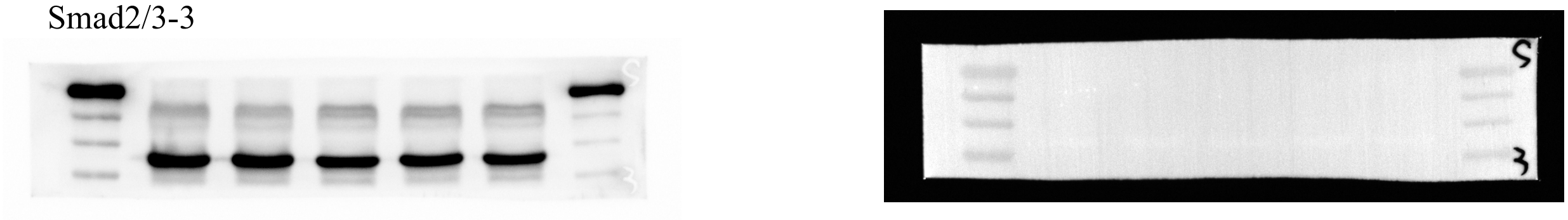

p-Smad2/3-1

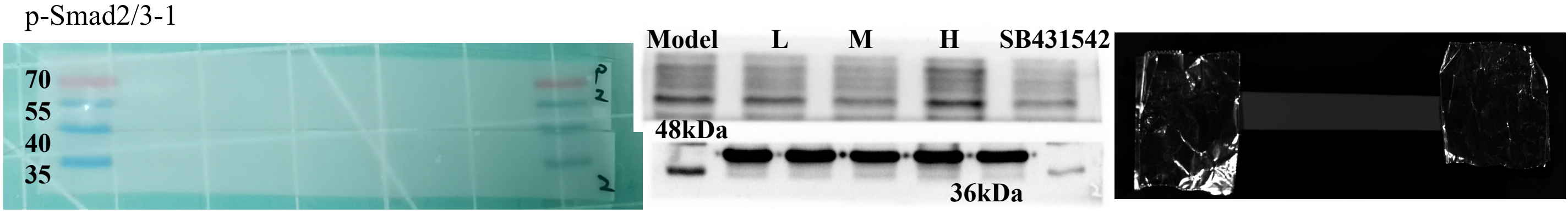

p-Smad2/3-2

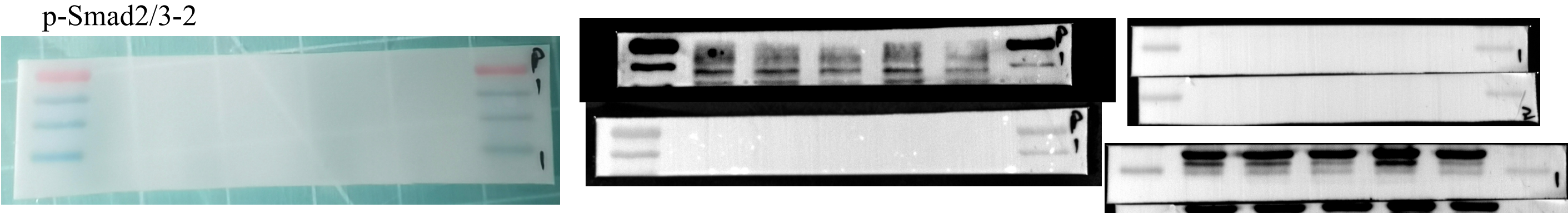

p-Smad2/3-3

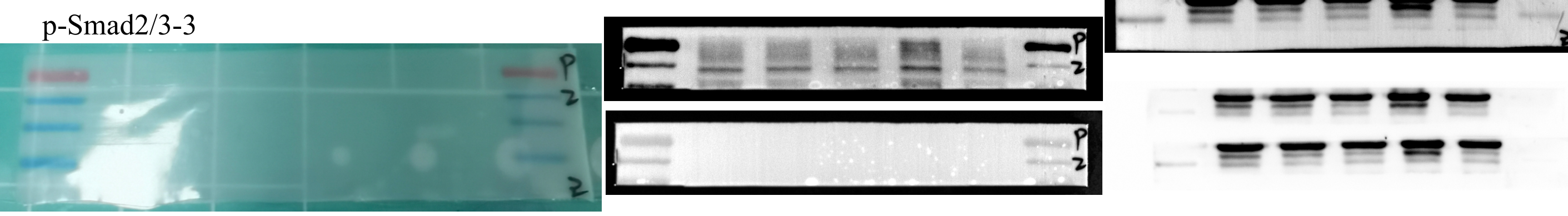

Fig. 5H–M

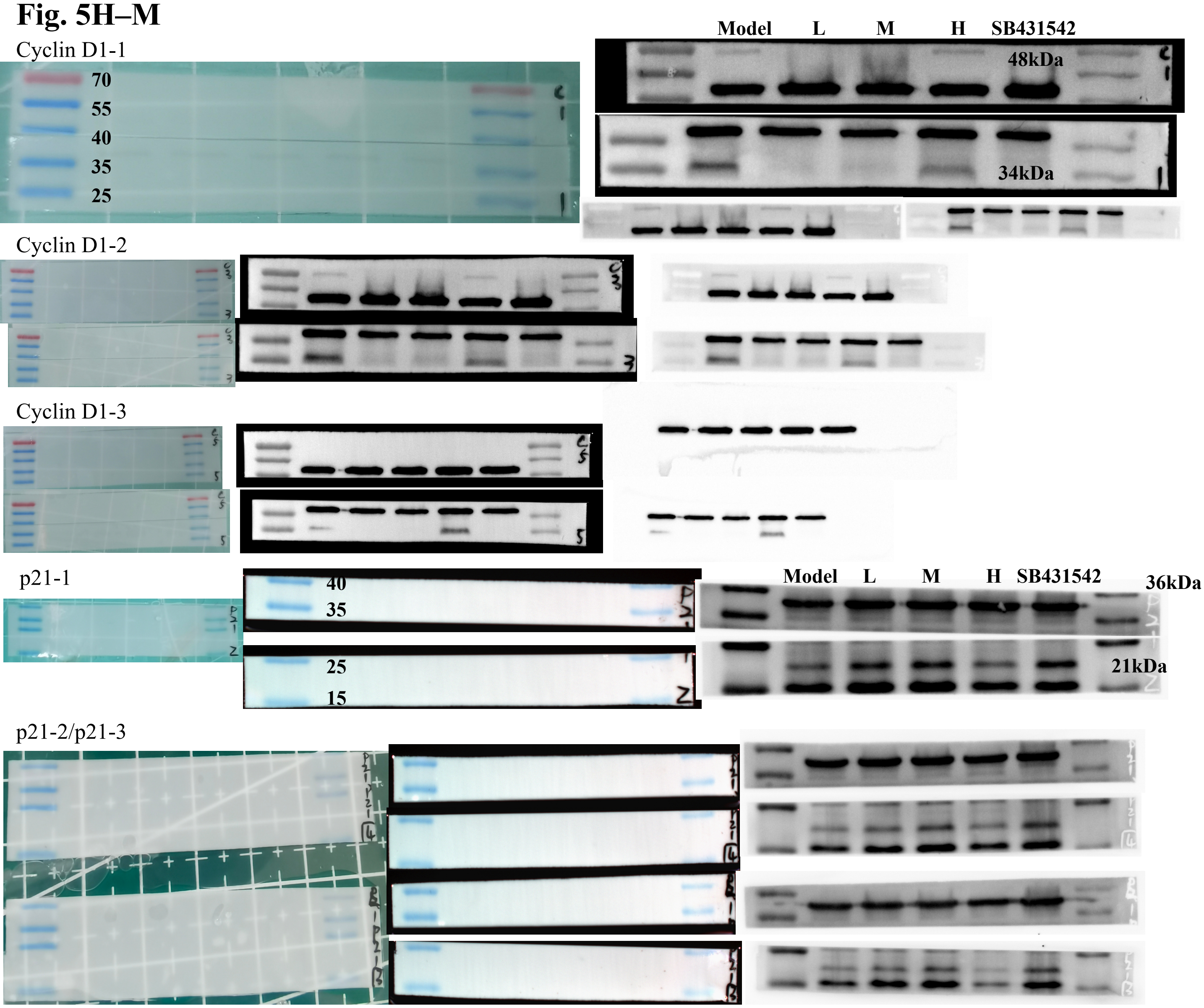

Cyclin D1-1
70
55
40
35
25
Model
L
M
H
SB431542
48kDa
34kDa
Cyclin D1-2
Cyclin D1-3
p21-1
40
35
25
15
Model
L
M
H
SB431542
36kDa
21kDa
p21-2/p21-3

Fig. 7A–H

DCN-1

Control、SLQG-5%、SLQG-15%、SB431542

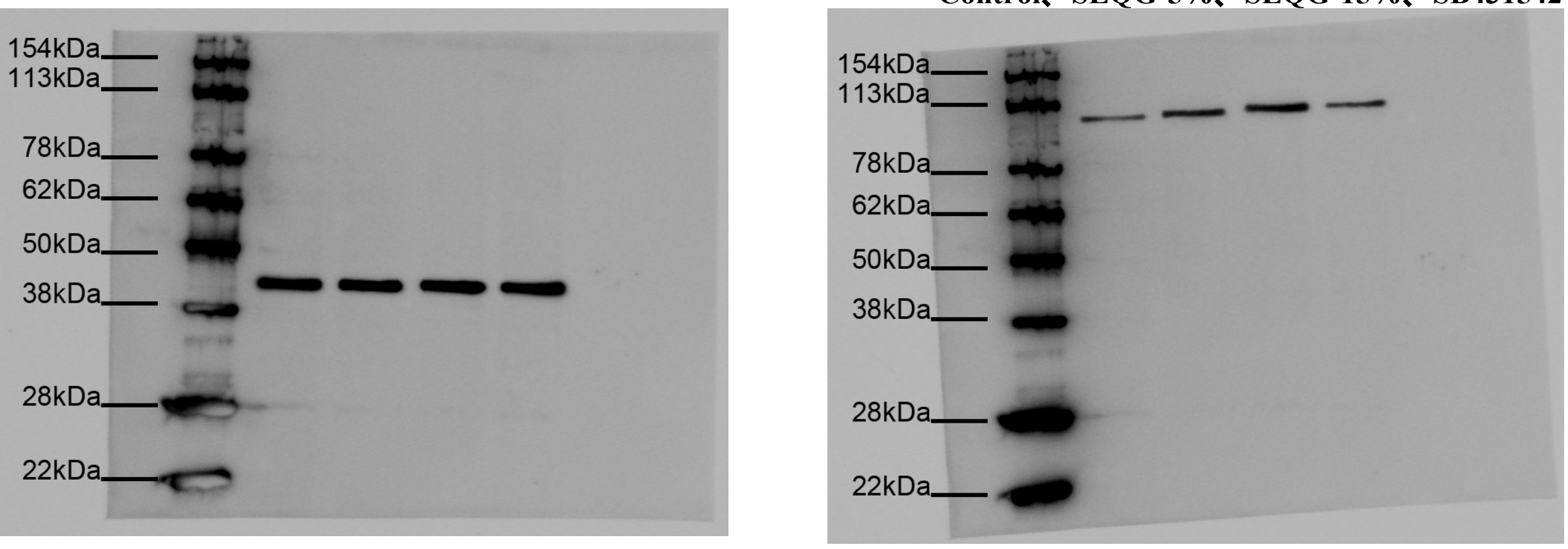

DCN-2

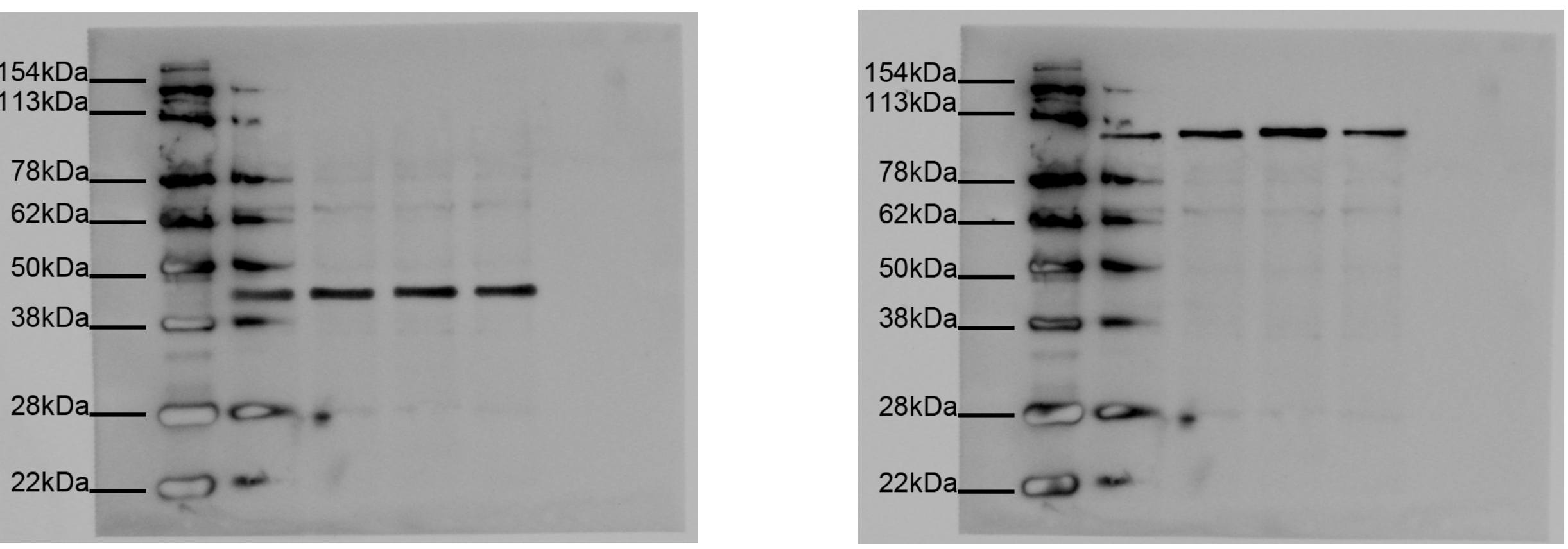

DCN-3

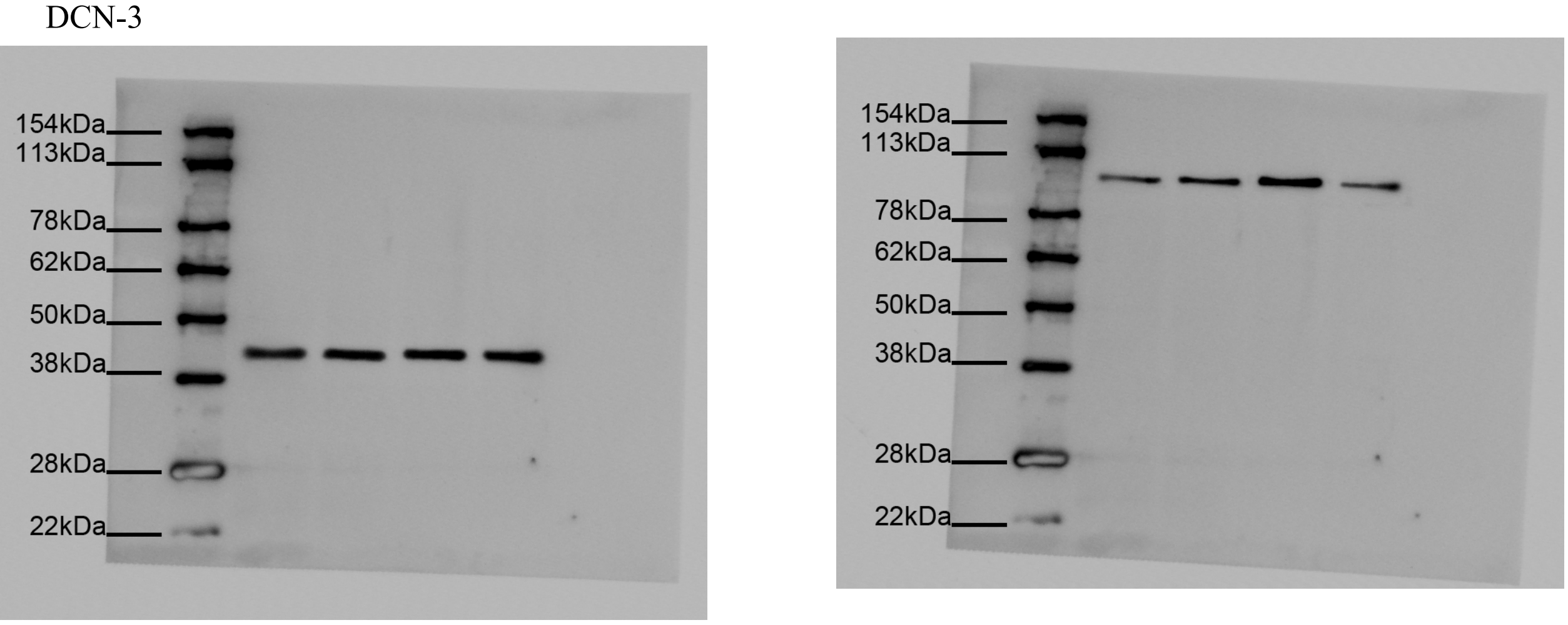

Fig. 7A–H

Smad2/3-1

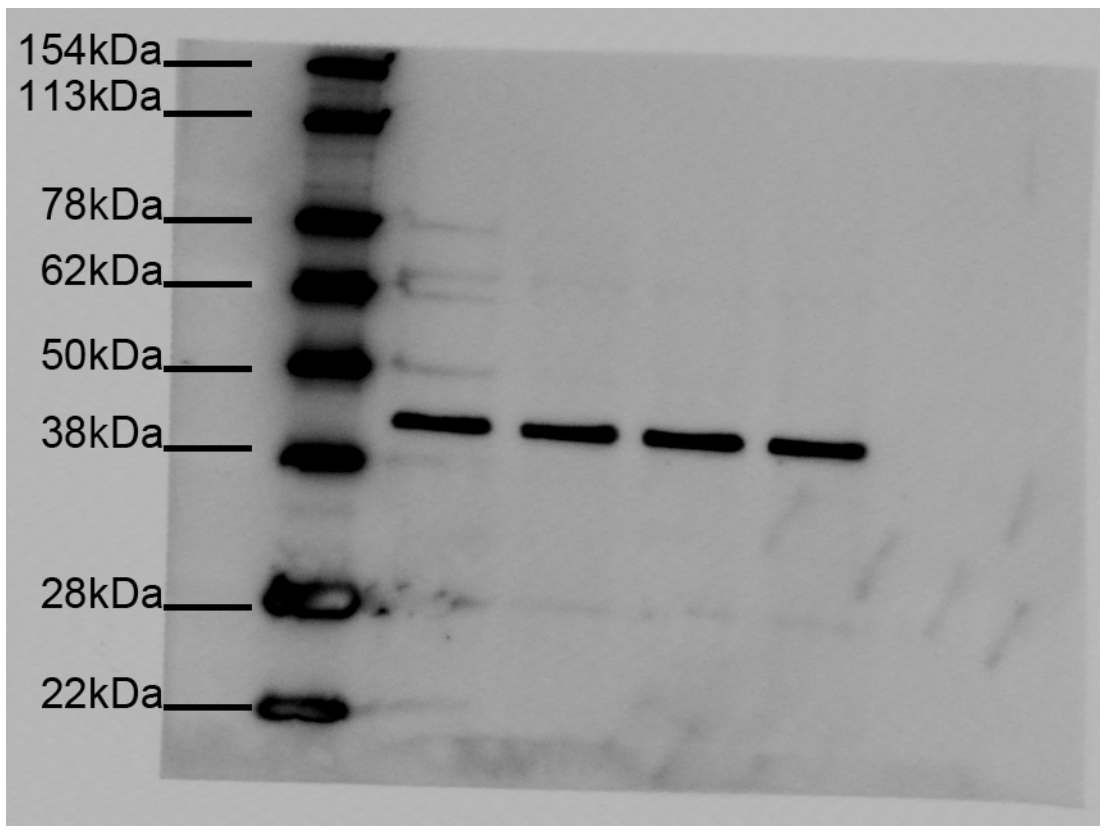

Control、SLQG-5%、SLQG-15%、SB431542

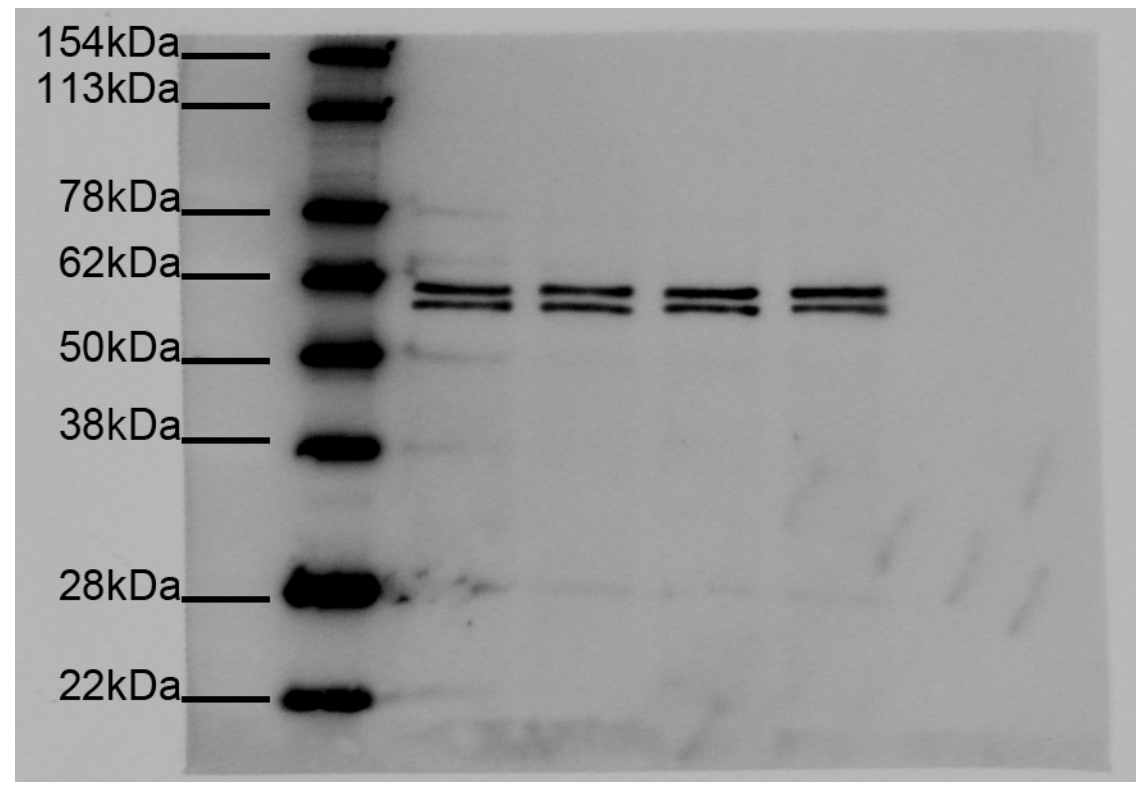

Smad2/3-2

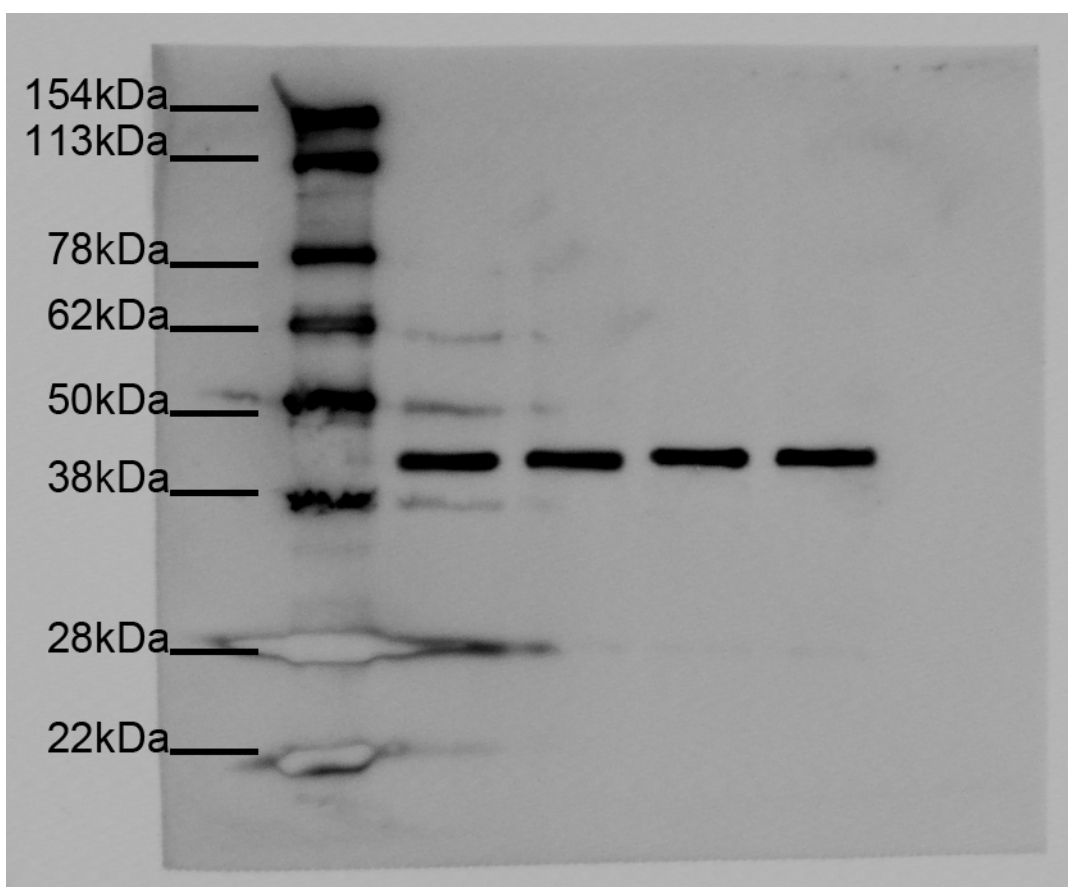

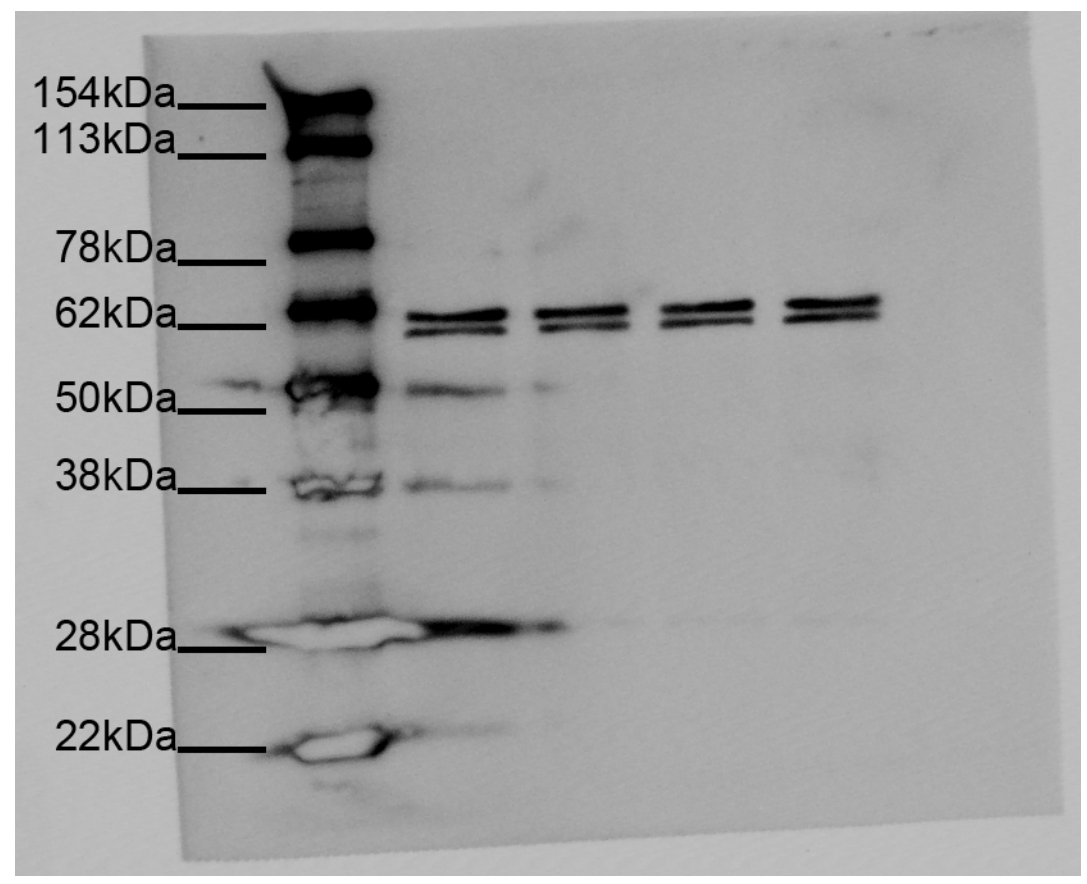

Smad2/3-3

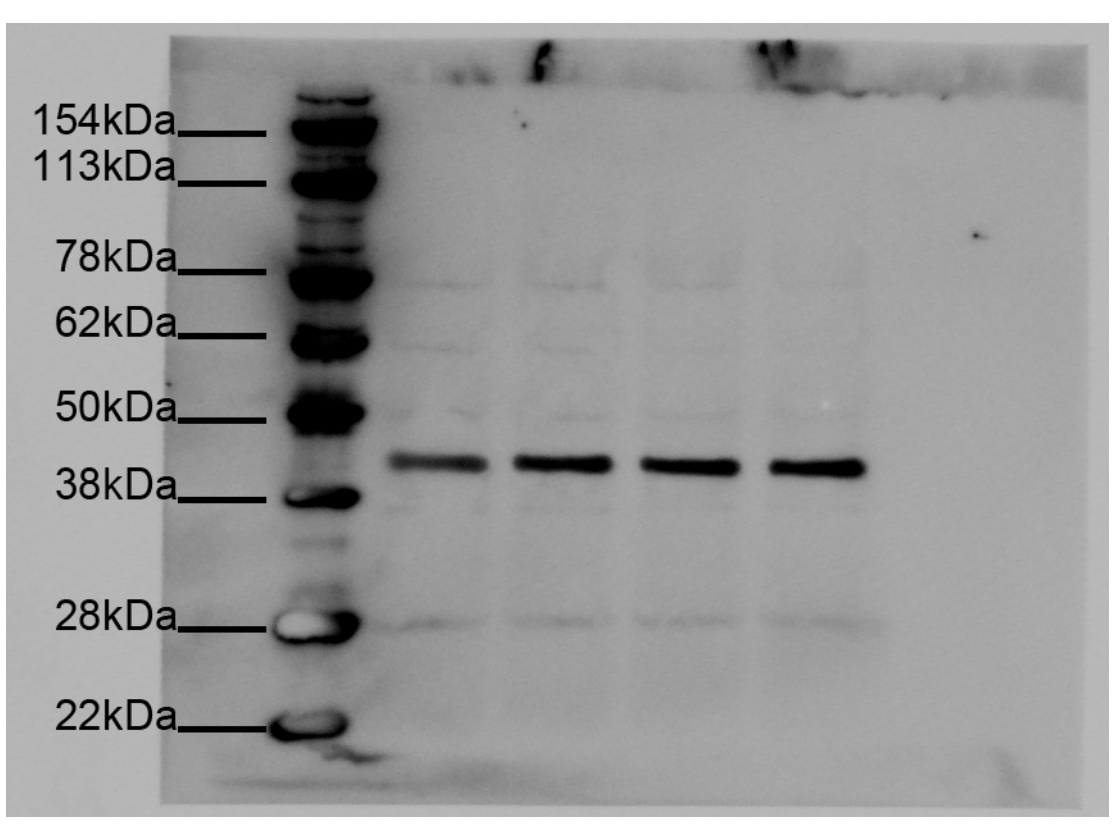

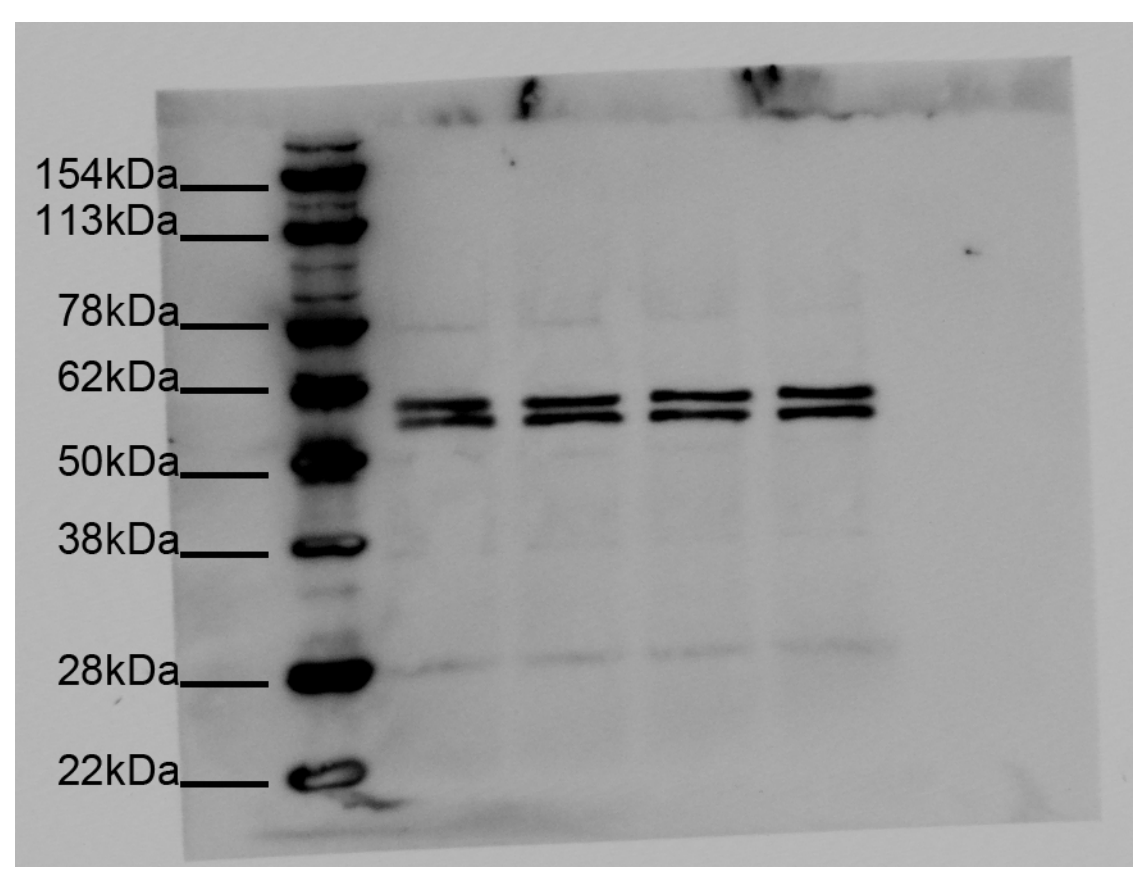

Fig. 7A–H

p-Smad2/3-1

Control、SLQG-5%、SLQG-15%、SB431542

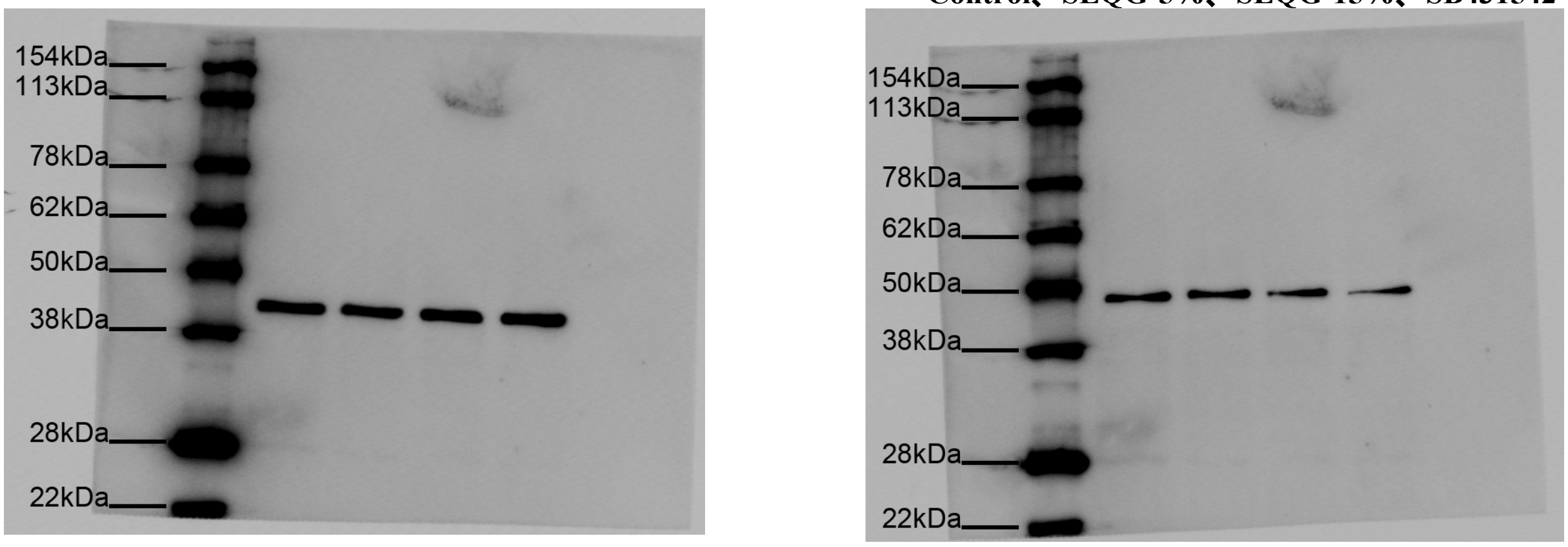

p-Smad2/3-2

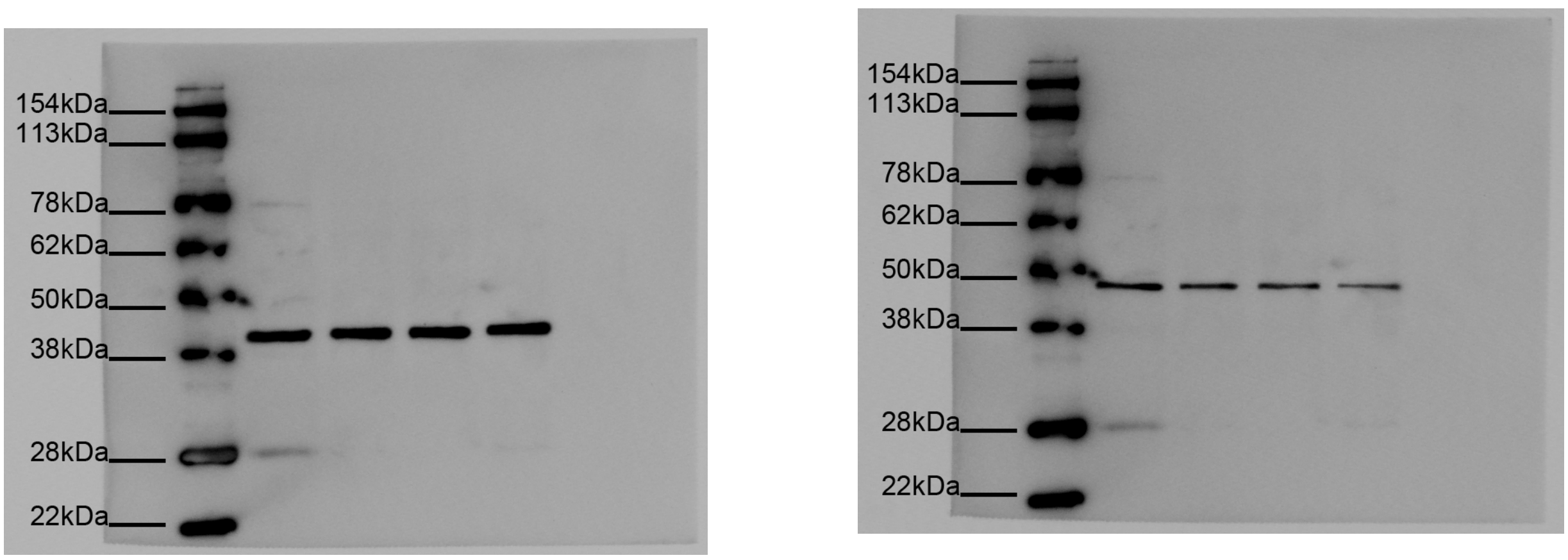

p-Smad2/3-3

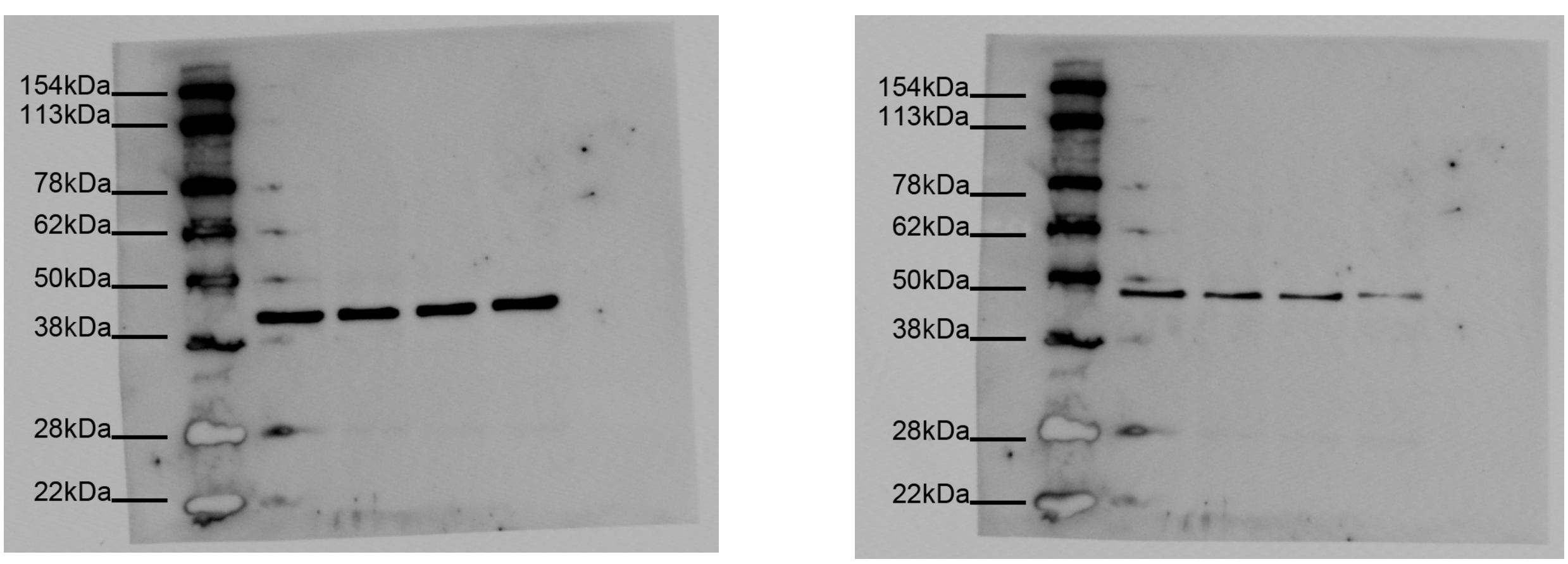

Fig. 7A–H

BAX-1

Control、SLQG-5%、SLQG-15%、SB431542

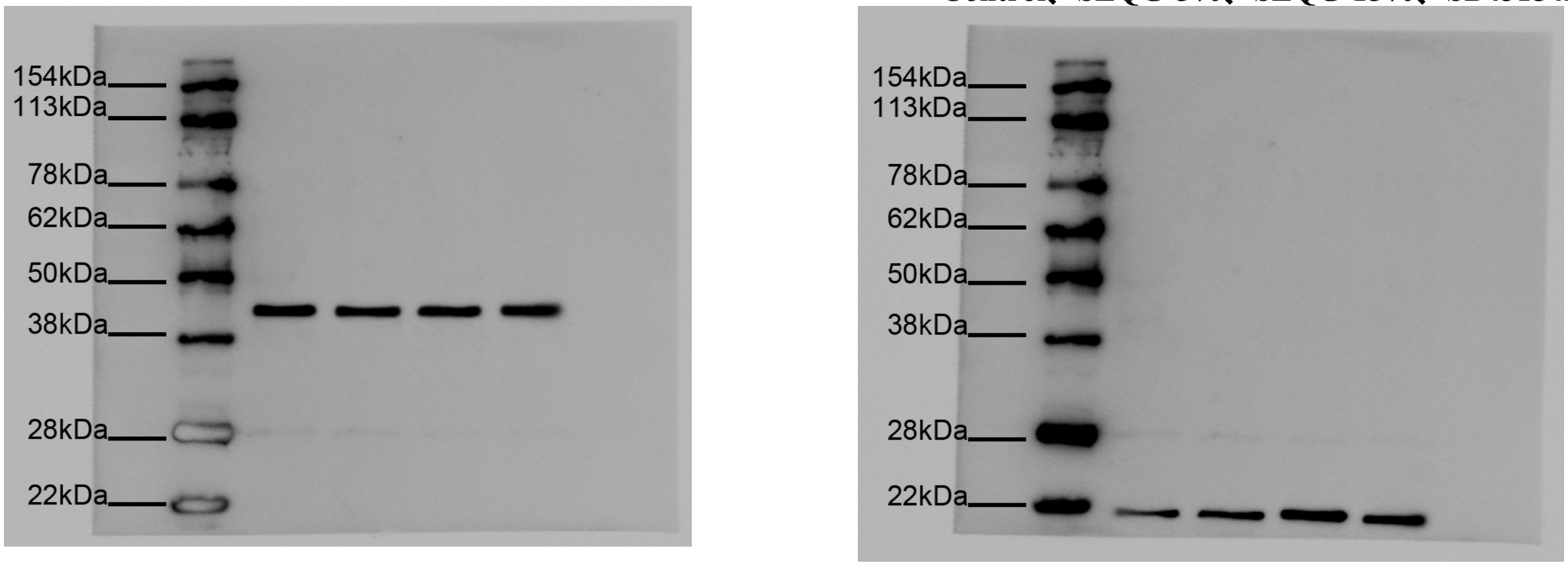

BAX-2

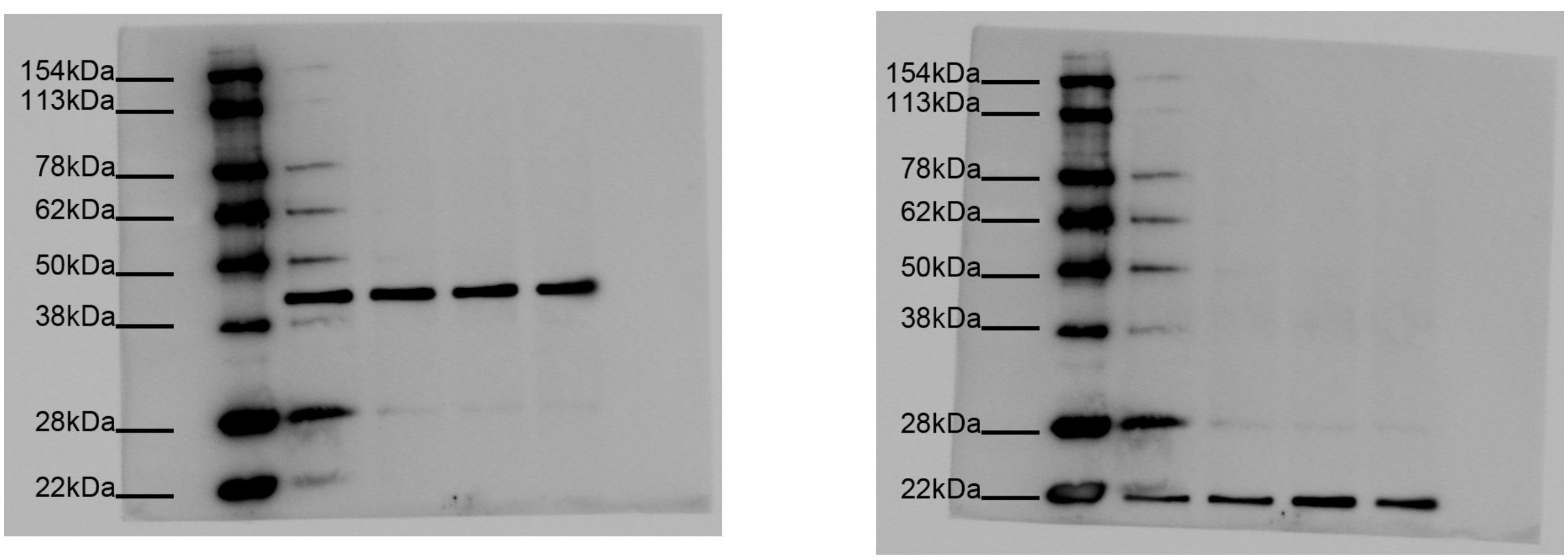

BAX-3

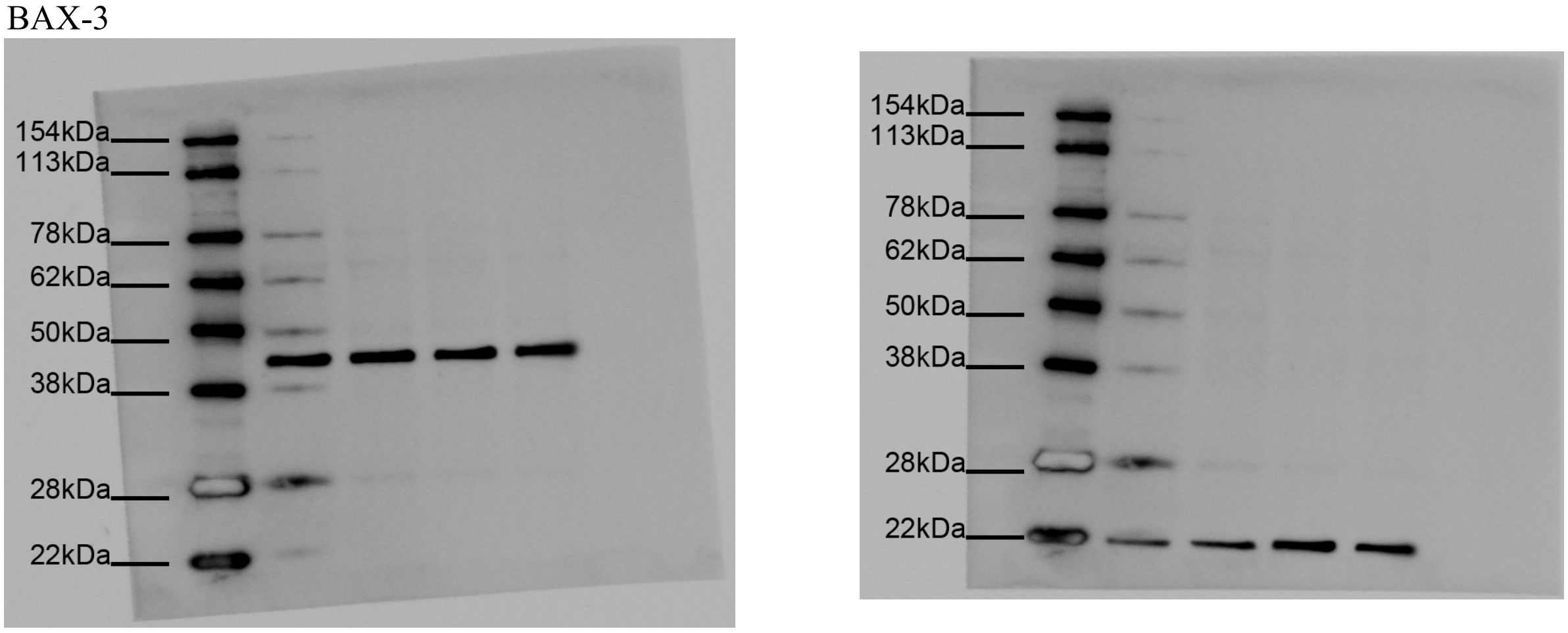

Fig. 7A–H

BCL2-1

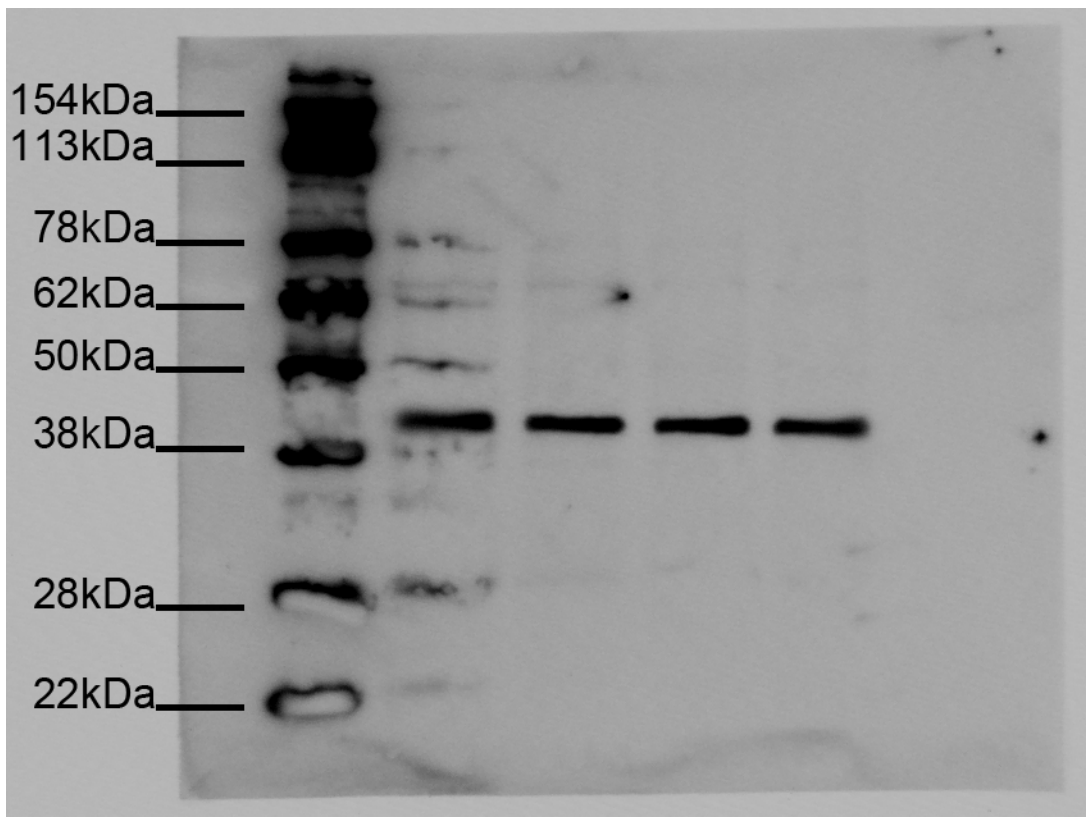

Control、SLQG-5%、SLQG-15%、SB431542

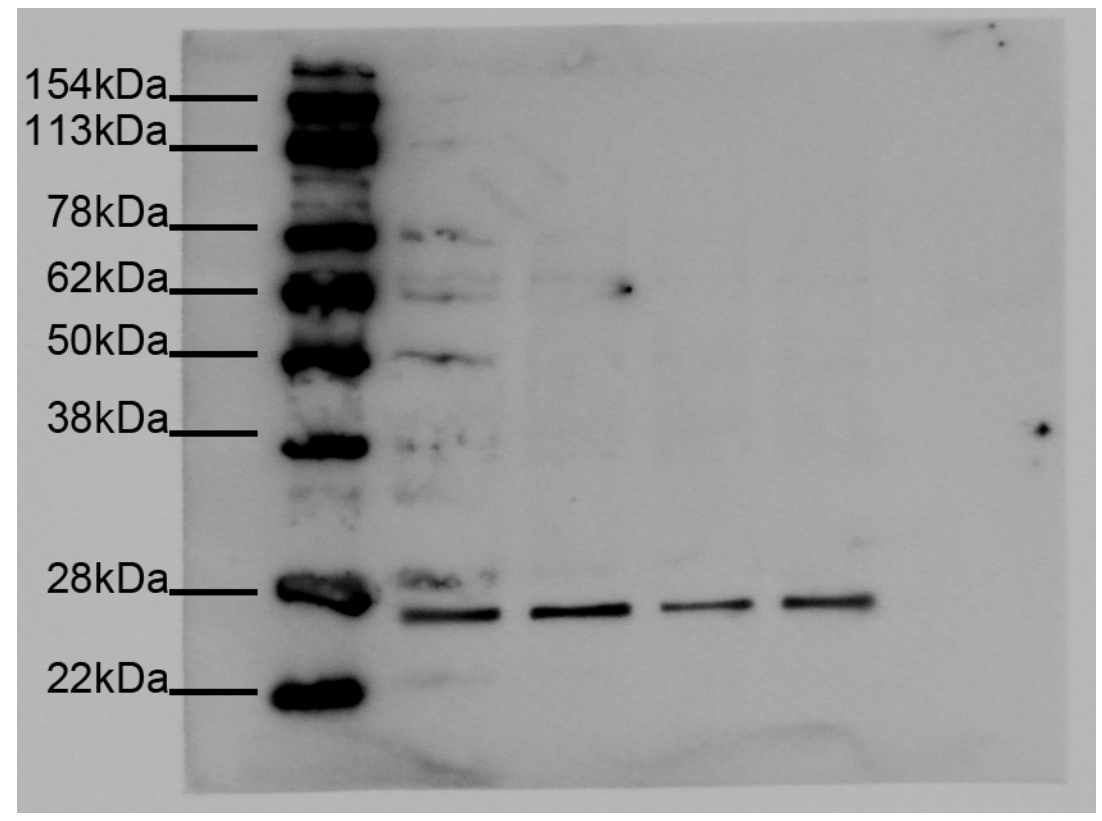

BCL2-2

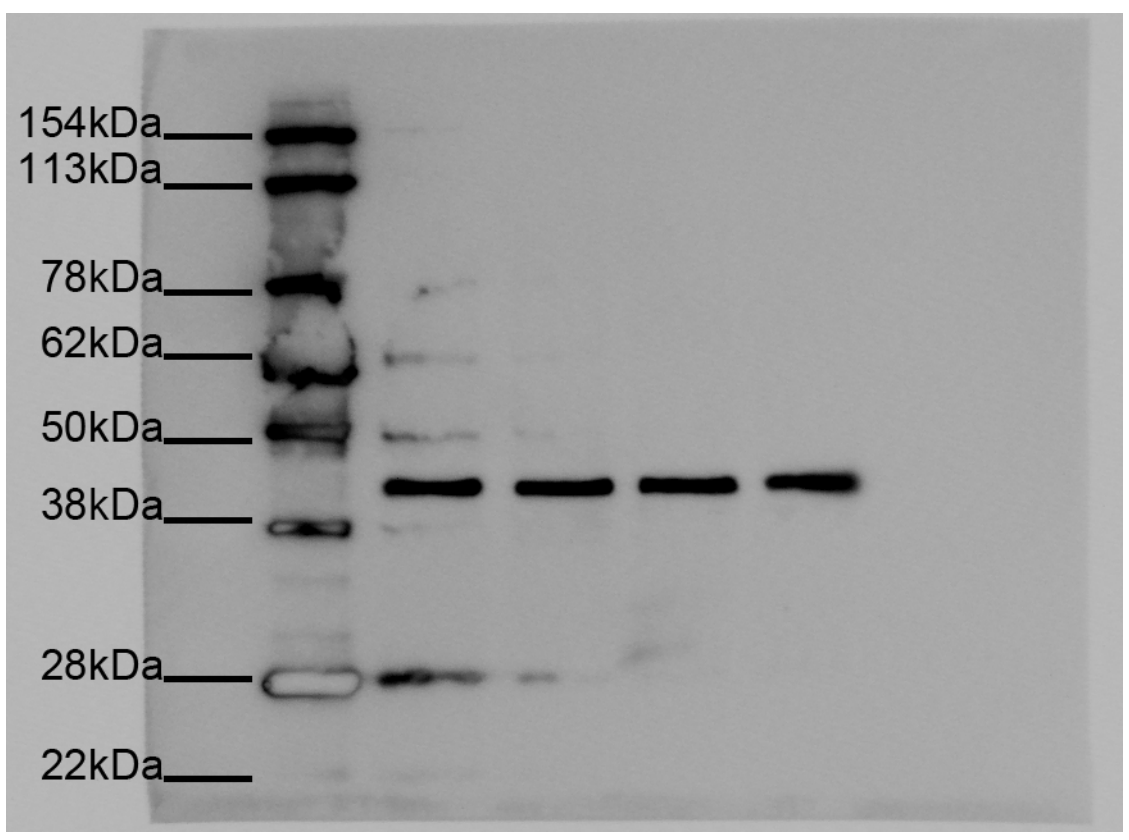

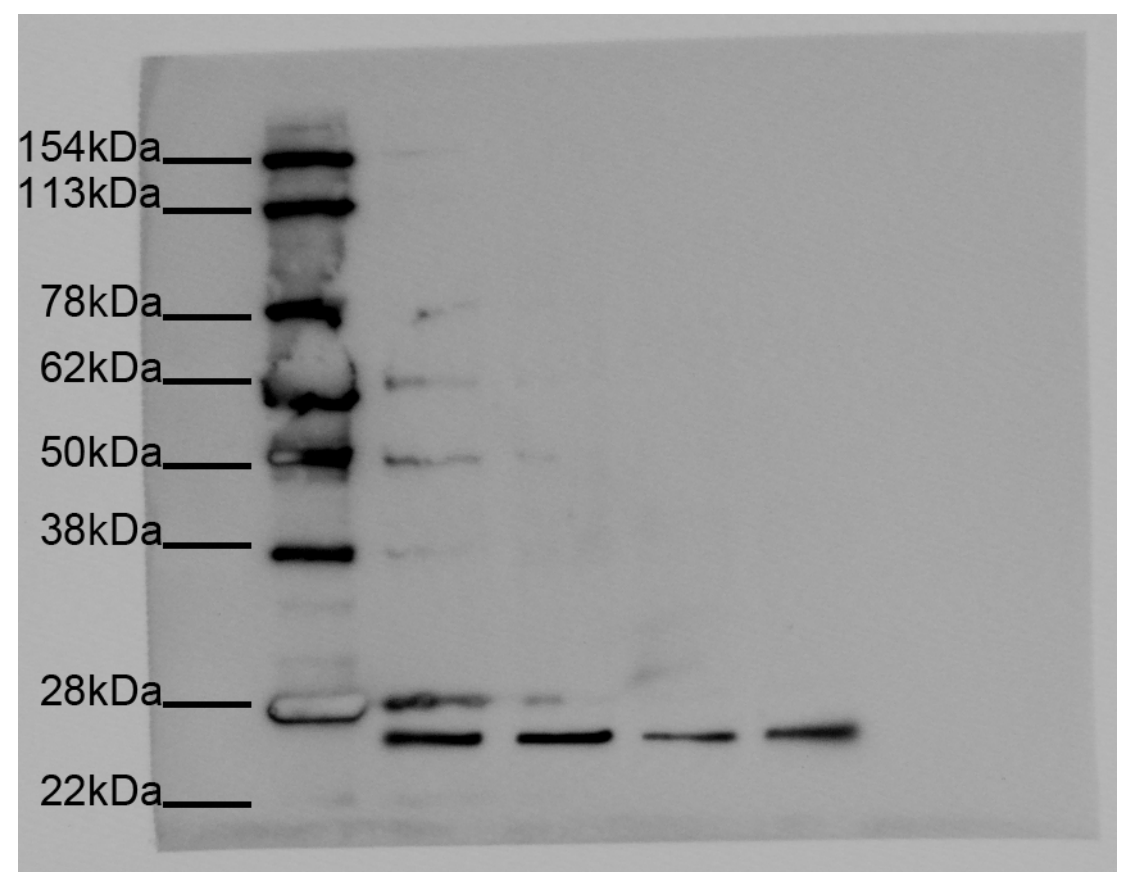

BCL2-3

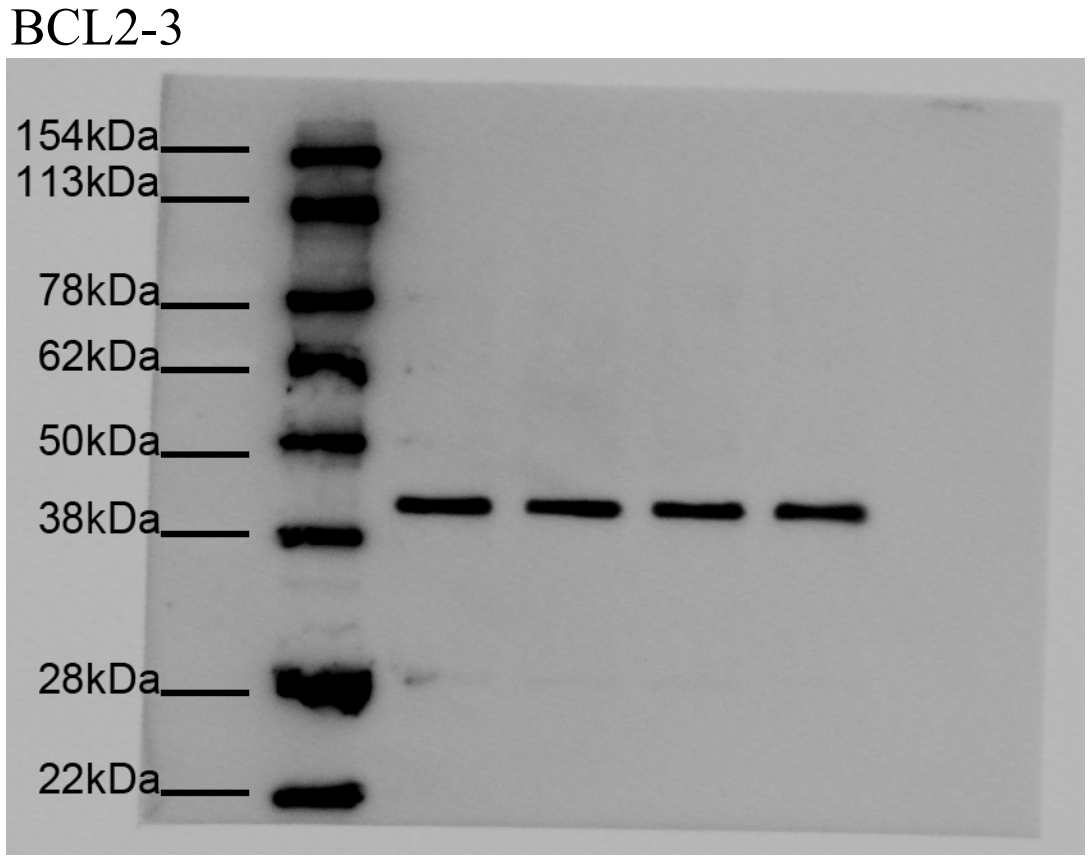

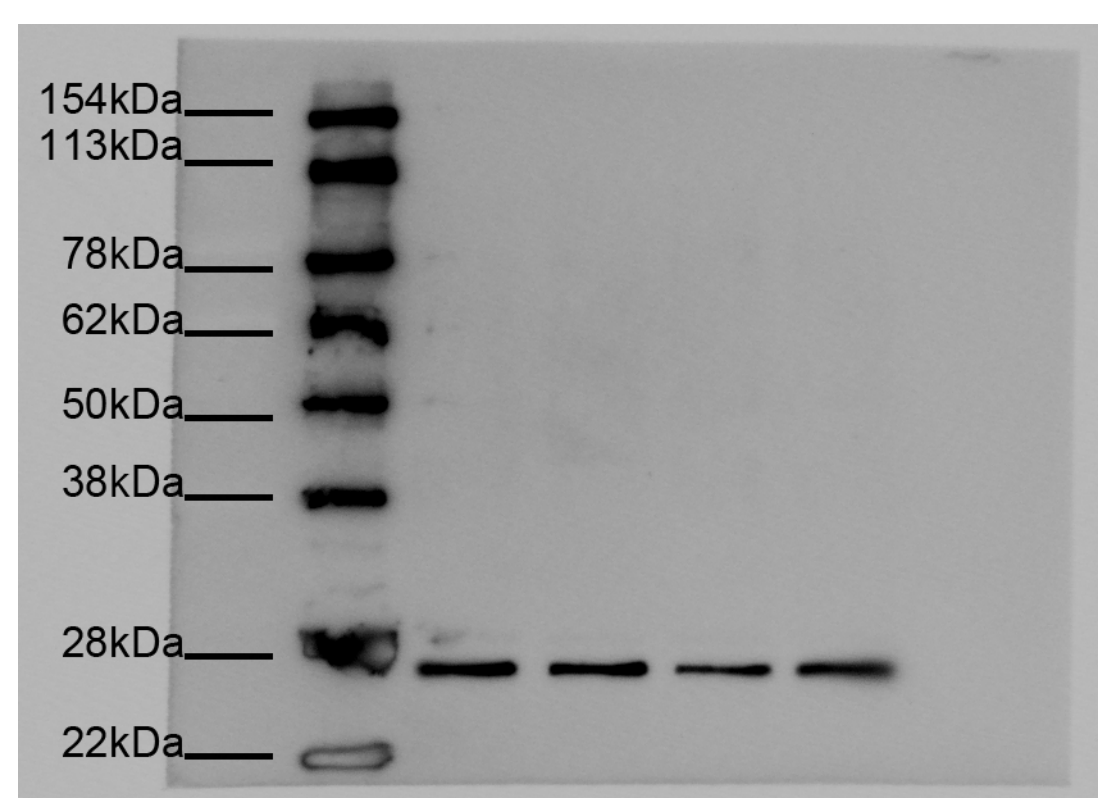

Fig. 7A–H

Cyclin D1-1

Control、SLQG-5%、SLQG-15%、SB431542

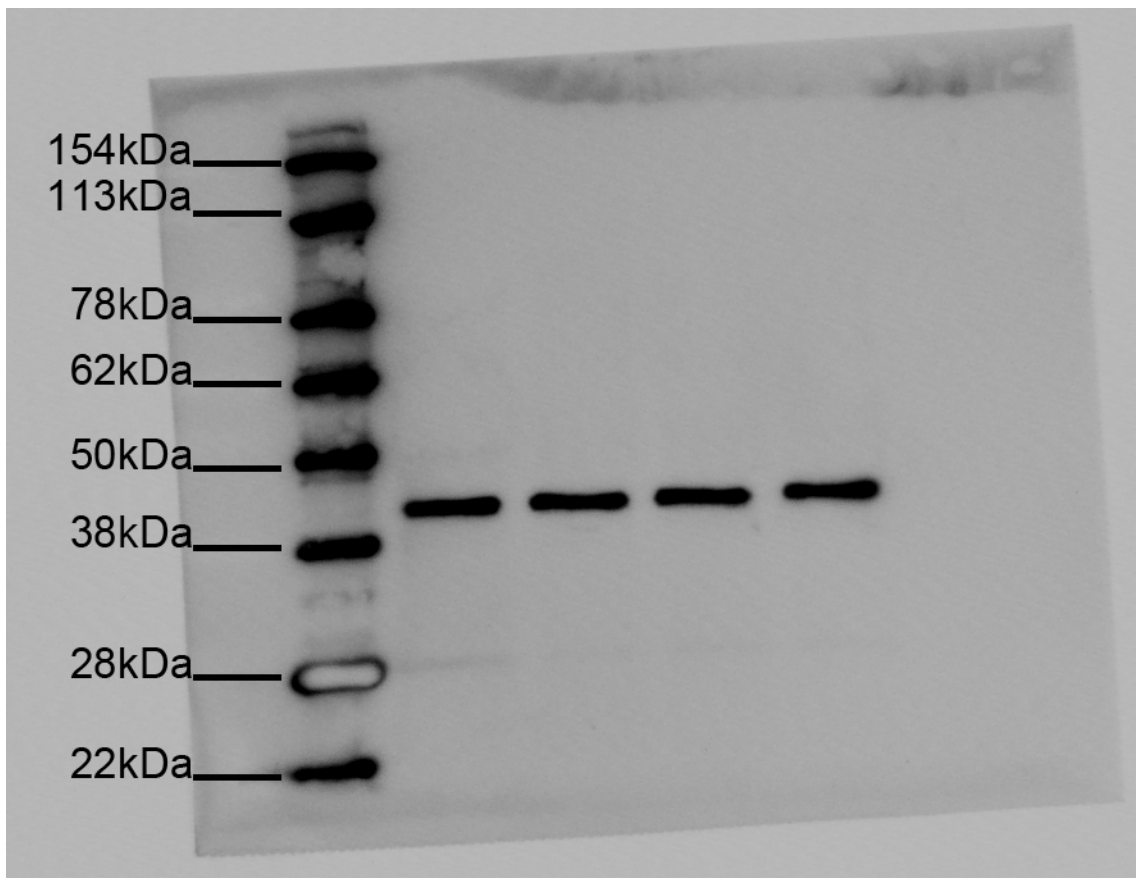

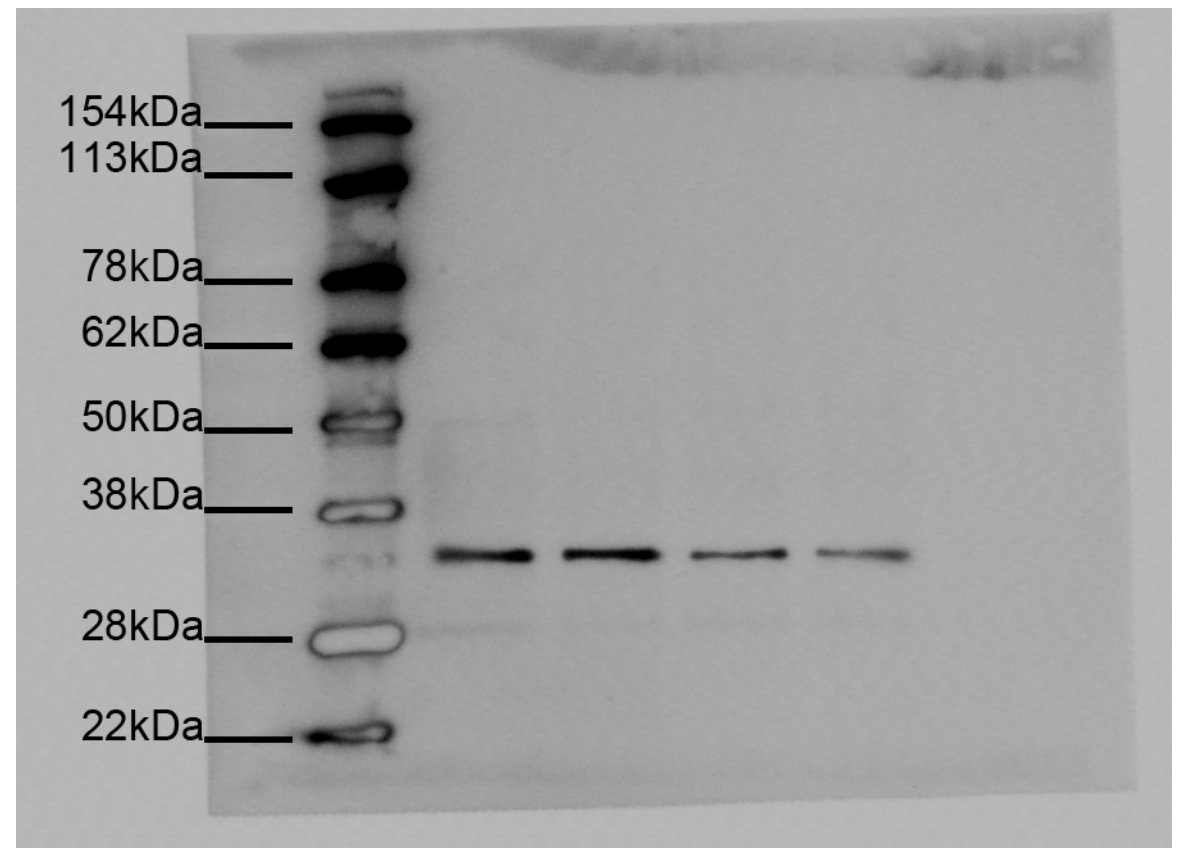

Cyclin D1-2

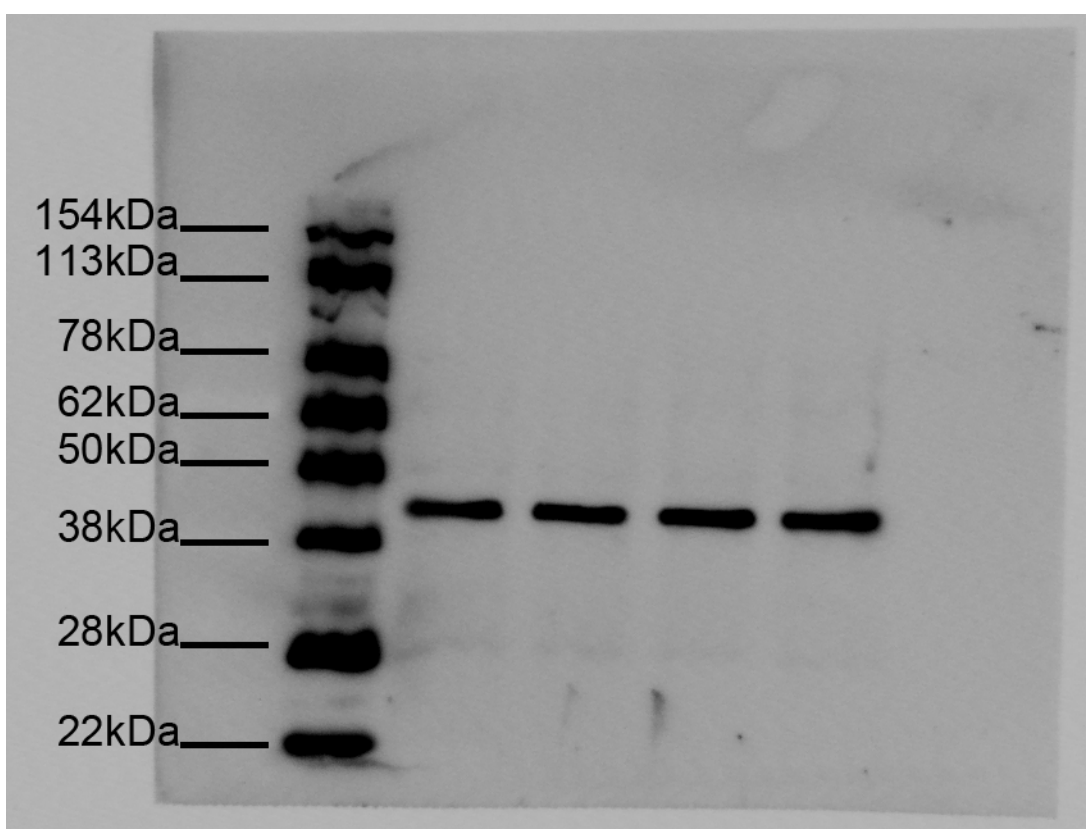

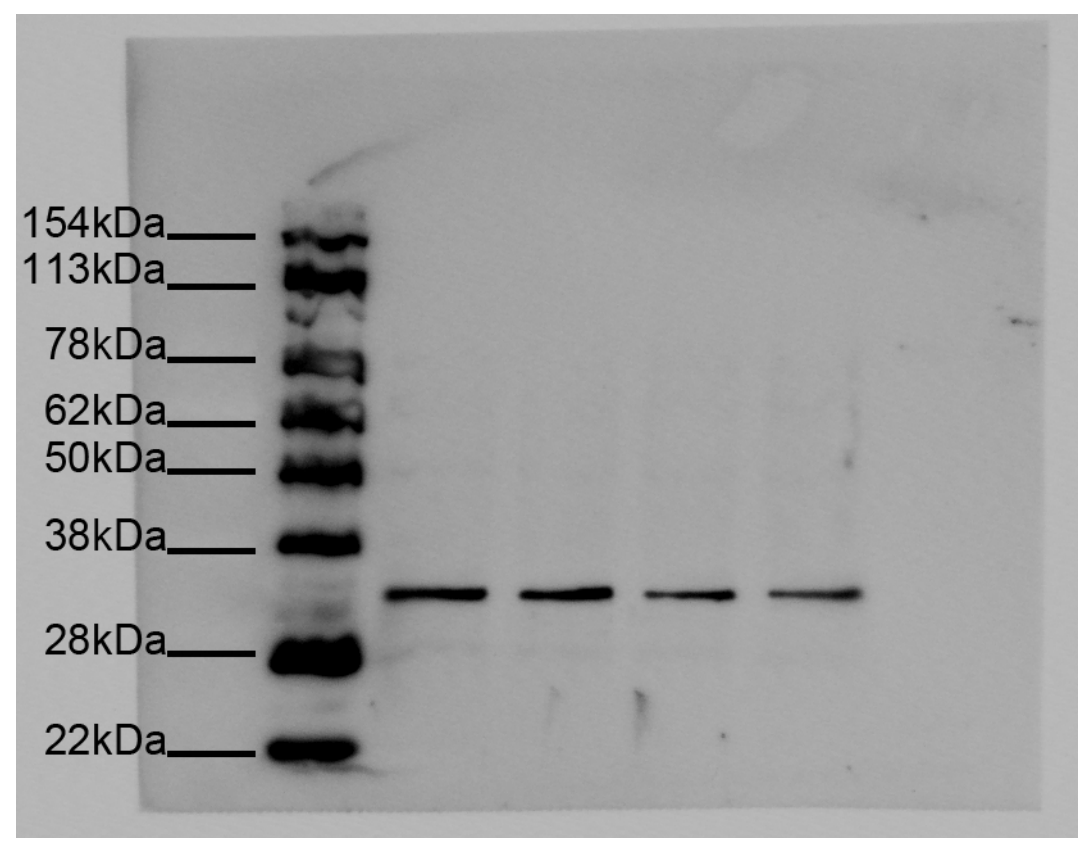

Cyclin D1-3

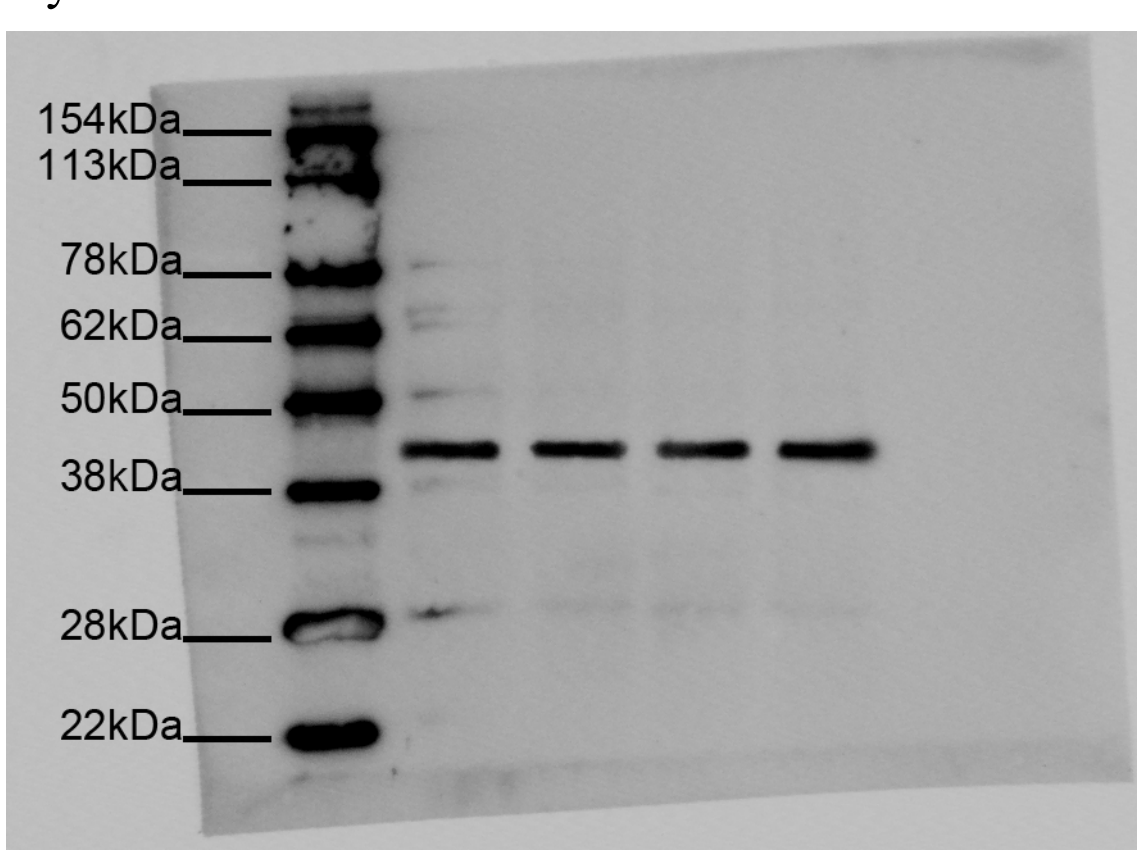

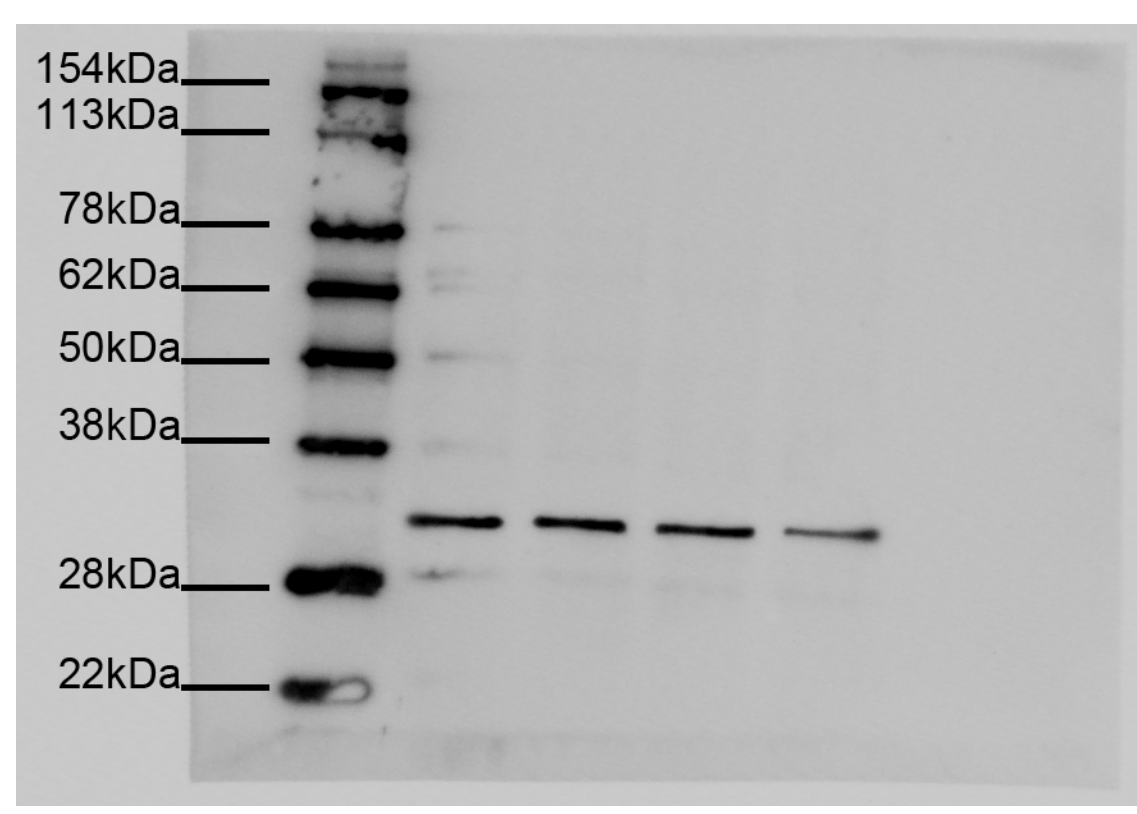

Fig. 7A–H

p21-1

Control、SLQG-5%、SLQG-15%、SB431542

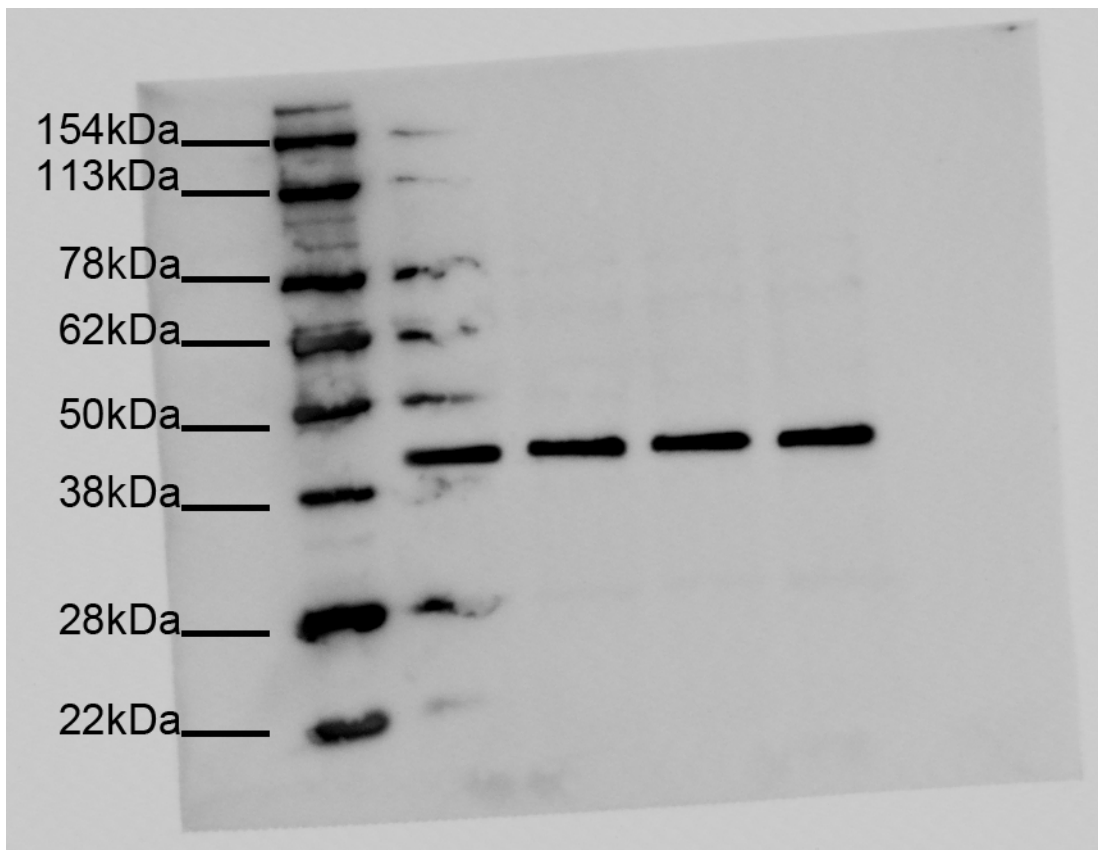

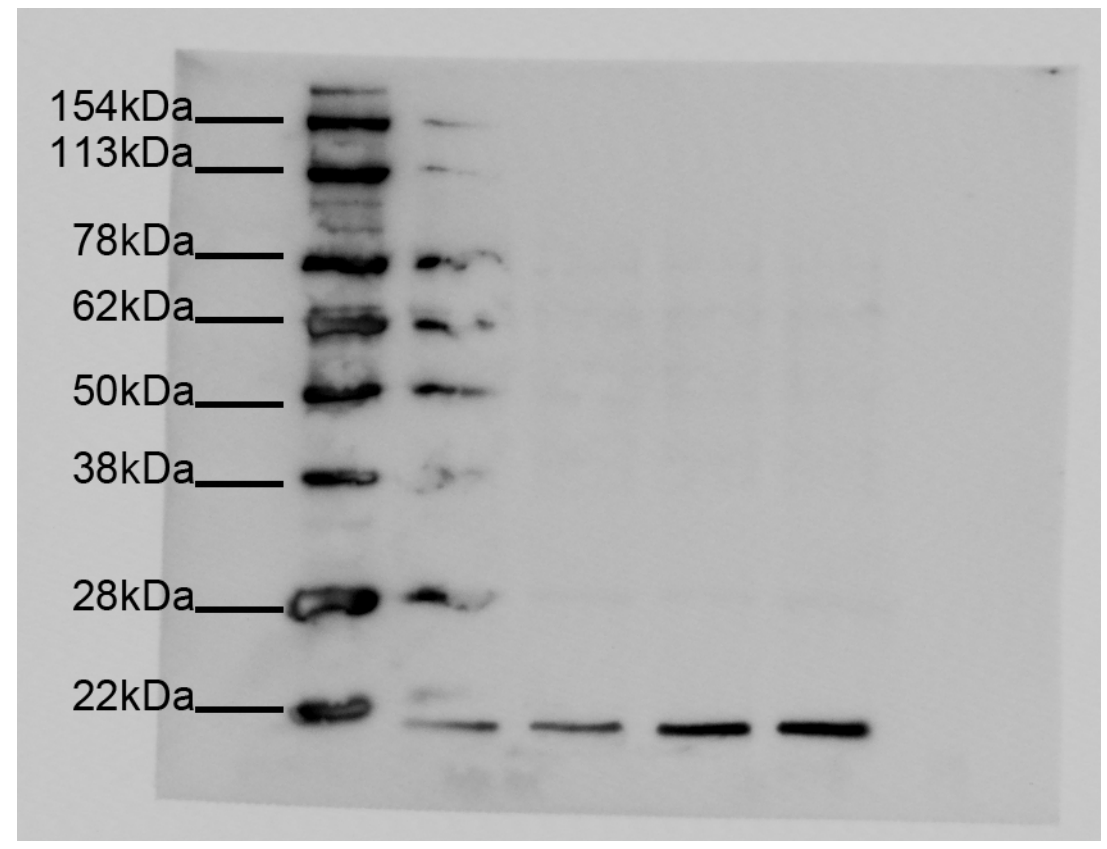

p21-2

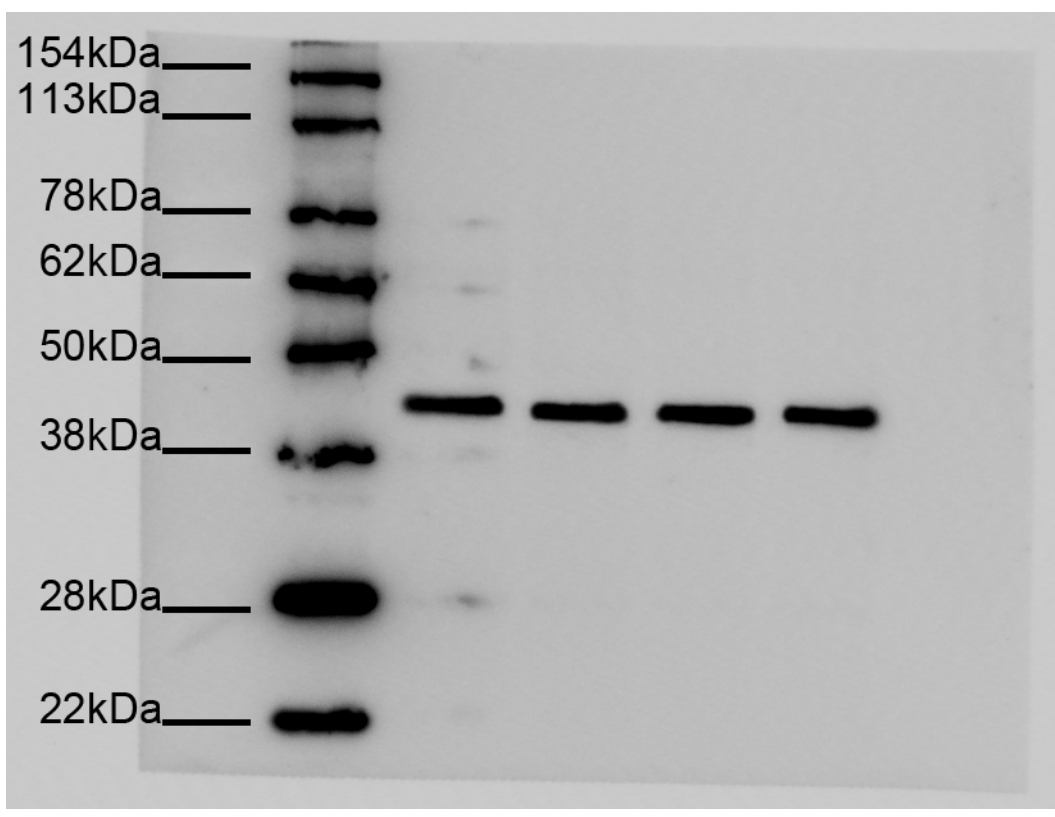

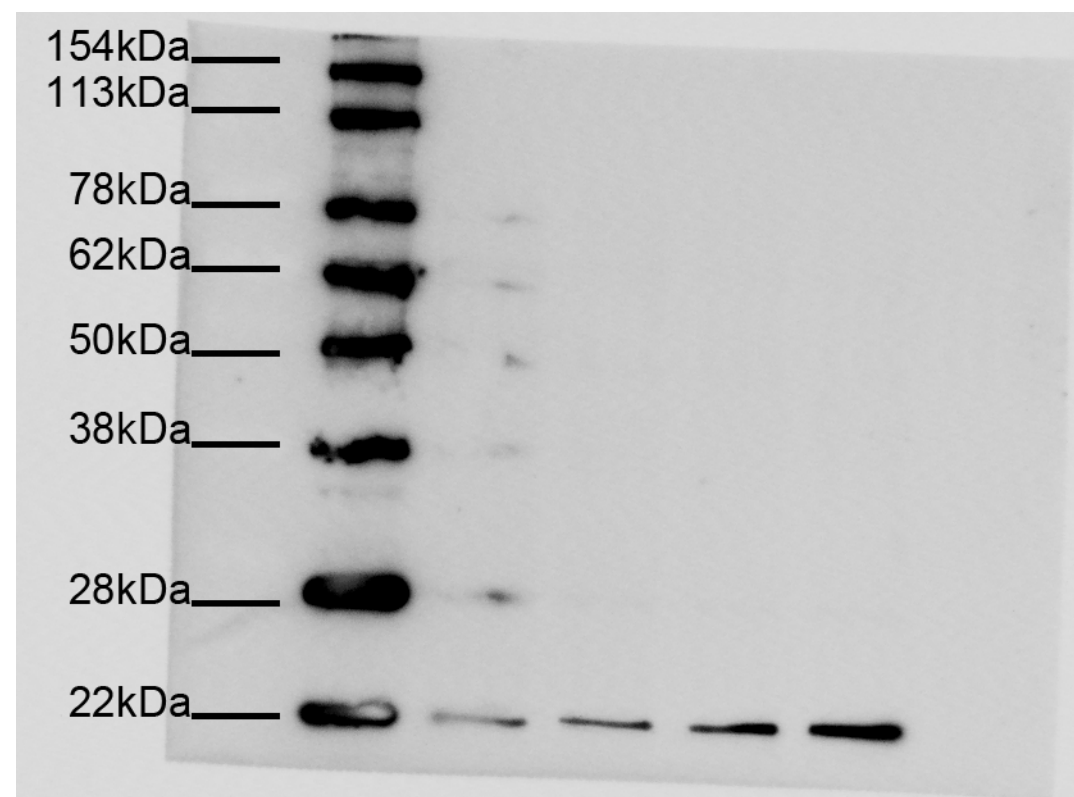

p21-3

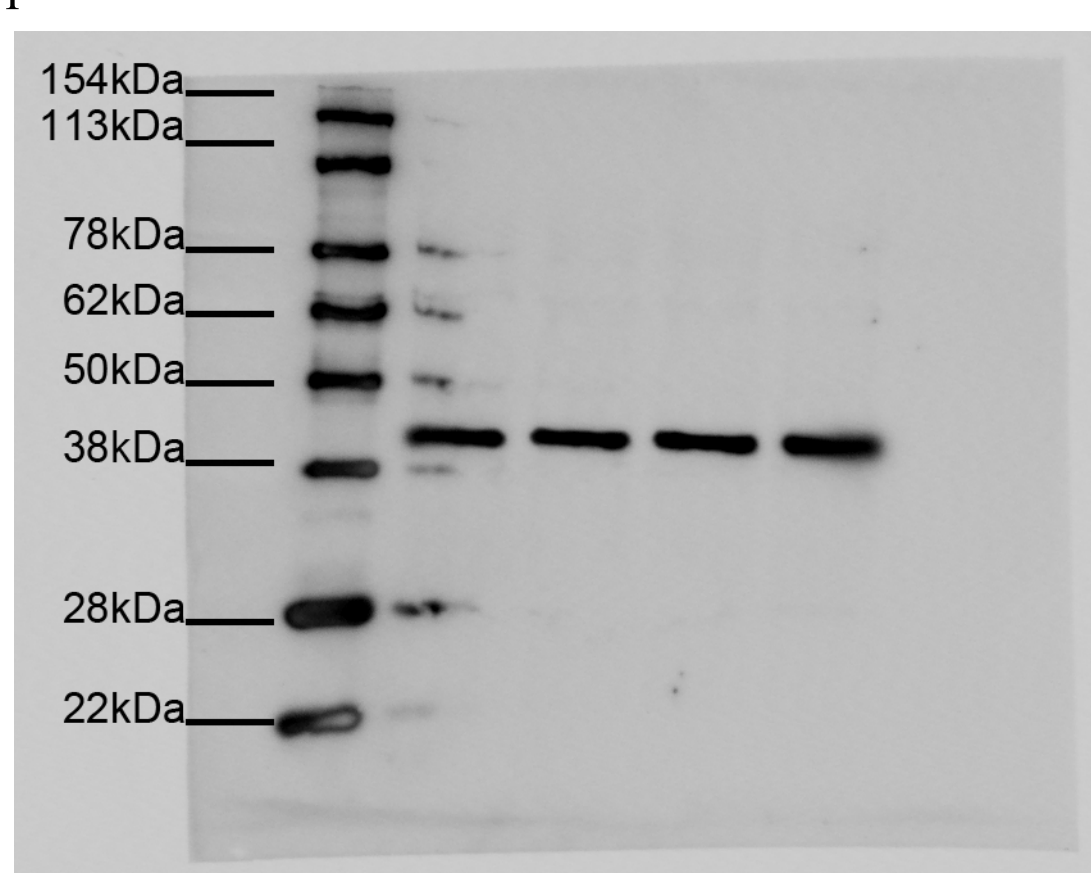

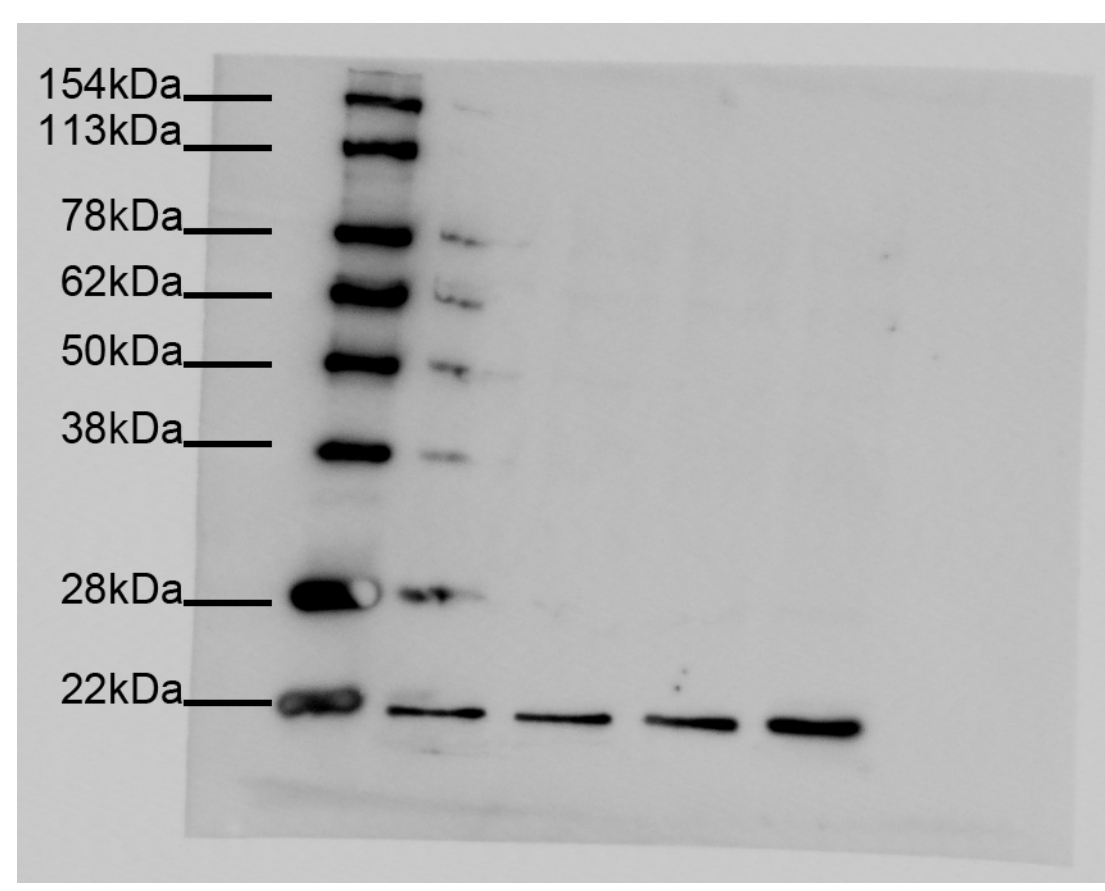

Fig. 7J–O

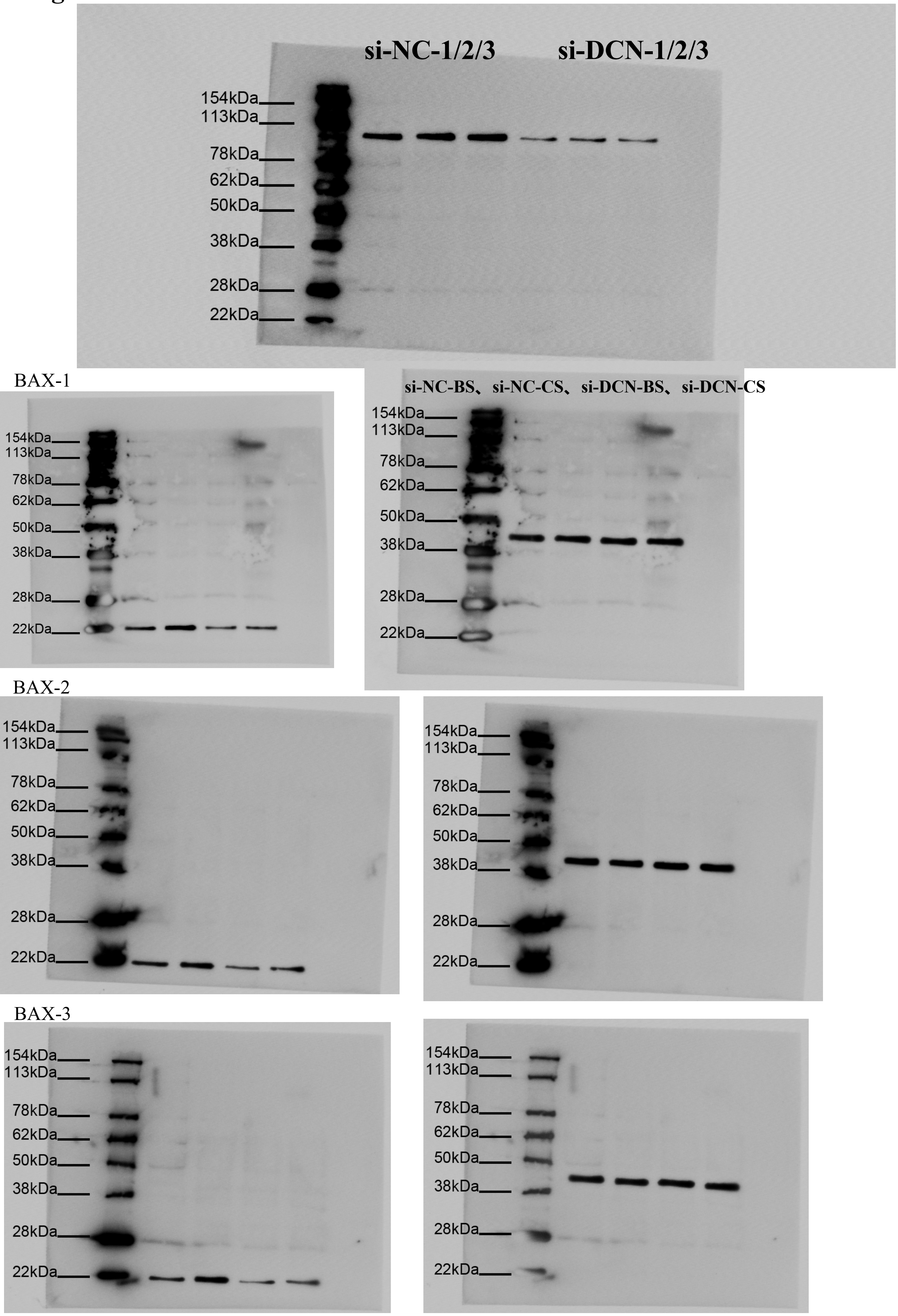
si-NC-1/2/3
si-DCN-1/2/3
154kDa
113kDa
78kDa
62kDa
50kDa
38kDa
28kDa
22kDa
BAX-1
si-NC-BS、si-NC-CS、si-DCN-BS、si-DCN-CS
BAX-2
BAX-3

Fig. 7J–O

p-Smad2/3-1

si-NC-BS、 si-NC-CS、 si-DCN-BS、 si-DCN-CS

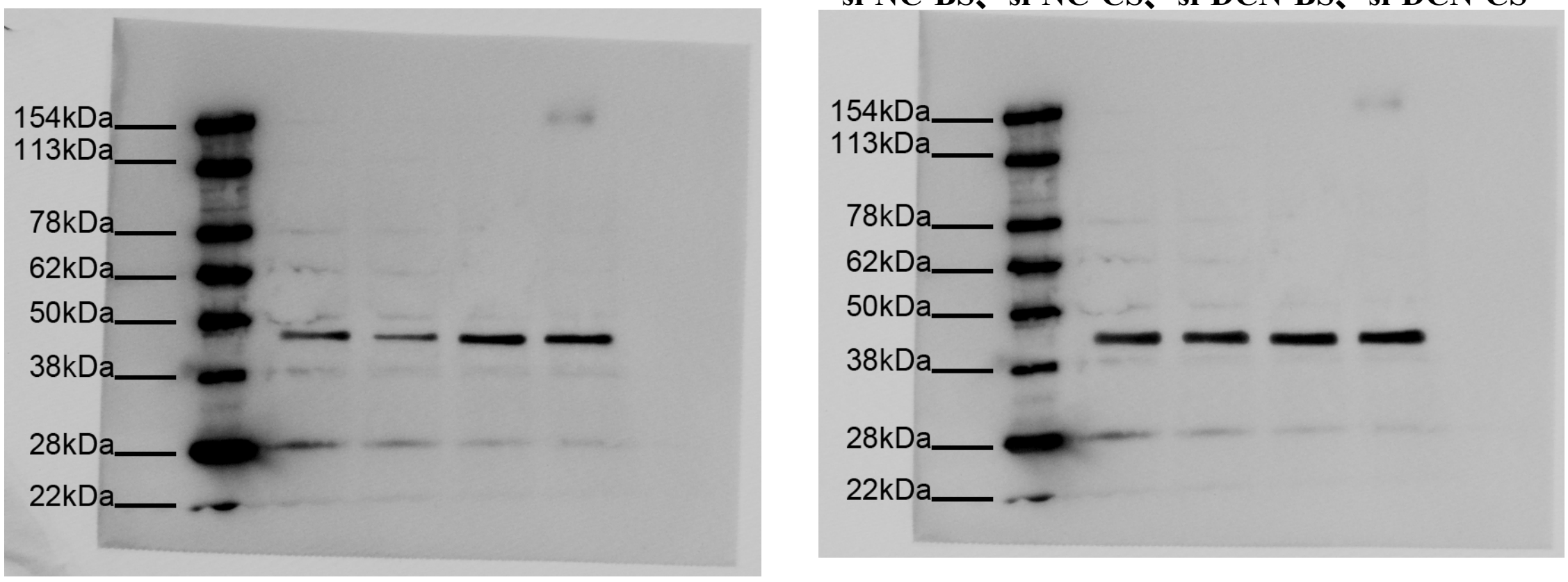

p-Smad2/3-2

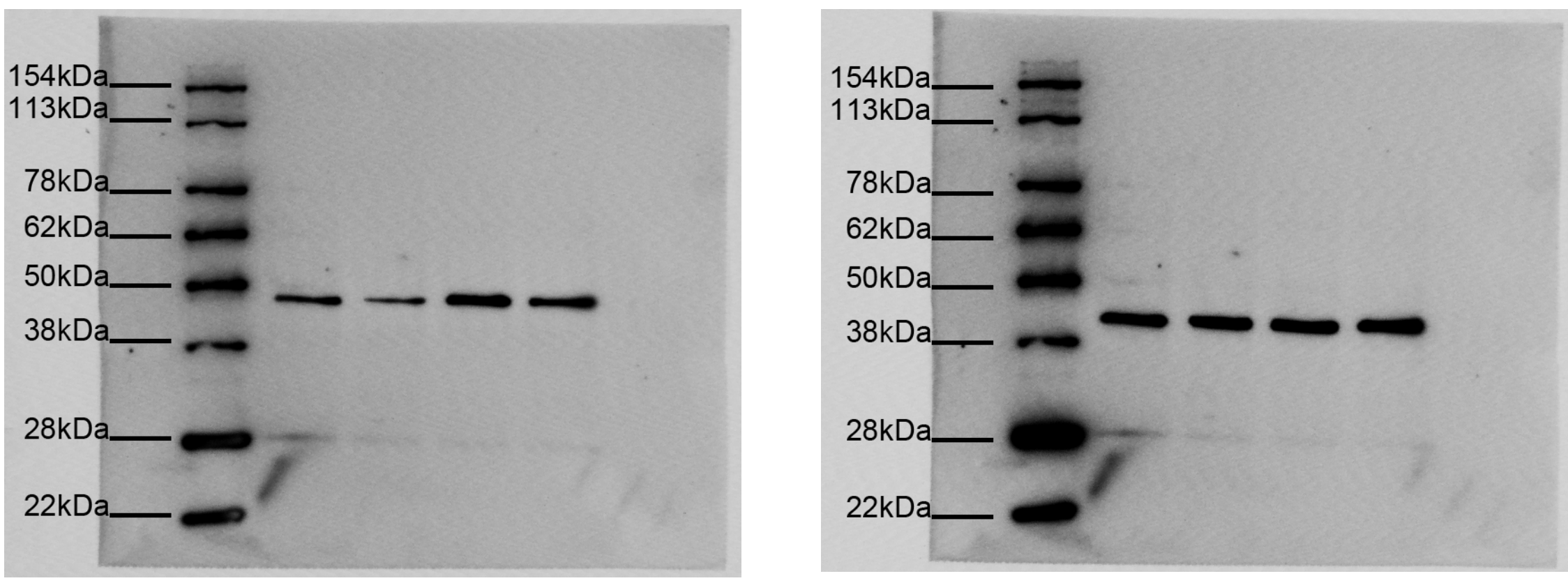

p-Smad2/3-3

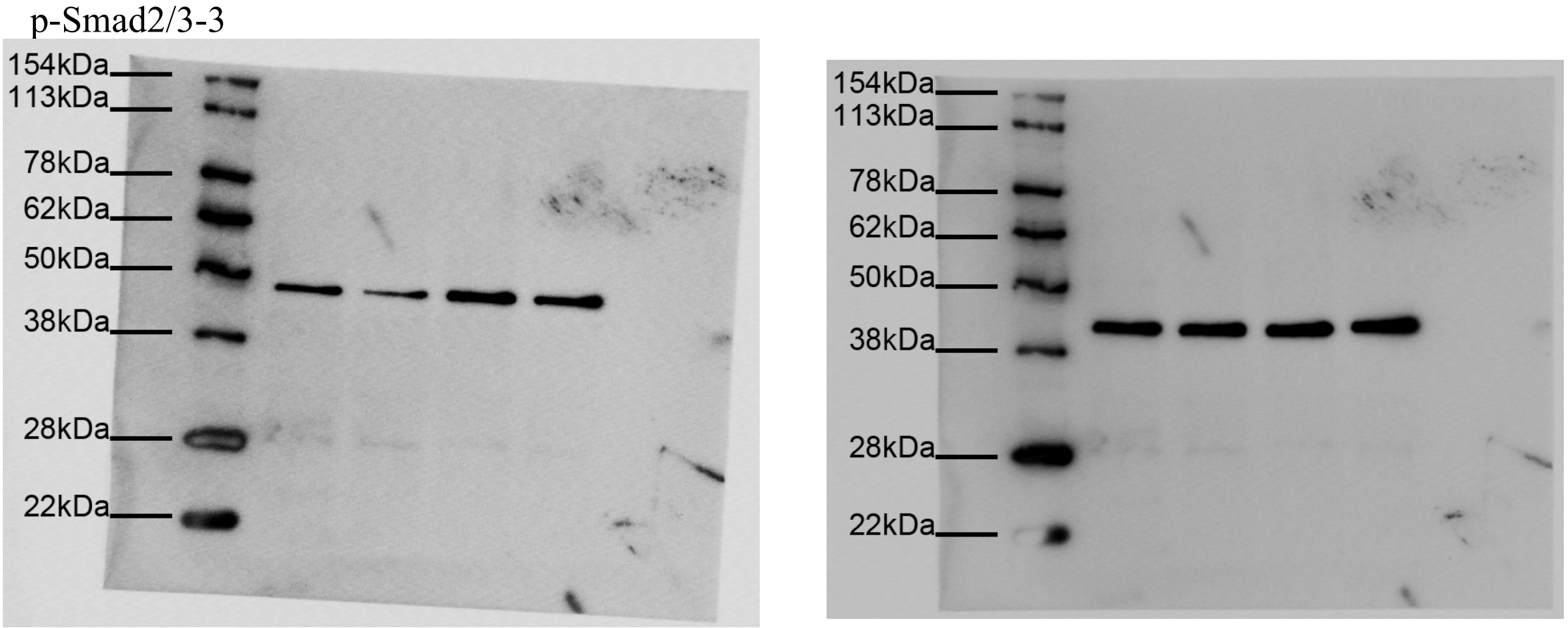

Fig. 7J–O

Cyclin D1-1

si-NC-BS、si-NC-CS、si-DCN-BS、si-DCN-CS

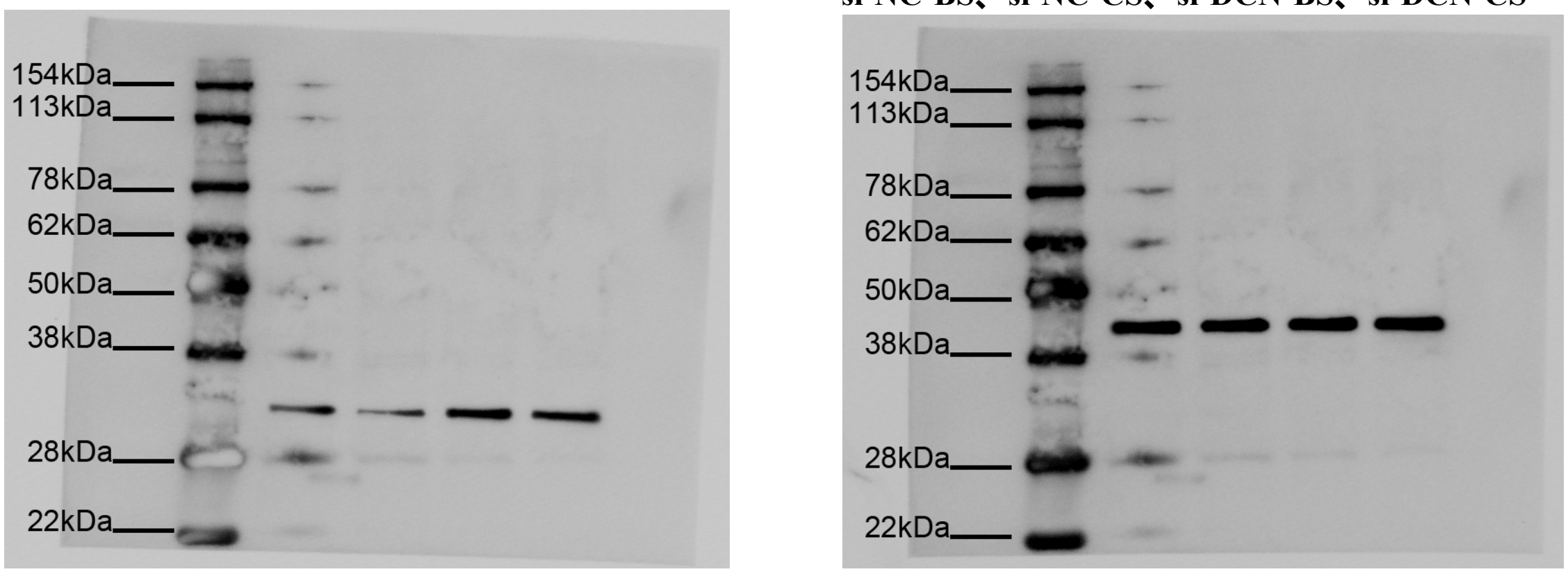

Cyclin D1-2

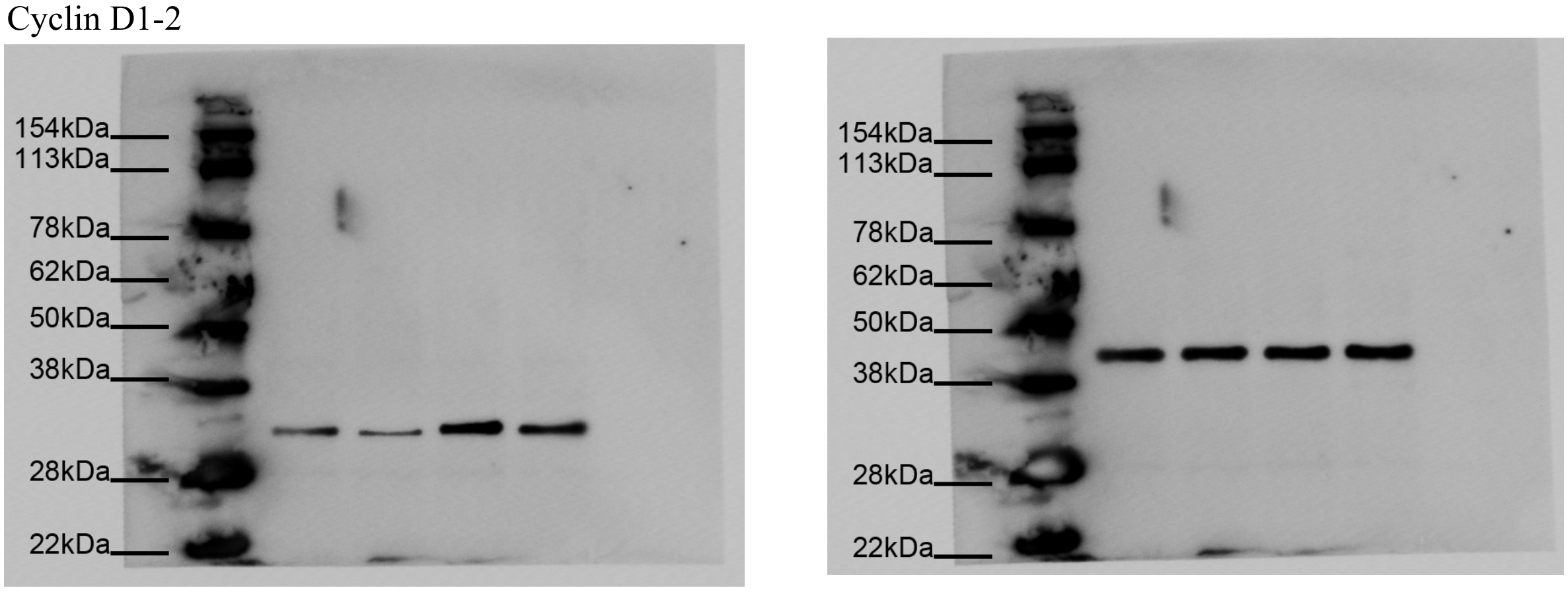

Cyclin D1-3

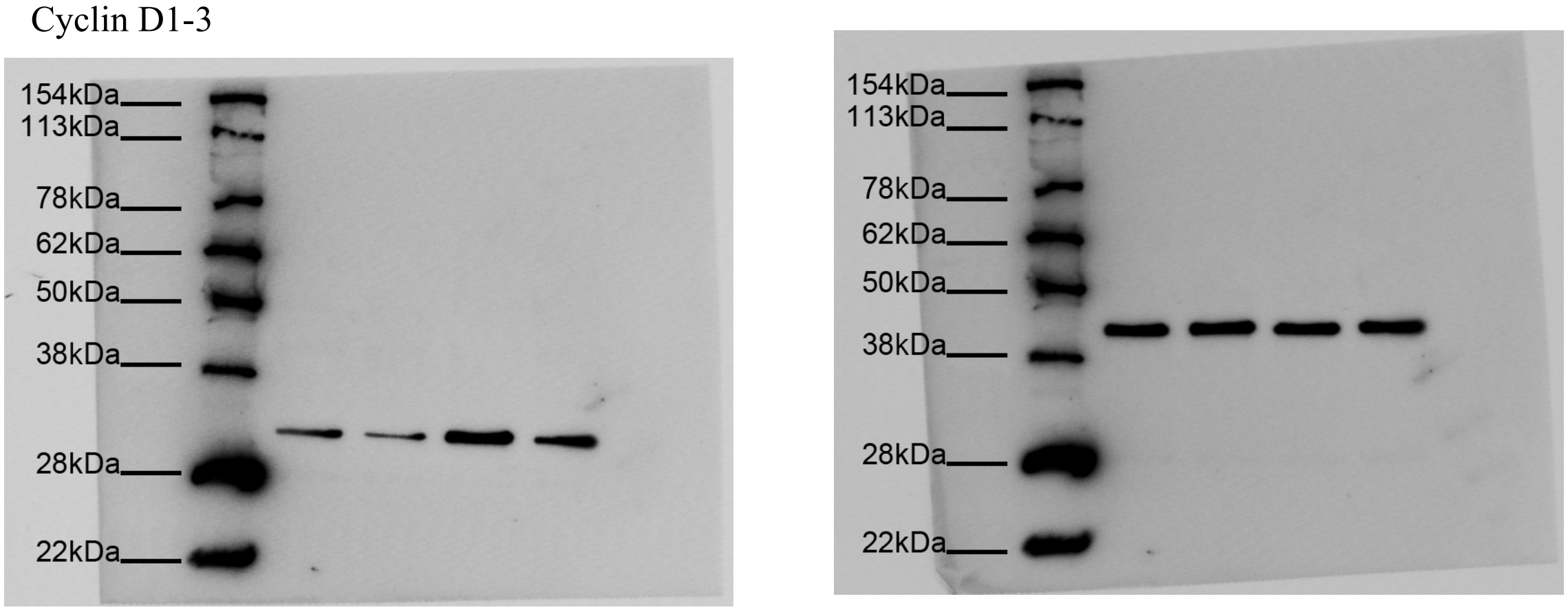

Supplement: Supplementary file 1 [file pone.0359405.s002.pdf]
