# Supplementary material for: Shenling Qige Granules inhibit esophageal squamous cell carcinoma progression in TE-1 xenograft and cell-based models by modulating the DCN–TGF-β/Smad signaling axis
Source: PLoS One. 2026 Oct 6;21(10):e0359405. doi: 10.1371/journal.pone.0359405 (PMC13641691; doi:10.1371/journal.pone.0359405)
Supplement: Supplementary file 2 [file pone.0359405.s001.docx]

**Supporting information**

S1 Fig

A

B

C

D

E

F

G

**S1 Fig** Representative docking conformations of DCN with the remaining seven major active compounds of SLQG. (A) Quercetin–DCN. (B) Luteolin–DCN. (C) α-Limonene–DCN. (D) D-Limonene–DCN. (E) β-Pinene–DCN. (F) Elemicin–DCN. (G) Tanshinone IIA–DCN. The three compounds with docking energies below –7.0 kcal/mol are shown in Fig. 2B–D.

S2 Fig

A

B

C

**S2 Fig** Solvent-accessible surface area analysis of DCN–ligand complexes during 100-ns molecular dynamics simulations. (A) β-sitosterol–DCN. (B) stigmasterol–DCN. (C) ursolic acid–DCN.

S1 Table. Constituents previously identified in Shenling Qige Granules by UHPLC-Q-Orbitrap HRMS

| No. | Molecular formula | Tentatively identified constituent | Source medicinal material(s) |
| --- | --- | --- | --- |
| 1 | C_6_H_14_N_2_O_2_ | L-Lysine | Scorpio |
| 2 | C_9_H_11_NO_3_ | L-Tyrosine | Scorpio |
| 3 | C_10_H_13_N_5_O_4_ | Adenosine | Glehniae Radix; Poria; Amomi Fructus; Curcumae Radix |
| 4 | C_10_H_13_N_5_O_5_ | Guanosine | Glehniae Radix; Poria; Amomi Fructus |
| 5 | C_9_H_11_NO_2_ | L-Phenylalanine | Glehniae Radix |
| 6 | C_11_H_12_N_2_O_2_ | L-Tryptophan | Glehniae Radix; Scorpio |
| 7 | C_16_H_18_O_9_ | Neochlorogenic acid | Glehniae Radix |
| 8 | C_36_H_30_O_16_ | Unidentified constituent | Salviae Miltiorrhizae Radix et Rhizoma |
| 9 | C_7_H_6_O_3_ | Protocatechualdehyde | Poria; Salviae Miltiorrhizae Radix et Rhizoma |
| 10 | C_16_H_18_O_9_ | Chlorogenic acid | Glehniae Radix; Curcumae Radix; Salviae Miltiorrhizae Radix et Rhizoma; Rabdosiae Rubescentis Herba; Nelumbinis Folium |
| 11 | C_16_H_18_O_9_ | Cryptochlorogenic acid | Glehniae Radix |
| 12 | C_9_H_8_O_4_ | Caffeic acid | Glehniae Radix; Amomi Fructus; Curcumae Radix; Salviae Miltiorrhizae Radix et Rhizoma; Rabdosiae Rubescentis Herba; Nelumbinis Folium |
| 13 | C_8_H_8_O_4_ | Vanillic acid | Glehniae Radix; Poria; Amomi Fructus; Curcumae Radix |
| 14 | C_8_H_8_O_3_ | Vanillin | Curcumae Radix; Nelumbinis Folium |
| 15 | C_9_H_8_O_3_ | p-Coumaric acid | Curcumae Radix; Eupatorii Herba |
| 16 | C_21_H_20_O_12_ | Hyperoside | Glehniae Radix; Amomi Fructus; Rabdosiae Rubescentis Herba; Nelumbinis Folium |
| 17 | C_31_H_48_O_4_ | Unidentified constituent | Poria |
| 18 | C_10_H_10_O_4_ | Ferulic acid | Glehniae Radix; Curcumae Radix; Salviae Miltiorrhizae Radix et Rhizoma; Rabdosiae Rubescentis Herba |
| 19 | C_21_H_20_O_11_ | Astragalin | Nelumbinis Folium |
| 20 | C_7_H_6_O_3_ | Salicylic acid | Glehniae Radix; Curcumae Radix; Nelumbinis Folium |
| 21 | C_14_H_18_O_9_ | Unidentified constituent | Glehniae Radix |
| 22 | C_27_H_45_NO_3_ | Peimine | Fritillariae Thunbergii Bulbus |
| 23 | C_21_H_20_O_11_ | Quercitrin | Amomi Fructus; Nelumbinis Folium |
| 24 | C_27_H_41_NO_3_ | Peimisine | Fritillariae Thunbergii Bulbus |
| 25 | C_18_H_16_O_8_ | Rosmarinic acid | Salviae Miltiorrhizae Radix et Rhizoma; Pogostemonis Herba; Rabdosiae Rubescentis Herba |
| 26 | C_9_H_6_O_2_ | Coumarin | Glehniae Radix; Eupatorii Herba |
| 27 | C_27_H_22_O_12_ | Lithospermic acid | Salviae Miltiorrhizae Radix et Rhizoma |
| 28 | C_27_H_43_NO_3_ | Peiminine | Fritillariae Thunbergii Bulbus |
| 29 | C_19_H_21_NO_2_ | Nuciferine | Nelumbinis Folium |
| 30 | C_36_H_30_O_16_ | Salvianolic acid B | Salviae Miltiorrhizae Radix et Rhizoma |
| 31 | C_16_H_12_O_7_ | Unidentified constituent | Rabdosiae Rubescentis Herba |
| 32 | C_20_H_28_O_6_ | Oridonin | Rabdosiae Rubescentis Herba |
| 33 | C_15_H_10_O_7_ | Quercetin | Glehniae Radix; Amomi Fructus; Nelumbinis Folium |
| 34 | C_31_H_46_O_4_ | Unidentified constituent | Poria |
| 35 | C_27_H_45_NO_2_ | Unidentified constituent | Fritillariae Thunbergii Bulbus |
| 36 | C_12_H_8_O_4_ | Methoxsalen | Glehniae Radix; Salviae Miltiorrhizae Radix et Rhizoma |
| 37 | C_12_H_8_O_4_ | Bergapten | Glehniae Radix |
| 38 | C_15_H_22_O_2_ | Curcumenol | Curcumae Radix; Rabdosiae Rubescentis Herba |
| 39 | C_30_H_44_O_5_ | Poricoic acid B | Poria |
| 40 | C_18_H_12_O_3_ | Tanshinone I | Salviae Miltiorrhizae Radix et Rhizoma |
| 41 | C_19_H_20_O_3_ | Cryptotanshinone | Salviae Miltiorrhizae Radix et Rhizoma |
| 42 | C_27_H_41_NO_4_ | Unidentified constituent | Fritillariae Thunbergii Bulbus |
| 43 | C_31_H_46_O_5_ | Poricoic acid A | Poria; Rabdosiae Rubescentis Herba |
| 44 | C_27_H_45_NO_3_ | Unidentified constituent | Fritillariae Thunbergii Bulbus |
| 45 | C_31_H_48_O_4_ | Dehydrotumulosic acid | Poria |
| 46 | C_19_H_18_O_3_ | Tanshinone IIA | Salviae Miltiorrhizae Radix et Rhizoma |

Note: The constituents listed in S1 Table were previously identified by UHPLC-Q-Orbitrap HRMS in our earlier chemical characterization study of the same prescription, then reported under the name Huazhuo Runzao Jiangqi Formula [9]. They are summarized here as the phytochemical basis for the present DCN-centered mechanistic investigation.

S2 Table. Primer sequences

| GENE | | Primer sequence (5′-3′) |
| --- | --- | --- |
| BAX | Forward primer | CCCGAGAGGTCTTTTTCCGAG |
|  | Reverse primer | CCAGCCCATGGTTCTGAT |
| BCL2 | Forward primer | CATGCTGATGTCTCTGGAATCT |
|  | Reverse primer | GATGTGATGCCTCTGCGAAG |
| GAPDH | Forward primer | CCATGAGAAGTATGACAACAGCC |
|  | Reverse primer | CCTTCCACGATACCAAAGTTG |

S3 Table. Primer sequences

| GENE | | Primer sequence (5′-3′) |
| --- | --- | --- |
| DCN | Forward primer | GGACCGTTTCAACAGAGAGG |
|  | Reverse primer | GACCACTCGAAGATGGCATT |
| CCND1 | Forward primer | CCGCACGATTTCATTGAACACT |
|  | Reverse primer | CGAAGGTCTGCGCGTGTTT |
| CDKN1A | Forward primer | AGGTGGACCTGGAGACTCTCAG |
|  | Reverse primer | TCCTCTTGGAGAAGATCAGCCG |
| GAPDH | Forward primer | CCATGAGAAGTATGACAACAGCC |
|  | Reverse primer | CCTTCCACGATACCAAAGTTG |

S4 Table. siRNA and primer sequences

| GENE | | Primer sequence (5′-3′) |
| --- | --- | --- |
| DCN | Forward primer | GGACCGTTTCAACAGAGAGG |
|  | Reverse primer | GACCACTCGAAGATGGCATT |
| β-actin | Forward primer | AAGTGTGACGTGGACATCCGC |
|  | Reverse primer | CCGGACTCGTCATACTCCTGCT |
| si-DCN-1 | Sense | CAUUGAUUCUUGUCAACAA |
|  | Antisense | UUGUUGACAAGAAUCAAUG |
| si-DCN-2 | Sense | CCAGAUGAUUGUCAUAGAA |
|  | Antisense | UUCUAUGACAAUCAUCUGG |
| si-DCN-3 | Sense | GAUUCUUGUCAACAAUAAA |
|  | Antisense | UUCAGGCUCAACCAACAGCTT |
| Negative control siRNA (si-NC) | Sense | UGGUUUACAUGUCGACUAA |
|  | Antisense | UUAGUCGACAUGUAAACCA |

S5 Table. Predicted candidate compounds of SLQG identified by network pharmacology

| CAS No. | Compound | Source medicinal material(s) | Degree centrality |
| --- | --- | --- | --- |
| 117-39-5 | Quercetin | Glehniae Radix, Nelumbinis Folium, Pogostemonis Herba | 461 |
| 83-46-5 | β-Sitosterol | Glehniae Radix, Amomi Fructus, Curcumae Radix, Fritillariae Thunbergii Bulbus | 156 |
| [491-70-3](https://commonchemistry.cas.org/detail?cas_rn=491-70-3) | Luteolin | Salviae Miltiorrhizae Radix et Rhizoma, Eupatorii Herba | 116 |
| 138-86-3 | α-Limonene | Rabdosiae Rubescentis Herba | 104 |
| 5989-27-5 | D-Limonene | Rabdosiae Rubescentis Herba | 104 |
| 127-91-3 | β-Pinene | Rabdosiae Rubescentis Herba | 99 |
| 83-48-7 | Stigmasterol | Glehniae Radix, Amomi Fructus, Eupatorii Herba | 96 |
| [487-11-6](https://commonchemistry.cas.org/detail?cas_rn=487-11-6) | Elemicin | Rabdosiae Rubescentis Herba | 62 |
| 77-52-1 | Ursolic acid | Rabdosiae Rubescentis Herba | 47 |
| [568-72-9](https://commonchemistry.cas.org/detail?cas_rn=568-72-9) | Tanshinone IIA | Salviae Miltiorrhizae Radix et Rhizoma | 42 |

S6 Table. Molecular docking energy values of the top 10 candidate compounds with DCN

| Target | Ligand | Binding energy (kcal/mol) |
| --- | --- | --- |
| DCN  (UniProt-ID: P07585) | Quercetin | –5.70 |
|  | β-Sitosterol | –7.69 |
|  | Luteolin | –5.14 |
|  | α-Limonene | –4.73 |
|  | D-Limonene | –4.90 |
|  | β-Pinene | –4.61 |
|  | Stigmasterol | –7.89 |
|  | Elemicin | –3.51 |
|  | Ursolic acid | –8.43 |
|  | Tanshinone IIA | –6.01 |
