# Supplementary material for: Shenling Qige Granules inhibit esophageal squamous cell carcinoma progression in TE-1 xenograft and cell-based models by modulating the DCN–TGF-β/Smad signaling axis
Source: PLoS One. 2026 Oct 6;21(10):e0359405. doi: 10.1371/journal.pone.0359405 (PMC13641691; doi:10.1371/journal.pone.0359405)
Supplement: Supplementary file 3 [file pone.0359405.s005.docx]

**Response to Reviewers**

We sincerely thank the Editor and all reviewers for their valuable comments and constructive suggestions, which have greatly helped us improve the quality of our manuscript. Reviewer comments are presented below in *italicized font*, and our responses are provided beneath each comment in blue font. All modifications in the revised manuscript are highlighted in yellow.

**Reviewer #1**

*Comments 1:
The manuscript reported Shenling Qige Granules inhibited esophageal squamous cell carcinoma progression. The study appeared incomplete without identifying the active components in the granules. The medicinal properties of Ursolic acid has been well documented in the literature. What are the significances of the In vivo study with nude mice? The responses of carcinoma in appropriate animal model could physiologically demonstrate the cellular activity? The present study involves voluminous set of data using equipment. The results did not satisfactorily support its conclusion. An alternative approach to evaluate the pharmaceutical properties of the granules is warranted. A formulation of phytochemicals could show multi-arrays of pharmaceutical properties. To focus on a few of the compounds is far from satisfactory.*

Response 1:
We are grateful to the reviewer for the careful evaluation of our study and for raising important considerations regarding the pharmacological interpretation of SLQG as a complex traditional Chinese medicine formulation. We appreciate the reviewer’s emphasis on the importance of clarifying the relationship between chemical constituents and the overall biological effects of herbal preparations, which has helped us further refine the conceptual framework of our study.

In our previous study, the same prescription (previously named Huazhuo Runzao Jiangqi Formula and currently approved as SLQG) was characterized using UHPLC-Q-Orbitrap HRMS, through which 46 constituents were identified. In the present study, this previous chemical characterization was used as the phytochemical foundation of SLQG, whereas the newly generated transcriptomic, computational, in vivo, in vitro, and DCN knockdown experiments were independently conducted to investigate the formula-level pharmacological mechanisms underlying the anti-ESCC effects of SLQG.

We would like to clarify that the purpose of the present study was not to attribute the therapeutic effects of SLQG to a single compound, such as ursolic acid. Although ursolic acid and other constituents identified from SLQG have been reported to possess antitumor activities, SLQG represents a multi-component and multi-target formulation, and its biological effects are more likely to result from the coordinated regulation of multiple constituents and signaling networks. Therefore, the network pharmacology and molecular docking analyses were used to identify potential candidate compounds and provide mechanistic insights rather than to determine the individual contribution of a specific compound to the overall pharmacological activity of SLQG.

Regarding the significance of the nude mouse xenograft model, we appreciate the reviewer’s consideration of the importance of appropriate in vivo models for pharmacological evaluation. In this study, the xenograft model was designed to complement the cellular findings and to determine whether the observed molecular alterations and phenotypic changes were associated with tumor growth inhibition within an in vivo biological environment.

In response to the reviewer’s concerns regarding the interpretation of multiple datasets, we have carefully revised the manuscript to provide a more balanced explanation and have moderated our conclusions accordingly. We now clarify that our findings support the involvement of DCN-associated signaling regulation, including TGF-β/Smad modulation, in SLQG-mediated ESCC inhibition, while recognizing that the pharmacological effects of SLQG may involve broader multi-component and multi-pathway regulation.

**Reviewer #2**

*Comments:*
*Overview & Summary
This manuscript by Liu and colleagues presents a comprehensive preclinical investigation into the antitumor mechanisms of Shenling Qige Granules (SLQG)—a hospital preparation derived from the traditional Chinese medicine formula Qige San—against esophageal squamous cell carcinoma (ESCC). The authors employ an integrative strategy combining transcriptomic RNA sequencing, network pharmacology, molecular docking, 100-ns molecular dynamics (MD) simulations, GEPIA public database analysis, in vivo TE-1 xenografts in nude mice, and in vitro medicated serum assays paired with siRNA-mediated DCN knockdown. The study concludes that SLQG suppresses ESCC proliferation, induces G₀/G₁ cell-cycle arrest, and promotes apoptosis through the DCN–TGF-β/Smad axis.
The study is well-structured, addresses an important therapeutic need in ESCC, and provides commendable transparency regarding raw data and sequencing accessions. However, several major mechanistic, statistical, and conceptual issues must be addressed before the study can meet the rigorous standards required for publication in PLOS ONE.*

Response:

We sincerely thank the reviewer for the careful evaluation of our manuscript and for providing constructive and insightful comments. We appreciate the reviewer’s recognition of the clinical relevance of exploring novel therapeutic strategies for esophageal squamous cell carcinoma and the value of our integrative experimental approach combining transcriptomic analysis, network pharmacology, computational analyses, and experimental validation. We are also grateful for the reviewer’s positive comments regarding the overall organization of the manuscript and the transparency of our data sharing and sequencing information.

We acknowledge the reviewer’s concerns regarding the mechanistic interpretation, statistical rigor, and conceptual framework of the study. These comments are highly valuable and have helped us further improve the clarity, rigor, and scientific interpretation of our manuscript. We have carefully addressed each comment and revised the manuscript accordingly. Detailed responses to each point are provided below.

*Comments 1:
1. Mechanistic Discrepancy in the In Vivo Xenograft Model
In the in vivo TE-1 xenograft assay, treatment with the selective TGF-β receptor inhibitor SB431542 (10 mg/kg i.p.) achieved a non-significant tumor inhibition rate of only 6.93% relative to the Model group (Table 1; P>0.05). Conversely, SLQG-M achieved a statistically significant inhibition rate of 43.43% (P<0.01).
If pharmacologic blockade of TGF-β signaling via SB431542 fails to significantly attenuate TE-1 tumor growth in vivo, claiming that SLQG inhibits ESCC primarily through the TGF-β/Smad axis is not fully supported by the empirical animal data. This discrepancy raises several questions:
Is SB431542 adequately bioavailable at the administered dose and route?
Does the TE-1 xenograft model rely on TGF-β signaling for growth, or are other pathways (EGFR, MET, IGF-1R) more dominant?
Does SLQG engage parallel pathways that are also regulated by Decorin?
Recommendation: The authors must discuss this discrepancy in depth in the Discussion section. Potential explanations to consider:
SB431542 may have poor pharmacokinetic properties in vivo (e.g., rapid clearance, protein binding)
The dose of SB431542 (10 mg/kg) may be insufficient; consider citing literature on effective doses in similar models
SLQG may act through multiple pathways, with TGF-β/Smad being one component rather than the sole mediator
DCN's anti-tumor effects may involve receptor tyrosine kinase inhibition (EGFR, IGF-1R, MET) in addition to TGF-β neutralization
The manuscript's assertion regarding the centrality of the TGF-β/Smad axis in vivo should be appropriately nuanced.*

Response 1:
We sincerely appreciate the reviewer’s insightful comment regarding the discrepancy between the antitumor efficacy of SLQG and SB431542 in the xenograft model. The reviewer has highlighted an important mechanistic consideration, and we fully agree that the differential responses between direct pharmacological blockade of TGF-β signaling and SLQG treatment require careful interpretation within the context of a complex biological system.

In response to this valuable suggestion, we have revised the Discussion section to provide a more balanced interpretation of the role of the DCN–TGF-β/Smad axis in SLQG-mediated antitumor activity. We now clarify that although inhibition of TGF-β receptor signaling alone did not fully recapitulate the tumor growth suppression observed with SLQG, this finding does not diminish the potential contribution of TGF-β/Smad modulation to the overall mechanism. Instead, it suggests that the antitumor effects of SLQG may involve broader DCN-associated signaling regulation and coordinated modulation of multiple pathways rather than being solely dependent on TGF-β/Smad inhibition.

Specifically, we have added a paragraph discussing several possible explanations for this discrepancy, including the potential influence of SB431542 pharmacological properties in vivo, the complexity of TE-1 xenograft signaling networks, and the additional regulatory roles of DCN in receptor tyrosine kinase pathways. We have also appropriately moderated our interpretation of the DCN–TGF-β/Smad axis, emphasizing that it represents an important but not exclusive component of the pharmacological mechanism underlying the antitumor effects of SLQG.

We sincerely thank the reviewer again for this constructive suggestion, which has helped us improve the mechanistic depth and scientific rigor of our manuscript.

*Comments 2:
2. Non-Monotonic Dose Response and Potential High-Dose Toxicity
The medium dose (SLQG-M) exhibited superior tumor suppression (43.43%) compared to the high dose (SLQG-H, 23.13%).
Figure 3D indicates that mice in the SLQG-H group experienced progressive body weight loss during the treatment period.
In vitro, 15% SLQG-containing serum showed greater efficacy than 20% serum.
This bell-shaped dose-response curve, paired with weight loss at elevated doses, suggests potential systemic toxicity, target saturation, receptor desensitization, or off-target effects at high concentrations. This is a critical pharmacological concern that challenges the interpretation of the results.
Recommendation:
(1) Provide preliminary organ safety/systemic toxicity metrics:
Serum biochemical parameters (e.g., AST, ALT, BUN, creatinine)
Representative histopathology of major non-target organs (liver, kidney, heart, lung)
Organ weight data (liver/kidney/spleen to body weight ratios)
(2) Offer a formal mechanistic interpretation in the Discussion explaining why the high dose yielded reduced antitumor efficacy relative to the medium dose. Potential explanations to consider:
Saturation of absorption or transport mechanisms
Receptor desensitization or downregulation at high concentrations
Presence of both agonistic and antagonistic components in the extract
High-dose toxicity leading to impaired general health and indirect effects on tumor growth
Hormetic response or U-shaped dose-response curve
(3) Consider whether repeated-dose pharmacokinetic studies in mice (e.g., measuring plasma concentrations of key compounds at different SLQG doses) would help clarify exposure-response relationships.
(4) Acknowledge this as a limitation that warrants future pharmacokinetic and toxicological investigation.*

Response 2:
Thank you for your valuable comments.We sincerely appreciate the reviewer’s insightful comment regarding the non-monotonic dose-response relationship observed in our study. The reviewer has correctly highlighted an important pharmacological consideration, and we fully agree that the observation that the medium-dose group (SLQG-M) exhibited a stronger antitumor response than the high-dose group (SLQG-H) requires careful interpretation. This valuable suggestion has prompted us to further consider the complexity of dose-response relationships in multi-component herbal formulations.

We would like to clarify that the present study was primarily designed to investigate the antitumor efficacy and underlying molecular mechanisms of SLQG in an ESCC xenograft model rather than to perform a dedicated systemic toxicity evaluation. Therefore, based on the current experimental design, we believe that the reduced antitumor efficacy observed in the SLQG-H group cannot be conclusively attributed to systemic toxicity alone. In addition, body-weight reduction was also observed in the Model, DDP, and SB431542 groups, suggesting that changes in body weight may reflect the complex physiological consequences associated with tumor progression and therapeutic intervention rather than being exclusively caused by SLQG administration.

In response to the reviewer’s valuable suggestion, we have expanded the Discussion section to provide a more comprehensive and cautious interpretation of this non-linear dose-response pattern. Specifically, we have discussed that several pharmacological factors, including differences in absorption, bioavailability, exposure-response relationships, target engagement, and potential counteracting effects among multiple constituents within complex herbal formulations, may contribute to the reduced efficacy observed at the higher dose. We have also emphasized that this phenomenon should be interpreted cautiously and have acknowledged that further pharmacokinetic and toxicological investigations are warranted to clarify the relationship between SLQG exposure, biological response, and safety profiles.

We sincerely thank the reviewer again for raising this important point, which has helped us improve the pharmacological interpretation and scientific rigor of our manuscript.

*Comments 3:
3. DCN Pathway Specificity and Mechanistic Depth
The siRNA knockdown experiments demonstrate that DCN silencing attenuates SLQG effects (Fig 7L-O), but several important questions remain:
Are the effects of SLQG mediated primarily through DCN, or is DCN upregulation a secondary consequence of broader pathway modulation? The partial reversal after DCN knockdown (p-Smad2/3 partially restored, BAX and Cyclin D1 changes attenuated) suggests incomplete dependence on DCN.
Does DCN overexpression alone recapitulate the anti-tumor effects of SLQG? A gain-of-function approach would strengthen the mechanistic argument.
Why is the effect only partially reversed by DCN knockdown? This may indicate the involvement of additional pathways (identified in network pharmacology) that contribute to SLQG's overall activity.
Recommendation:
Discuss the "partial reversal" finding more explicitly, acknowledging that SLQG likely acts through multiple targets
Consider performing DCN overexpression experiments as a complementary gain-of-function approach for future studies
Address whether other components of the TGF-β/Smad pathway (e.g., Smad4, Smad7, TGFBR1, TGFBR2) were examined in the transcriptomic data
Discuss the network pharmacology findings in the context of multi-target mechanisms, including other candidate compounds identified (quercetin, luteolin, tanshinone IIA)*

Response 3:
We sincerely appreciate the reviewer’s thoughtful and insightful comments regarding the mechanistic interpretation of DCN in SLQG-mediated antitumor activity. The reviewer has raised an important point that the partial attenuation observed after DCN knockdown may indicate that DCN functions as a key regulatory mediator rather than the sole determinant of the biological effects induced by SLQG. This suggestion has helped us further refine our understanding and interpretation of the multi-component and multi-target characteristics of SLQG.

In response to this valuable suggestion, we have carefully revised the Discussion section to provide a more balanced interpretation of the role of DCN. Specifically, we have clarified that although DCN knockdown attenuated the effects of SLQG on p-Smad2/3, BAX, and Cyclin D1 expression, the incomplete reversal indicates that additional signaling networks and molecular targets may also contribute to the overall antitumor activity of SLQG. This interpretation is consistent with the pharmacological characteristics of traditional Chinese medicine formulations, in which multiple bioactive constituents may collectively regulate complex biological processes.

Furthermore, we have expanded the discussion of our network pharmacology findings and incorporated the potential contributions of additional candidate compounds, including quercetin, luteolin, ursolic acid, and tanshinone IIA. These compounds are now described as potential contributors and mechanistic clues rather than definitive determinants of SLQG activity, thereby avoiding overinterpretation of individual components within the complex formulation.

We are also grateful for the reviewer’s valuable suggestion regarding the DCN overexpression approach. We agree that a gain-of-function strategy would provide additional evidence regarding the sufficiency of DCN in mediating the observed antitumor effects. However, the primary objective of the present study was to elucidate how the multi-component SLQG formulation regulates ESCC progression, and the current loss-of-function experiment was designed to evaluate the functional contribution of DCN within this regulatory process. We have therefore acknowledged DCN overexpression and broader pathway validation as important directions for future investigation.

In addition, we have further clarified the relationship between transcriptomic analysis and TGF-β/Smad pathway validation. Although transcriptomic analysis highlighted enrichment of the TGF-β signaling pathway, our experimental validation focused on DCN-associated regulation of Smad2/3 phosphorylation based on the integrative prioritization strategy combining transcriptomic analysis and functional validation. Other pathway components, including Smad4, inhibitory Smads, and TGF-β receptors, were not identified as primary candidates in the current analysis and were therefore not further experimentally validated. We have now added this limitation and future perspective to the revised Discussion.

We sincerely thank the reviewer again for these constructive suggestions, which have substantially improved the mechanistic depth, scientific rigor, and interpretation of our manuscript.

*Comments 4:
4. Rationale for Molecular Docking Against Decorin (DCN)
Small molecules identified in SLQG were docked against the extracellular proteoglycan Decorin (DCN) (Section 2.3.5).
Experimental data confirm that SLQG upregulates DCN mRNA and protein expression levels. Small-molecule natural products typically upregulate extracellular proteins by targeting upstream nuclear receptors, transcription factors, or epigenetic modifiers, rather than binding directly to the secreted mature protein. The biological plausibility of small molecules directly binding to extracellular matrix proteins such as DCN is limited.
Recommendation:
Clarify the biological hypothesis underlying small molecule–DCN binding. Are candidate compounds hypothesized to act as chemical chaperones, stabilize DCN conformation, or alter DCN's binding affinity for TGF-β?
Without direct biophysical binding validation (e.g., surface plasmon resonance [SPR], microscale thermophoresis [MST], or isothermal titration calorimetry [ITC]), clearly state in the text that DCN docking represents exploratory computational modeling rather than validated target engagement.
Consider whether docking to upstream regulators (e.g., transcription factors, epigenetic modifiers) would be more biologically plausible.*

Response 4:
We greatly appreciate the reviewer’s careful consideration of the biological rationale underlying the molecular docking analysis between SLQG-derived compounds and DCN. The reviewer has raised an important mechanistic issue regarding the interpretation of computational prediction involving extracellular matrix proteins, and we agree that the biological significance of compound–DCN interactions requires cautious and appropriate explanation.

In response to this valuable suggestion, we have carefully revised both the Methods and Discussion sections to clarify the purpose and interpretation of the molecular docking analysis. Specifically, we have emphasized that the docking analysis was performed as an exploratory computational approach to identify potential molecular associations and generate mechanistic hypotheses, rather than to establish direct compound–DCN binding or validated target engagement.

We have also clarified that the present study does not intend to suggest that SLQG-derived compounds directly regulate DCN exclusively through physical binding to the mature extracellular protein. Instead, the docking and molecular dynamics analyses were incorporated as complementary computational evidence within an integrative framework combining transcriptomic analysis, network pharmacology, and experimental validation. The functional validation in the present study primarily demonstrated that SLQG treatment increased DCN expression and that DCN contributed to the downstream regulation of TGF-β/Smad-related signaling.

Furthermore, we have acknowledged that future studies using direct biophysical approaches, such as surface plasmon resonance (SPR), microscale thermophoresis (MST), or isothermal titration calorimetry (ITC), would be valuable for further clarifying potential compound–DCN interactions. We appreciate the reviewer’s suggestion, which has helped us improve the rigor and biological interpretation of the computational analyses presented in our manuscript.

*Comments 5:
5. Single Cell Line Reliance
All cellular mechanisms and xenograft models relied exclusively on the TE-1 cell line.
ESCC exhibits significant histological and molecular heterogeneity. Relying on a single cell line limits the generalizability of the findings and cannot fully recapitulate the histological heterogeneity or organ-specific microenvironment of clinical ESCC.
Recommendation:
Validate key molecular endpoints (e.g., DCN upregulation and p-Smad2/3 suppression) in a second ESCC cell line (e.g., KYSE150, ECA109, KYSE30) to demonstrate generalizability.
If this is not feasible, explicitly acknowledge the single-cell-line limitation in the Discussion, framing the findings as preliminary preclinical evidence requiring validation across a broader panel of ESCC models.
Consider discussing whether future studies using patient-derived organoids or primary ESCC cells would strengthen translational relevance.*

Response 5:
We appreciate the reviewer’s thoughtful comment regarding the limitation of relying on a single ESCC cell line and the importance of model diversity for improving the generalizability of preclinical findings. The reviewer has appropriately highlighted that ESCC exhibits substantial molecular and histological heterogeneity, and that validation across broader experimental models would further strengthen the translational relevance of our findings.

In the present study, TE-1 cells were selected as an established ESCC model and were consistently used for both xenograft experiments and mechanistic investigations, including transcriptomic analysis, pharmacological intervention, and DCN knockdown validation. The use of a consistent cellular and in vivo model system allowed us to systematically investigate the molecular events associated with SLQG-mediated ESCC inhibition. However, we fully acknowledge that findings derived from a single ESCC cell line cannot completely represent the molecular diversity, immune contexture, and tumor microenvironment complexity of clinical ESCC.

Accordingly, we have further refined the limitation statement in the Discussion section to explicitly acknowledge that the current findings should be interpreted as preliminary preclinical evidence requiring validation in additional ESCC models. We have also emphasized that future studies incorporating additional ESCC cell lines with diverse molecular backgrounds, orthotopic or immunocompetent models, and patient-derived organoids would be valuable to further evaluate the generalizability and translational relevance of SLQG-mediated DCN-associated regulation.

We sincerely thank the reviewer for this constructive suggestion, which has helped us more clearly define the scope of our conclusions and improve the discussion of future research directions.

*Comments 6:
6. Statistical Testing and Interpretation Rigor
Standard ANOVA tests were applied, but critical statistical details are missing.
Recommendation:
(1) Explicitly state whether tests for normality (e.g., Shapiro-Wilk) and homogeneity of variances (e.g., Levene's test) were performed prior to parametric testing. If assumptions were violated, describe the corrective measures applied (e.g., data transformation, non-parametric tests).
(2) Clarify the exact post-hoc test used (e.g., Tukey's HSD vs. Dunnett's test) across all figure legends. The Methods section (Section 2.5) states "Tukey's or Dunnett's post hoc test, as appropriate"—specify which test was used for each experiment.
(3) For cell assays involving multiple serum concentrations across time points (e.g., CCK-8 at 5%, 10%, 15%, 20%), ensure appropriate multi-way or repeated-measures ANOVA with multiple comparison corrections (e.g., Bonferroni or Sidak) is applied to prevent Type I error inflation.
(4) Provide a formal sample size justification: While sample sizes are indicated (n=8 for in vivo, n=3 for many molecular assays), no formal power analysis or sample size calculation is provided. Some mechanistic experiments with n=3 may have limited statistical power to detect moderate effect sizes. Consider citing previous studies or standard practice in the field to justify these numbers.
(5) For Western blot quantification, clarify the normalization strategy and whether all samples were run on the same gels.*

Response 6:
We sincerely appreciate the reviewer’s careful evaluation of our statistical analysis and reporting procedures. We agree that clear descriptions of statistical methods, biological replicates, and protein quantification strategies are essential for ensuring the transparency and reproducibility of experimental studies.

Regarding the assumptions of statistical analyses, we have revised the Statistical analysis section to clarify that the assumptions of parametric tests, including data distribution and variance homogeneity, were considered before performing ANOVA analyses. Appropriate statistical approaches were selected according to the experimental design and data characteristics. We have also specified the criteria for selecting post-hoc tests and revised the corresponding figure legends to indicate the statistical procedures used for each experiment.

Regarding sample size justification, we have clarified that the number of biological replicates for each experiment is indicated in the corresponding figure legends. In addition, the revised Statistical analysis section now states that sample sizes were selected based on the requirements of the experimental design, previous experience with similar experimental models, and ethical considerations for animal studies.

Regarding Western blot quantification, we have further clarified the normalization strategy in the revised Methods section. Specifically, the expression levels of BAX, BCL2, and cleaved caspase-3 were normalized to GAPDH, while other Western blot analyses were normalized to the corresponding loading controls (GAPDH or β-actin) according to the experimental conditions. The cell-based Western blot analyses were normalized to β-actin. These details have now been explicitly described in the revised manuscript.

We sincerely thank the reviewer for this constructive suggestion, which has helped us improve the clarity and rigor of our statistical reporting and experimental methodology.

*Comments 7:
7. Public Database Analysis Limitations
The authors acknowledge that GEPIA provides ESCA (esophageal carcinoma) data rather than ESCC-specific data. However, the Discussion could more critically address the implications of this limitation. ESCC and esophageal adenocarcinoma (EAC) have distinct molecular profiles, and pooling them in the ESCA cohort may obscure or confound DCN expression patterns specific to ESCC.
Recommendation:
More strongly emphasize that the GEPIA analysis provides supportive but not definitive evidence for DCN dysregulation in ESCC
Consider analyzing ESCC-specific datasets from other sources (e.g., GEO: GSE53625, GSE47404) to strengthen this finding
Discuss the potential for DCN expression to differ between ESCC and EAC subtypes
If possible, filter the GEPIA analysis specifically for the ESCC subtype within the ESCA dataset*

Response 7:
We sincerely thank the reviewer for this important comment regarding the interpretation of the GEPIA-based public-database analysis.

We agree that GEPIA provides TCGA/GTEx esophageal carcinoma (ESCA) data rather than an ESCC-specific dataset. Therefore, we have revised the manuscript to more clearly acknowledge this limitation. In the revised Methods section, we have explicitly stated that GEPIA does not provide an independent ESCC-specific query option, and therefore the ESCA cohort was used for exploratory public-database analysis.

Furthermore, we have strengthened the Discussion by clarifying that the GEPIA findings should be interpreted as supportive rather than definitive evidence for DCN dysregulation in ESCC. We have also discussed that ESCC and esophageal adenocarcinoma (EAC) possess distinct molecular characteristics, and that the inclusion of different histological subtypes within the ESCA cohort may introduce biological heterogeneity and potentially influence subtype-specific interpretation.

Regarding the suggestion to analyze ESCC-specific datasets (e.g., GEO cohorts), we appreciate this valuable recommendation. However, because the current study primarily aimed to investigate the molecular mechanism of SLQG in ESCC using TE-1 xenograft and cell-based models, the GEPIA analysis was included as a supportive external reference rather than as an independent clinical validation study. We therefore consider that additional validation using independent ESCC-specific datasets represents an important direction for future research.

We have revised the relevant sections accordingly to ensure that the interpretation of public-database findings is appropriately cautious and aligned with the limitations of available datasets.

*Comments 8:
8. Western Blot Quantification and Data Presentation
The manuscript states that raw Western blot images are provided in S1 Raw images, which is commendable. However, several details are missing:
Were all samples run on the same gel for each antibody to minimize inter-gel variability?
Was total protein normalization (e.g., stain-free technology) used in addition to housekeeping gene normalization?
Were bands quantified using a consistent exposure time across all samples?
Were loading controls run on the same membranes as target proteins?
Recommendation: Clarify the Western blot quantification protocol in the Methods section, including the normalization strategy and quality control measures.*

Response 8:
We sincerely thank the reviewer for this valuable comment regarding the transparency and rigor of Western blot quantification and data presentation.

We have revised the Methods section to provide additional details regarding our Western blot quality control and normalization procedures. Specifically, we clarified that samples from all experimental groups for each target protein were analyzed on the same gel and membrane to minimize inter-gel variability. We also specified that loading controls (GAPDH or β-actin) were detected on the same membranes as the corresponding target proteins, and target protein expression levels were normalized to the corresponding loading controls.

In addition, we clarified that chemiluminescent images were acquired under non-saturated conditions using consistent imaging criteria for comparative analysis. We further stated that total protein normalization approaches, such as stain-free technology, were not applied in the present study, and therefore our quantification was based on housekeeping protein normalization.

The original uncropped and minimally adjusted Western blot images have been provided in S1 Raw images, as indicated in the revised manuscript.

These revisions improve the methodological transparency of Western blot analysis and clarify the normalization strategy used in this study.

*Comments 9:
9. In Vitro Serum Normalization
In Methods sections 2.4.3–2.4.5, the grouping includes combinations such as "5% SLQG-containing serum + 10% blank serum" for the low-dose group and "15% SLQG-containing serum" for the high-dose group.
It is critical to confirm whether total serum concentration was held constant across all wells to ensure that observed effects are due to SLQG components rather than differences in total serum concentration, which can affect cell proliferation and viability.
Recommendation: Explicitly state in the Methods section whether total serum concentration was normalized across experimental groups (e.g., supplementing lower medicated serum percentages with blank control serum to maintain a constant total serum percentage).*

Response 9:
Dear Reviewer, thank you for your comments. We sincerely thank the reviewer for raising this important methodological concern regarding serum normalization in the in vitro experiments.

We agree that differences in total serum concentration may introduce confounding effects independent of SLQG-derived components. Therefore, we have clarified this issue in the revised Methods section.

Specifically, we have now stated that the total serum concentration was maintained consistently at 15% in all subsequent functional and mechanistic experiments. The low-dose SLQG-containing serum group was prepared using 5% SLQG-containing serum supplemented with 10% blank serum, whereas the control group received 15% blank serum and the high-dose group received 15% SLQG-containing serum. The SB431542 group was treated with 15% blank serum containing SB431542. This normalization strategy ensured that differences among groups were attributable primarily to SLQG-containing serum components rather than variations in total serum concentration.

The corresponding clarification has been added to Sections 2.4.3–2.4.7 of the revised manuscript.

*Comments 10:*

*Minor Revisions and Language Polish
1. Terminology and Nomenclature Errors
Several botanical names are misspelled or used inconsistently.
Corrections:
Line 120: "Gleiniae Radix" → should be "Glehniae Radix" (as correctly spelled in Line 134)
Line 131: "Rabbosiae Rubescentis Herba" → should be "Rabdosiae Rubescentis Herba" (as correctly spelled in Line 136)
Line 123 vs. 146: "Eupatori Herba" vs. "Eupatorii Herba" → choose one consistent genitive form
Gene vs. Protein Nomenclature:
Ensure consistent formatting: human/mouse gene symbols should be italicized (e.g., DCN, TGFB1, CCND1, CDKN1A), whereas protein symbols should remain in standard Roman font (e.g., DCN, TGF-β1, Cyclin D1, p21).*

Response 10:

We sincerely thank the reviewer for carefully checking the nomenclature and terminology throughout the manuscript.

We have revised the botanical names of the herbal components according to the accepted scientific nomenclature. Specifically, “Gleiniae Radix” was corrected to “Glehniae Radix”, “Rabbosiae Rubescentis Herba” was corrected to “Rabdosiae Rubescentis Herba”, and “Eupatori Herba” was standardized as “Eupatorii Herba” throughout the manuscript.

In addition, we carefully reviewed the gene and protein nomenclature according to standard biological conventions. Gene symbols referring to mRNA expression analyses (e.g., DCN, CCND1, CDKN1A, BAX, and BCL2) have been formatted in italics, whereas protein names (e.g., DCN, Cyclin D1, p21, BAX, BCL2, Smad2/3, and GAPDH) have been retained in regular font.

These corrections have been implemented throughout the revised manuscript.

*Comments 11:
2. Typographical and Unit Standardizations
Spacing: Standardize spacing between values and units throughout (e.g., "10 mg/kg" instead of "10mg/kg"; "37 °C" instead of "37℃"; "24 h" instead of "24h"; "100 μL" instead of "100μL").
Line 558: "71.50%" → consider consistency in decimal places throughout Table 1 (some values have two decimals, others two).
Line 802: "α-limonene" vs. "D-limonene" — clarify whether these are distinct compounds or stereoisomers.*

Response 11:
Thank you for pointing out these formatting issues. We have carefully checked the manuscript and standardized the spacing between numerical values and units throughout the text according to journal style, including expressions such as “mg/kg”, “°C”, “h”, and “μL”.

For Table 1, the tumor inhibition rates have been reviewed and formatted consistently with two decimal places.

Regarding the terminology of α-limonene and D-limonene, we have clarified this issue in the revised manuscript. These two terms were retained according to the original annotations from the TCMSP/BATMAN-TCM databases and may represent database-specific nomenclature entries or stereochemical annotations of limonene-related compounds. We have added this clarification in the Results section to avoid potential confusion.

*Comments 12:
3. Figure Legends
Some figure legends lack sufficient detail to be self-explanatory. For example, Figure 5 includes multiple panels with different experimental groups (Model, SLQG-L, SLQG-M, SLQG-H, SB431542), but the sample sizes and specific statistical comparisons are not always clearly indicated in the legend.
Recommendation: Ensure all figure legends include:
Sample sizes (n) for each panel
Clear identification of groups and conditions
Explanation of statistical tests and significance indicators (e.g., P < 0.05, *P < 0.01, **P < 0.001)
For immunohistochemistry, specify the magnification and scale bars*

Response 12:
Thank you for your helpful suggestion regarding the completeness of the figure legends. We have carefully reviewed and revised all figure legends to ensure that each figure is sufficiently self-explanatory.

Specifically, we have added or clarified the sample sizes (n) for each experimental panel, provided clearer descriptions of experimental groups and treatment conditions, and ensured that the statistical methods and significance annotations are consistently described. In addition, the magnification and scale bar information for histological and immunohistochemical images has been explicitly included in the corresponding figure legends.

These revisions have improved the clarity and completeness of the figure legends and allow readers to better interpret the presented data without relying solely on the main text.

*Comments 13:
4. Miscellaneous Corrections
Line 89-91: Specify the method used for mycoplasma testing (e.g., PCR, culture) and provide the testing date or frequency.
Line 151: "Before this name adjustment" → consider revising to "Prior to this name change" for better flow.
Line 194-196: Clarify whether the xenograft experiment and RNA sequencing were performed concurrently or in separate cohorts.
Line 556-558: The SB431542 group shows only 6.93% tumor inhibition—this warrants more explicit discussion (see Major Concern #1).
Line 627-630: The statement "total Smad2/3 expression showed no significant difference among groups" is important for demonstrating specificity; highlight this finding more prominently.
Line 823-824: The non-linear dose-response warrants a more detailed discussion of potential mechanisms (see Major Concern #2).*

Response 13:
Thank you for these careful comments regarding several methodological details and the interpretation of our findings. We appreciate the reviewer’s suggestions, which helped us further improve the clarity and rigor of the manuscript.

Regarding the SB431542 group, we have further clarified the interpretation of this finding in the Discussion section. We acknowledge that SB431542 produced only a limited tumor growth inhibition effect in vivo (6.93%), despite reducing p-Smad2/3 and Cyclin D1 expression. We now emphasize that pharmacological inhibition of TGF-β receptor signaling alone may not fully reproduce the broader biological effects of SLQG, because SLQG may regulate DCN-associated signaling networks involving multiple downstream pathways. Therefore, SB431542 was interpreted as a pathway reference control rather than a direct equivalent of the overall pharmacological action of SLQG.

For the unchanged total Smad2/3 expression, we have highlighted this finding more clearly in the revised manuscript. We now clarify that SLQG primarily affected Smad2/3 phosphorylation rather than total Smad2/3 abundance, suggesting modulation of TGF-β/Smad pathway activation rather than regulation of Smad2/3 expression levels.

Regarding the non-linear dose-response relationship, we have expanded the Discussion section to provide a more cautious interpretation. Specifically, we discussed that the stronger antitumor response observed in the medium-dose group compared with the high-dose group may reflect the complex pharmacological characteristics of multi-component herbal formulations, including differences in absorption, bioavailability, exposure-response relationships, target engagement, and interactions among multiple constituents. We also clarified that the present data do not allow us to conclude that the reduced efficacy of the high-dose group was directly caused by systemic toxicity, and further pharmacokinetic and toxicological studies are warranted.

We thank the reviewer again for these valuable suggestions, which have improved the mechanistic interpretation and transparency of our study.

**Reviewer #3**

*Comments 1:
1. The study design relies heavily on a single ESCC cell line (TE-1). The authors should consider validating the main findings in at least one additional ESCC cell line to improve the generalizability and robustness of the conclusions.*

Response 1:

Thank you for this important comment regarding the use of a single ESCC cell line. We agree that validation across additional ESCC models would further strengthen the generalizability of our findings.

In the revised manuscript, we have further clarified this limitation in the Discussion section. We now acknowledge that although TE-1 is a widely used human ESCC experimental model and allowed consistent evaluation of SLQG-induced molecular and phenotypic alterations in both xenograft and cell-based systems, ESCC is characterized by substantial molecular heterogeneity. Therefore, findings obtained from a single cell line may not fully represent responses across different ESCC subtypes.

We have also refined the interpretation of our conclusions by emphasizing that the current study demonstrates the anti-ESCC effects and DCN-associated regulatory mechanism of SLQG in TE-1-based models, rather than representing a universal mechanism applicable to all ESCC contexts. Future studies using additional ESCC cell lines with distinct molecular backgrounds, as well as orthotopic or patient-derived models, will be important to further validate the translational relevance of these findings.

We appreciate the reviewer’s constructive suggestion, which has helped us better define the scope and limitations of our study.
*Comments 2:
2. Although DCN knockdown experiments are mentioned, the manuscript should clearly demonstrate the efficiency of knockdown and provide stronger quantitative evidence showing how DCN silencing reverses the effects of SLQG on TGF-β/Smad signaling, apoptosis, and cell-cycle regulation.*

Response 2:

We thank the reviewer for this valuable comment regarding the validation of DCN involvement in SLQG-mediated effects.

In the revised manuscript, we have clarified and strengthened the description of the DCN knockdown experiments. Specifically, we have emphasized that siRNA-mediated DCN silencing was validated at both mRNA and protein levels (Fig. 7I–K), confirming the efficiency of DCN knockdown.

Furthermore, we have expanded the Results section to provide a clearer interpretation of the functional consequences of DCN depletion. The revised manuscript now highlights that DCN knockdown partially reversed the effects of SLQG-containing serum on TGF-β/Smad signaling (p-Smad2/3), apoptosis-associated signaling (BAX), and cell-cycle regulation (Cyclin D1) (Fig. 7L–O). These findings provide additional functional evidence that DCN contributes to the regulatory effects of SLQG on ESCC-related biological processes.

We have also revised the Discussion section to clarify that DCN knockdown represents a functional validation approach supporting the role of DCN as an important mediator of SLQG-induced molecular regulation rather than merely an associated molecular change.

We appreciate the reviewer’s suggestion, which has improved the mechanistic interpretation and strengthened the causal relationship between DCN regulation and the biological effects of SLQG.

*Comments 3:
3.The authors should provide information on biological replicates, sequencing depth, quality-control results, normalization procedures, and whether multiple-testing correction was applied for DEG analysis. Using only P < 0.05 without clear adjustment for false discovery may increase the risk of false-positive findings.*

Response 3:

We sincerely appreciate the reviewer’s careful evaluation of our transcriptomic analysis and agree that detailed information regarding biological replicates, sequencing quality control, normalization procedures, and statistical criteria is essential for improving the transparency and reliability of RNA-sequencing studies.

In the revised manuscript, we have expanded the description of the transcriptomic workflow in the Methods section. Specifically, we have clarified the number of biological replicates used for sequencing (*n* = 4 per group), RNA quality assessment procedures, including NanoDrop quantification and Bioanalyzer-based integrity evaluation, library construction procedures, insert size, and sequencing strategy (PE150 sequencing on the Illumina NovaSeq 6000 platform).

We have also revised the description of differential expression analysis to clarify that the initial DEG screening was performed as an exploratory analysis using nominal *P* values. We acknowledge that the absence of multiple-testing correction in the initial screening represents a limitation and that false-positive findings cannot be completely excluded. This limitation has now been explicitly discussed in the revised Discussion section.

Importantly, DCN was not considered a key candidate solely based on transcriptomic screening. Instead, its biological significance was further supported by an integrated validation strategy, including pathway enrichment analysis, public-database analysis, in vivo and in vitro experimental validation, and DCN knockdown experiments. These complementary approaches consistently supported the involvement of DCN in SLQG-mediated regulation of ESCC-related biological processes.

We thank the reviewer for this constructive suggestion, which has helped us improve the methodological transparency and provide a more balanced interpretation of the transcriptomic findings.

*Comments 4:
4.The interpretation of the molecular docking and molecular dynamics results should be more cautious. These computational analyses can support a possible interaction between SLQG-derived compounds and DCN, but they do not prove direct binding or biological activity. The authors should avoid overstating these findings and, if possible, provide experimental validation of the proposed compound–DCN interaction.*

Response 4:

We appreciate the reviewer’s thoughtful comment regarding the interpretation of the molecular docking and molecular dynamics analyses.

We agree that computational approaches can provide valuable mechanistic hypotheses but cannot independently prove direct compound–protein binding or biological activity. In the revised manuscript, we have carefully moderated the description of these analyses and clarified that molecular docking and molecular dynamics simulations were used to explore potential interaction patterns between SLQG-derived compounds and DCN rather than to establish definitive target engagement.

Specifically, we have revised the wording in the Results section to avoid overinterpretation of the docking findings and emphasized that the predicted compound–DCN interactions should be considered as in silico predictions. In addition, we have further clarified in the Discussion section that these computational results provide hypothesis-generating evidence and require further experimental validation using appropriate biophysical approaches, such as surface plasmon resonance, microscale thermophoresis, or cellular thermal shift assays.

We appreciate the reviewer’s suggestion, which has helped us improve the rigor and appropriate interpretation of the computational analyses.

*Comments 5:
5.The clinical relevance and limitations of the study should be discussed more clearly. The findings are based on xenograft and cell-based models, and the public database analysis used the broader ESCA cohort rather than an ESCC-specific dataset. Therefore, the conclusions should be carefully limited to preclinical evidence, and the need for validation in additional ESCC models and clinical samples should be emphasized.*

Response 5:
We sincerely thank the reviewer for this valuable comment. We agree that the clinical implications of our findings should be interpreted cautiously, as the current study primarily provides preclinical evidence derived from TE-1 xenograft and cell-based models.

In the revised manuscript, we have further clarified the limitations associated with the public-database analysis and the experimental models. Specifically, we have emphasized that the GEPIA analysis was performed using the TCGA/GTEx ESCA cohort rather than an ESCC-specific dataset, and therefore these results should be interpreted as supportive evidence of DCN dysregulation rather than definitive evidence specifically representing ESCC.

Furthermore, we have added additional discussion regarding the translational limitations of the current study. Although the combination of xenograft experiments, SLQG-containing serum-treated TE-1 cells, and DCN knockdown experiments provided mechanistic evidence supporting the involvement of DCN-associated regulation, further validation in additional ESCC models, including independent ESCC cell lines, patient-derived models, and clinical tumor specimens, will be necessary to determine the clinical relevance of SLQG and DCN-associated signaling regulation.

These revisions have been incorporated into the Discussion section and the conclusions have been carefully limited to preclinical evidence.

**Reviewer #4**

*Comments:
This study investigates the antitumor effects and mechanisms of Shenling Qige Granules (SLQG) in esophageal squamous cell carcinoma (ESCC) using TE-1 xenograft mice and cell-based models. The authors integrate transcriptomic sequencing, network pharmacology, molecular docking, molecular dynamics simulations, and public database analysis. The study demonstrates that SLQG inhibits tumor growth, upregulates decorin (DCN) expression, inhibits Smad2/3 phosphorylation, promotes apoptosis, and induces G0/G1 cell cycle arrest. siRNA-mediated DCN knockdown attenuates these effects, supporting DCN-TGF-β/Smad involvement.
Overall assessment:
This is a technically sound and well-conducted study with comprehensive methodology and appropriate controls. The data generally support the conclusions. The manuscript is clearly written and presents interesting findings on the mechanism of SLQG in ESCC. However, several areas require attention before publication.
Major Strengths:
• Comprehensive experimental approach integrating multiple complementary methodologies (transcriptomics, network pharmacology, molecular docking, MD simulations, in vivo and in vitro experiments).
• Mechanistic validation through siRNA-mediated DCN knockdown provides functional evidence for DCN involvement.
• Appropriate positive controls (cisplatin, SB431542) and negative controls are included.
• Transparent data availability with raw sequencing data, Western blot images, and numerical data provided.
• Clear presentation of the interesting non-linear dose-response pattern.*

Response :

We sincerely appreciate the reviewer’s careful evaluation of our manuscript and the positive assessment of our study. We are grateful for the recognition of the comprehensive experimental design, the integration of multiple analytical approaches, and the functional validation of DCN involvement in SLQG-mediated antitumor effects.

We are particularly encouraged that the reviewer acknowledged the value of combining transcriptomic analysis, network pharmacology, molecular docking, molecular dynamics simulations, and experimental validation to investigate the mechanism of a complex traditional Chinese medicine formulation. We also appreciate the reviewer’s comments regarding the importance of appropriate controls, transparent data availability, and the observed non-linear dose-response pattern.

The reviewer’s constructive suggestions regarding the remaining issues have been very helpful in improving the clarity, rigor, and interpretation of our manuscript. We have carefully considered each comment and have revised the manuscript accordingly to provide a more balanced discussion of the findings, clarify methodological details, and appropriately define the limitations of the current study.

We thank the reviewer again for the thoughtful review and valuable suggestions, which have helped us further strengthen the quality of our manuscript.

*Comments 1:
Major Concerns Requiring Revision:
• DCN knockdown experiments need strengthening. While the siRNA data support DCN involvement, the incomplete reversal of SLQG effects after DCN knockdown suggests other pathways may contribute. Please consider discussing this more thoroughly and addressing whether DCN overexpression experiments could confirm DCN sufficiency.*

Response 1:

We appreciate the reviewer’s thoughtful comment regarding the interpretation of DCN knockdown experiments and the possibility that additional pathways may contribute to the antitumor effects of SLQG. We agree that the incomplete reversal observed after DCN silencing is an important point that requires careful interpretation, particularly for a multi-component traditional Chinese medicine formulation.

In the revised manuscript, we have expanded the Discussion to clarify that DCN should be considered an important regulatory mediator rather than the sole determinant of SLQG activity. Specifically, we now emphasize that the partial restoration of p-Smad2/3, BAX, and Cyclin D1 changes after DCN knockdown suggests that SLQG may regulate DCN-associated signaling networks together with additional molecular pathways.

Furthermore, we have incorporated the multi-component and multi-target characteristics of SLQG into the mechanistic interpretation and discussed that other candidate compounds identified through network pharmacology, including quercetin, luteolin, ursolic acid, and tanshinone IIA, may contribute to the overall pharmacological effects of the formulation.

Regarding DCN overexpression, we sincerely appreciate the reviewer’s valuable suggestion. We agree that a gain-of-function approach would provide additional evidence regarding whether DCN upregulation is sufficient to reproduce the antitumor effects observed with SLQG. However, the current study was primarily designed to investigate the contribution of DCN to SLQG-mediated regulation through loss-of-function validation. Therefore, we have acknowledged the lack of DCN overexpression or rescue experiments as a limitation and highlighted this approach as an important direction for future investigation.

We thank the reviewer again for this constructive suggestion, which has helped us refine the mechanistic interpretation and appropriately define the role of DCN within the broader pharmacological network of SLQG.

*Comments 2:
• Public database analysis limitations. The GEPIA analysis used the ESCA cohort (includes both ESCC and adenocarcinoma) rather than ESCC-specific data. Please strengthen the discussion of this limitation and consider analyzing available ESCC-specific datasets if possible.*

Response 2:

We appreciate the reviewer’s valuable comment regarding the limitation of the GEPIA-based public database analysis. We agree that the TCGA/GTEx ESCA cohort includes both esophageal squamous cell carcinoma (ESCC) and esophageal adenocarcinoma (EAC), and therefore cannot be regarded as an ESCC-specific dataset.

In the revised manuscript, we have clarified this limitation in both the Methods and Discussion sections. Specifically, we have stated that GEPIA currently provides an ESCA cohort rather than an ESCC-specific analysis option, and therefore the observed DCN expression pattern should be interpreted as supportive evidence of DCN dysregulation in esophageal carcinoma rather than definitive evidence specifically representing ESCC.

Furthermore, we have expanded the Discussion to emphasize that ESCC and EAC possess distinct molecular characteristics and biological behaviors, which may introduce heterogeneity into ESCA-based analyses. We have also highlighted that future validation using independent ESCC-specific datasets, such as GEO-derived cohorts, will be important to further determine the clinical relevance of DCN dysregulation in ESCC.

We thank the reviewer for this important suggestion, which helped us more appropriately define the interpretation and limitation of the public database analysis.

*Comments 3:
• Compound-target validation remains in silico. Please clarify that molecular docking and MD simulations are hypothesis-generating rather than confirmatory. Consider addressing the discrepancy between the 46 UHPLC-HRMS-detected compounds and the 123 network pharmacology candidates.*

Response 3:

We appreciate the reviewer’s important comment regarding the interpretation of compound–target analyses. We agree that molecular docking and molecular dynamics simulations are computational approaches that provide hypothesis-generating evidence but cannot confirm direct compound–target interactions.

Accordingly, we have clarified this point in the Methods and Discussion sections. Specifically, we now emphasize that docking and molecular dynamics simulations were performed to explore potential compound–DCN associations rather than to demonstrate validated target engagement. We have also acknowledged that further experimental approaches, such as surface plasmon resonance, cellular thermal shift assay, or pull-down assays, will be required to confirm direct interactions between SLQG-derived compounds and DCN.

In addition, we have clarified the relationship between the 46 compounds identified by UHPLC-Q-Orbitrap HRMS and the 123 candidate compounds obtained through network pharmacology. Specifically, we now state that the 46 compounds represent experimentally detected constituents of SLQG, whereas the 123 compounds represent database-derived candidates used for mechanistic exploration. This clarification helps distinguish chemical characterization from computational prediction.

We thank the reviewer for this constructive suggestion, which has improved the rigor and transparency of our interpretation of the computational analyses.

*Comments 4:
• The non-linear dose-response pattern (medium dose most effective) requires more discussion. Please address possible reasons including saturation, toxicity, or pharmacokinetic effects.*

Response 4:

We appreciate the reviewer’s thoughtful comment regarding the non-linear dose–response relationship observed in our study. We agree that the finding that the medium dose of SLQG showed greater antitumor efficacy than the high dose requires careful interpretation.

In the revised manuscript, we have expanded the Discussion section to address this pharmacological phenomenon. We now clarify that the reduced efficacy observed in the high-dose group does not necessarily indicate SLQG-induced systemic toxicity, because body-weight reduction was also observed in the Model, DDP, and SB431542 groups, suggesting that these changes may be related to tumor burden and treatment-related physiological effects.

Furthermore, we have discussed several possible explanations for this non-linear response pattern, including differences in absorption, bioavailability, exposure–response relationships, target engagement, and potential counteracting effects among multiple constituents within complex herbal formulations. We have also emphasized that further pharmacokinetic and toxicological studies are required to clarify the relationship between SLQG exposure, biological response, and safety profiles.

We thank the reviewer for this valuable suggestion, which helped us improve the interpretation of the dose-response relationship and avoid overinterpretation of the current findings.

*Comments 5:
• The unexpected SB431542 in vivo results (only 6.93% tumor inhibition) should be discussed more thoroughly. This finding seems inconsistent with the proposed mechanism and requires explanation.*

Response 5:

We sincerely appreciate the reviewer’s careful observation regarding the discrepancy between the limited antitumor effect of SB431542 and the stronger efficacy observed with SLQG in the xenograft model. We agree that this finding required a more nuanced interpretation.

In the revised manuscript, we have expanded the Discussion section to address this discrepancy. We now clarify that pharmacological inhibition of TGF-β receptor signaling alone may not fully reproduce the biological effects induced by SLQG. Several factors may contribute to this difference, including the pharmacodynamic properties, tissue exposure, and duration of action of SB431542 in vivo, as well as the complexity of signaling regulation in TE-1 xenograft tumors.

Furthermore, we have emphasized that DCN is not only involved in TGF-β/Smad regulation but also participates in multiple receptor tyrosine kinase-associated pathways, including EGFR, IGF-1R, and MET signaling. Therefore, the antitumor effects of SLQG should be interpreted as the result of coordinated regulation of DCN-associated signaling networks, with TGF-β/Smad inhibition representing an important but not exclusive mechanism.

We thank the reviewer for this insightful suggestion, which helped us refine the mechanistic interpretation and appropriately moderate our conclusions.

*Comments 6:
Minor Concerns:
• Methodology: Justification for network pharmacology screening thresholds (OB ≥ 30%, DL ≥ 0.18; score ≥ 20) is missing.
• Methodology: The 100 ns molecular dynamics simulation time could be justified more clearly.
• Results: The rationale for focusing primarily on SLQG-M for transcriptomic analysis is not clearly stated.
• Results: In Figure 6, the CCK-8 assay shows decreased viability at 20% serum but less effect than at 15% - this pattern should be discussed.
• Discussion: The statement about β-sitosterol, stigmasterol and ursolic acid forming "relatively stable complexes with DCN" should be qualified as computational prediction.
• Figures: Some figure text is small; consider improving readability.*

Response 6:

We appreciate the reviewer’s careful evaluation of the methodological details and presentation of our study. We have revised the manuscript to address these points and improve the transparency of the experimental design.

For the network pharmacology analysis, we have clarified that the OB ≥ 30%, DL ≥ 0.18, and BATMAN-TCM score ≥ 20 thresholds were selected based on commonly used criteria in traditional Chinese medicine-related pharmacological studies to balance screening efficiency and candidate retention.

For molecular dynamics simulations, we have added a brief explanation that the 100-ns simulation duration was selected as an appropriate timescale to evaluate the conformational stability and dynamic behavior of protein–ligand complexes.

Regarding transcriptomic analysis, we have clarified that SLQG-M was emphasized for downstream mechanistic prioritization because it showed the most consistent antitumor response and the clearest transcriptional separation from the Model group.

For the CCK-8 assay, we have added a statement indicating that the reduced inhibitory effect at 20% compared with 15% SLQG-containing serum represents a non-monotonic response pattern, which is consistent with the complex dose-response characteristics discussed in the revised Discussion.

We have also further clarified that the β-sitosterol–DCN, stigmasterol–DCN and ursolic acid–DCN interactions identified by docking and molecular dynamics simulations represent computational predictions rather than experimentally confirmed binding events.

Finally, figure formatting has been carefully reviewed and adjusted to improve readability.

We thank the reviewer for these constructive suggestions, which helped us improve the methodological clarity and presentation quality of the revised manuscript.

*Comments 7:
Specific Questions for Authors:*

*1. Why was the medium dose more effective than the high dose in vivo? Was any toxicity observed at the high dose?*

Response 7:
We sincerely appreciate the reviewer’s thoughtful question regarding the non-linear dose-response relationship observed in our study. This point is particularly important because dose-dependent efficacy is a critical consideration when interpreting the pharmacological effects of complex herbal formulations.

We agree that the stronger antitumor effect observed in the SLQG-M group compared with the SLQG-H group requires careful interpretation. In the revised Discussion, we have expanded our explanation and emphasized that the reduced efficacy at the high dose should not be directly interpreted as evidence of toxicity, because the present study was not specifically designed as a toxicological evaluation. In addition, body-weight reduction was also observed in other tumor-bearing groups, suggesting that changes in general condition may reflect the complex physiological effects associated with tumor burden and treatment intervention rather than SLQG administration alone.

We further discussed that several pharmacological factors, including differences in absorption, bioavailability, exposure-response relationships, target engagement, and potential counteracting interactions among multiple constituents within SLQG, may contribute to the observed non-linear response pattern. We have also acknowledged that further pharmacokinetic and toxicological investigations are required to clarify the relationship between SLQG exposure, efficacy, and safety.

We thank the reviewer for highlighting this important pharmacological consideration, which helped us refine the interpretation of the dose-response findings.

*Comments 8:
2. Did you observe any correlation between DCN expression and tumor response in individual animals?*

Response 8:
We appreciate the reviewer’s valuable suggestion regarding the relationship between DCN expression and individual tumor responses. We agree that correlation analysis at the individual animal level could provide additional information regarding the association between molecular changes and therapeutic outcomes.

However, such analysis was not included in the original experimental design. The current study evaluated DCN regulation based on group-level comparisons using transcriptomic analysis, RT-qPCR, Western blotting, and immunohistochemistry, together with functional validation through DCN knockdown experiments in vitro. These complementary approaches demonstrated that DCN was consistently upregulated following SLQG treatment and that DCN depletion partially attenuated SLQG-mediated molecular effects.

We acknowledge that individual-level correlation analysis between DCN expression and tumor inhibition was not performed and have considered this point as a potential direction for future studies involving larger cohorts and more comprehensive molecular profiling.

We thank the reviewer for this constructive suggestion, which further highlights the importance of integrating molecular biomarkers with therapeutic responses.

*Comments 9:
3. How do you interpret the SB431542 finding of only 6.93% tumor inhibition in vivo?*

Response 9:
We thank the reviewer for raising this important point regarding the interpretation of the SB431542 xenograft result. We agree that the limited tumor inhibition observed in the SB431542 group requires careful consideration when evaluating the contribution of TGF-β/Smad signaling to SLQG-mediated antitumor activity.

In the revised Discussion, we have further clarified that pharmacological inhibition of TGF-β receptor signaling alone did not reproduce the antitumor efficacy observed with SLQG, suggesting that SLQG does not function exclusively through TGF-β/Smad inhibition. Several factors may contribute to this discrepancy, including differences in in vivo pharmacological properties of SB431542, the complexity of signaling networks within TE-1 xenografts, and the possibility that DCN regulates additional pathways, including receptor tyrosine kinase-associated signaling.

Accordingly, we have moderated our conclusion and now describe the DCN–TGF-β/Smad axis as an important component involved in SLQG-mediated regulation rather than the sole determinant of its antitumor activity.

We sincerely appreciate the reviewer’s careful interpretation of this result, which helped us improve the mechanistic balance of our manuscript.

*Comments 10:
4. How do you reconcile the discrepancy between 123 candidate compounds from network pharmacology and 46 compounds detected by UHPLC-Q-Orbitrap HRMS?*

Response 10:
We appreciate the reviewer’s insightful question regarding the relationship between the network pharmacology-derived candidates and the experimentally detected chemical constituents of SLQG.

We agree that clarification of this issue is important for the interpretation of multi-component herbal formulations. In the revised Discussion, we have explicitly clarified that the 123 candidate compounds identified through network pharmacology represent database-derived candidates for mechanistic exploration, whereas the 46 compounds identified by UHPLC-Q-Orbitrap HRMS represent experimentally detected constituents of the formulation.

These two datasets serve different purposes: the UHPLC-Q-Orbitrap HRMS results provide the chemical basis of SLQG, while network pharmacology provides a hypothesis-generating framework for exploring potential compound–target relationships. Therefore, the candidate compounds identified by network pharmacology should not be interpreted as equivalent to confirmed SLQG constituents or as definitive determinants of pharmacological activity.

We have revised the Discussion accordingly and emphasized that the biological effects of SLQG should be understood within the context of its multi-component and multi-target characteristics.

We thank the reviewer for this important comment, which helped us clarify the integration and interpretation of different analytical approaches.

*Comments 11:
5. Have you considered testing additional ESCC cell lines (e.g., KYSE series) to confirm the findings?*

Response 11:
We sincerely appreciate the reviewer’s thoughtful suggestion regarding validation in additional ESCC models. We fully agree that ESCC is a heterogeneous disease, and confirmation across multiple cell lines would further strengthen the generalizability of the findings.

In the current study, TE-1 cells were selected as the experimental model for both xenograft establishment and in vitro mechanistic validation. The use of a consistent model system allowed us to integrate transcriptomic analysis, in vivo validation, cellular functional assays, and DCN knockdown experiments within the same biological context.

However, we acknowledge that reliance on a single ESCC cell line represents an important limitation. Therefore, we have revised the Discussion to explicitly state that the current findings represent preliminary preclinical evidence requiring further validation in additional ESCC cell lines, orthotopic or immunocompetent models, patient-derived models, and clinical specimens.

We thank the reviewer for this constructive suggestion, which highlights an important direction for future translational studies.

**Reviewer #5**

*Comments:
This is an outstanding piece of scholarly literature; however, there are a few concerns that you should address before publishing.*

Response:
We sincerely thank the reviewer for the highly encouraging and positive evaluation of our manuscript. We greatly appreciate the reviewer’s recognition of the scientific significance and overall quality of our study.

We are also grateful for the constructive comments provided by the reviewer. These insightful suggestions helped us carefully reconsider the interpretation of our mechanistic findings, improve the rigor and balance of our discussion, and provide additional methodological details regarding the preparation and application of SLQG-containing serum.

Following the reviewer’s recommendations, we have revised the manuscript accordingly. Specifically, we have moderated several mechanistic statements to avoid overinterpretation, clarified that the DCN-associated TGF-β/Smad axis represents an important regulatory mechanism involved in SLQG-mediated effects rather than an exclusive pathway, and expanded the methodological description of the serum preparation procedure.

We sincerely appreciate the reviewer’s careful evaluation and valuable suggestions, which have substantially improved the clarity and scientific rigor of the revised manuscript.

*Comments 1:
1. You proposed that SLQG exerts its anti-ESCC effect through a DCN-associated TGF-beta/Smad signalling mechanism. While this correlation was established, there was no mechanistic or evidence-based discussion on whether restoration of DCN expression is sufficient to reproduce, or rescue, the effects attributed to SLQG. You should therefore provide a little bit of functional evidence involving DCN rescue/overexpression and/or complementary pathway manipulation. If there is no available evidence from this study, then you will have to tone down the discussion session relating to the 'effect' of SLQG through the DCN-associated Smad signalling pathway.

The experiment depended on drug-containing rat serum but without description of important details. You should describe how the serum was prepared, the timing of serum collection after SLQG administration, the final serum concentration used in cell experiments, and whether equivalent serum was used for the control groups.*

Response 1:
We sincerely appreciate the reviewer’s insightful comments regarding the interpretation of the DCN-associated mechanism and the methodological details of the SLQG-containing serum experiments.

We agree that the present study did not include DCN overexpression or rescue experiments to determine whether restoration of DCN expression alone is sufficient to reproduce the biological effects of SLQG. Therefore, we have carefully revised the manuscript to avoid overstatement of causality. Specifically, we have modified the description of the DCN–TGF-β/Smad signaling axis from a definitive mechanism to a regulatory axis involved in SLQG-mediated effects. We have also clarified in the Discussion that additional experiments involving DCN overexpression/rescue strategies will be required to determine whether DCN restoration is sufficient to independently reproduce the effects of SLQG.

In the present study, functional evidence supporting the involvement of DCN was obtained through loss-of-function experiments. DCN knockdown partially attenuated SLQG-containing serum-induced changes in p-Smad2/3, BAX, and Cyclin D1 expression, indicating that DCN contributes to SLQG-associated regulation of TGF-β/Smad signaling, apoptosis-related signaling, and cell cycle control.

Regarding the SLQG-containing serum experiments, we appreciate the reviewer’s suggestion and have substantially expanded the methodological description. Briefly, male Sprague–Dawley rats were randomly assigned to SLQG-containing serum and blank serum groups. SLQG was administered by oral gavage for 7 consecutive days, and blood samples were collected 2 h after the final administration. Serum samples were subsequently prepared by clotting, centrifugation, heat inactivation, sterile filtration, and storage at −80 °C. The blank serum group received an equivalent volume of saline and was prepared using the same procedure as the control serum. In the subsequent cellular experiments, equivalent serum concentrations were maintained among groups; the final serum concentration was standardized to 15% by supplementing SLQG-containing serum with blank serum when necessary.

These revisions have been incorporated into the Materials and Methods and Discussion sections of the revised manuscript.

We sincerely thank the reviewer for this valuable suggestion, which helped us improve both the mechanistic interpretation and methodological transparency of our study.

*Comments 2:
2. There is a potentially confounding finding in your research regarding the non-linear response of SLQG. The median dose was shown to have the highest level of efficacy on the experimental models. However, there was no description of the biological basis as to why this efficacy was lost at the high-dose. Could it be differences in exposure, toxicity, formulation-related effects, pharmacokinetics, or variability between experimental groups? Clarify this, and if there are no data on this, kindly indicate it as a limitation. Also, explicitly acknowledge that the optimal medium dose was empirically observed under the experimental conditions rather than established pharmacologically.*

Response 2:
We appreciate the reviewer’s thoughtful comment regarding the non-linear dose–response relationship observed in our study. We agree that the greater antitumor effect observed in the SLQG-M group compared with the SLQG-H group requires cautious interpretation, particularly for a complex multi-component herbal formulation.

In the revised Discussion, we have further clarified that the superior efficacy of the medium dose represents an empirical observation under the current experimental conditions rather than an established optimal pharmacological dose. We have also discussed several possible explanations, including differences in systemic exposure, absorption, bioavailability, target engagement, pharmacokinetic characteristics, and potential interactions among multiple constituents within SLQG.

At present, our study was designed primarily to evaluate the antitumor efficacy and preliminary mechanism of SLQG rather than to establish a dose–exposure–response relationship or perform a dedicated toxicological assessment. Therefore, we cannot determine whether the reduced efficacy observed at the high dose is related to toxicity or other pharmacological factors. We have accordingly acknowledged this issue as a limitation and emphasized that further pharmacokinetic, exposure–response and safety studies are required.

We sincerely appreciate the reviewer for highlighting this important pharmacological consideration, which helped us refine the interpretation of the dose-response findings.

*Comments 3:
3. There are technical issues with your statistical reporting. Firstly, you need to clarify whether the n = 3 reported for Western blot and RT-qPCR experiments represents three independently treated biological samples or repeated measurements from the same sample pool from the nude mice. This should also be clarified across other cell-based experiments. Also, you continuously reported results as standard deviation with p-values and ANOVA for group comparison; was this the only statistical test used for all experimental groups, including cell-based assays? Kindly provide a complete description of the statistical tests used for each experiment. This will aid in increasing the transparency and replicability of these experiments.*

Response 3:
We thank the reviewer for this important comment regarding statistical transparency and experimental reproducibility. We agree that clarification of biological replicates and statistical procedures is essential for accurate interpretation of experimental findings.

In the revised manuscript, we have further clarified the statistical analysis procedures in the Methods section. Specifically, data are presented as mean ± SD, and the assumptions of parametric tests, including data distribution and variance homogeneity, were considered before performing ANOVA analyses. Multiple-group comparisons were analyzed using one-way ANOVA followed by appropriate post hoc tests (Tukey’s or Dunnett’s multiple comparisons test depending on the experimental design), whereas pairwise comparisons were performed using two-tailed Student’s t-test.

We have also ensured that the number of biological replicates is explicitly indicated in the corresponding figure legends. For Western blot, RT-qPCR and other molecular assays, the reported n values represent independent biological samples rather than repeated measurements from the same sample pool. For example, Western blot and RT-qPCR analyses from tumor tissues and cultured cells were performed using independent biological replicates (*n* = 3), as indicated in the figure legends.

We appreciate the reviewer’s suggestion, which helped us improve the methodological transparency and reproducibility of the revised manuscript.

*Comments 4:
4. Technical terms should be first spelt out in full before being abbreviated including DDP as Cisplatin. Especially in the Abstract and Introduction sections.*

Response 4:
We appreciate the reviewer’s careful suggestion regarding the appropriate introduction of technical abbreviations. We have thoroughly checked the manuscript and ensured that all technical terms are first presented in their full forms before abbreviations are used.

Specifically, abbreviations including esophageal squamous cell carcinoma (ESCC), traditional Chinese medicine (TCM), transforming growth factor-β (TGF-β), ultra-high-performance liquid chromatography-quadrupole-Orbitrap high-resolution mass spectrometry (UHPLC-Q-Orbitrap HRMS), Gene Expression Profiling Interactive Analysis (GEPIA), and decorin (DCN) were checked throughout the Abstract and Introduction sections.

In addition, we further clarified the first appearance of cisplatin (DDP) in the Materials and Methods section to ensure consistent terminology throughout the manuscript.

We thank the reviewer for this valuable suggestion, which improved the clarity and readability of the revised manuscript.

*Comments 5:
5. There are occasional paragraphs that are without citations in the discussion section, and are filled with statements of facts not attributed to you or your previous work.*

Response 5:
We appreciate the reviewer’s important comment regarding citation completeness in the Discussion section. We have carefully re-examined the entire Discussion and distinguished statements derived from our experimental findings from those describing established biological knowledge.

Statements describing our own experimental observations, including the effects of SLQG on tumor growth, apoptosis, cell-cycle progression, DCN expression, and TGF-β/Smad signaling modulation, were retained without additional citations because they represent original findings from the present study.

For statements regarding previously established biological functions of DCN and its interactions with receptor tyrosine kinase pathways, we have added additional references where appropriate to ensure that external knowledge is properly attributed. Specifically, the discussion regarding DCN-mediated regulation of EGFR, IGF-1R and MET-related signaling pathways has been supplemented with the corresponding citation.

We thank the reviewer for highlighting this issue, which helped improve the scientific rigor and transparency of the Discussion section.
