# Supplementary material for: Shenling Qige Granules inhibit esophageal squamous cell carcinoma progression in TE-1 xenograft and cell-based models by modulating the DCN–TGF-β/Smad signaling axis
Source: PLoS One. 2026 Oct 6;21(10):e0359405. doi: 10.1371/journal.pone.0359405 (PMC13641691; doi:10.1371/journal.pone.0359405)
Supplement: Peer review history

# Peer review: Decision Letter 0

**DOI:** 10.1371/journal.pone.0359405.r001

**Authors:** Tohru Yamada

20 Aug 2026

Dear Dr. Fan,

Thank you for submitting your manuscript to PLOS ONE. After careful consideration, we feel that it has merit but does not fully meet PLOS ONE’s publication criteria as it currently stands. Therefore, we invite you to submit a revised version of the manuscript that addresses the points raised during the review process.

Please submit your revised manuscript by Oct 19 2026 11:59PM. If you will need more time than this to complete your revisions, please reply to this message or contact the journal office at plosone@plos.org. When you're ready to submit your revision, log on to https://www.editorialmanager.com/pone/ and select the 'Submissions Needing Revision' folder to locate your manuscript file.

- A letter that responds to each point raised by the academic editor and reviewer(s). You should upload this letter as a separate file labeled 'Response to Reviewers'.
- A marked-up copy of your manuscript that highlights changes made to the original version. You should upload this as a separate file labeled 'Revised Manuscript with Track Changes'.
- An unmarked version of your revised paper without tracked changes. You should upload this as a separate file labeled 'Manuscript'.

If you would like to make changes to your financial disclosure, please include your updated statement in your cover letter. Guidelines for resubmitting your figure files are available below the reviewer comments at the end of this letter.

If applicable, we recommend that you deposit your laboratory protocols in protocols.io to enhance the reproducibility of your results. Protocols.io assigns your protocol its own identifier (DOI) so that it can be cited independently in the future. For instructions see: https://journals.plos.org/plosone/s/submission-guidelines#loc-laboratory-protocols. Additionally, PLOS ONE offers an option for publishing peer-reviewed Lab Protocol articles, which describe protocols hosted on protocols.io. Read more information on sharing protocols at https://plos.org/protocols?utm_medium=editorial-email&utm_source=authorletters&utm_campaign=protocols.

As the corresponding author, your ORCID iD is verified in the submission system and will appear in the published article. PLOS supports the use of ORCID, and we encourage all coauthors to register for an ORCID iD and use it as well. Please encourage your coauthors to verify their ORCID iD within the submission system before final acceptance, as unverified ORCID iDs will not appear in the published article. *Only*  the individual author can complete the verification step; PLOS staff *cannot*  verify ORCID iDs on behalf of authors.

We look forward to receiving your revised manuscript.

Kind regards,

Tohru Yamada

Academic Editor

PLOS One

Journal Requirements:

When submitting your revision, we need you to address these additional requirements.

1.Please ensure that your manuscript meets PLOS ONE's style requirements, including those for file naming. The PLOS ONE style templates can be found at

https://journals.plos.org/plosone/s/file?id=wjVg/PLOSOne_formatting_sample_main_body.pdf and

https://journals.plos.org/plosone/s/file?id=ba62/PLOSOne_formatting_sample_title_authors_affiliations.pdf

2. Please note that PLOS One has specific guidelines on code sharing for submissions in which author-generated code underpins the findings in the manuscript. In these cases, we expect all author-generated code to be made available without restrictions upon publication of the work. Please review our guidelines at https://journals.plos.org/plosone/s/materials-and-software-sharing#loc-sharing-code and ensure that your code is shared in a way that follows best practice and facilitates reproducibility and reuse.

3. We note that the grant information you provided in the ‘Funding Information’ and ‘Financial Disclosure’ sections do not match.

When you resubmit, please ensure that you provide the correct grant numbers for the awards you received for your study in the ‘Funding Information’ section.

4. Please include your full ethics statement in the ‘Methods’ section of your manuscript file. In your statement, please include the full name of the IRB or ethics committee who approved or waived your study, as well as whether or not you obtained informed written or verbal consent. If consent was waived for your study, please include this information in your statement as well.

5. Please upload a new copy of Figure 1 to 7 as the detail is not clear. Please follow the link for more information:  https://journals.plos.org/plosone/s/figures.

6. PLOS ONE now requires that authors provide the original uncropped and unadjusted images underlying all blot or gel results reported in a submission’s figures or Supporting Information files. This policy and the journal’s other requirements for blot/gel reporting and figure preparation are described in detail at https://journals.plos.org/plosone/s/figures#loc-blot-and-gel-reporting-requirements and https://journals.plos.org/plosone/s/figures#loc-preparing-figures-from-image-files. When you submit your revised manuscript, please ensure that your figures adhere fully to these guidelines and provide the original underlying images for all blot or gel data reported in your submission. See the following link for instructions on providing the original image data: https://journals.plos.org/plosone/s/figures#loc-original-images-for-blots-and-gels.

In your cover letter, please note whether your blot/gel image data are in Supporting Information or posted at a public data repository, provide the repository URL if relevant, and provide specific details as to which raw blot/gel images, if any, are not available. Email us at plosone@plos.org if you have any questions.

7. Please include captions for your Supporting Information files at the end of your manuscript, and update any in-text citations to match accordingly. Please see our Supporting Information guidelines for more information: http://journals.plos.org/plosone/s/supporting-information.

8. If the reviewer comments include a recommendation to cite specific previously published works, please review and evaluate these publications to determine whether they are relevant and should be cited. There is no requirement to cite these works unless the editor has indicated otherwise.

[Note: HTML markup is below. Please do not edit.]

Reviewer's Responses to Questions

**Comments to the Author**

1. Is the manuscript technically sound, and do the data support the conclusions?

Reviewer #1: Yes

Reviewer #2: Partly

Reviewer #3: No

Reviewer #4: Yes

Reviewer #5: Partly

**********

2. Has the statistical analysis been performed appropriately and rigorously?

Reviewer #1: Yes

Reviewer #2: Yes

Reviewer #3: Yes

Reviewer #4: Yes

Reviewer #5: Yes

**********

3. Have the authors made all data underlying the findings in their manuscript fully available?

The PLOS Data policy

Reviewer #1: Yes

Reviewer #2: Yes

Reviewer #3: Yes

Reviewer #4: Yes

Reviewer #5: Yes

**********

4. Is the manuscript presented in an intelligible fashion and written in standard English?

Reviewer #1: No

Reviewer #2: Yes

Reviewer #3: Yes

Reviewer #4: Yes

Reviewer #5: Yes

**********

Reviewer #1: The manuscript reported Shenling Qige Granules inhibited esophageal squamous cell carcinoma progression. The study appeared incomplete without identifying the active components in the granules. The medicinal properties of Ursolic acid has been well documented in the literature. What are the significances of the In vivo study with nude mice? The responses of carcinoma in appropriate animal model could physiologically demonstrate the cellular activity? The present study involves voluminous set of data using equipment. The results did not satisfactorily support its conclusion. An alternative approach to evaluate the pharmaceutical properties of the granules is warranted. A formulation of phytochemicals could show multi-arrays of pharmaceutical properties. To focus on a few of the compounds is far from satisfactory.

Reviewer #2: Overview & Summary

This manuscript by Liu and colleagues presents a comprehensive preclinical investigation into the antitumor mechanisms of Shenling Qige Granules (SLQG)—a hospital preparation derived from the traditional Chinese medicine formula Qige San—against esophageal squamous cell carcinoma (ESCC). The authors employ an integrative strategy combining transcriptomic RNA sequencing, network pharmacology, molecular docking, 100-ns molecular dynamics (MD) simulations, GEPIA public database analysis, in vivo TE-1 xenografts in nude mice, and in vitro medicated serum assays paired with siRNA-mediated DCN knockdown. The study concludes that SLQG suppresses ESCC proliferation, induces G₀/G₁ cell-cycle arrest, and promotes apoptosis through the DCN–TGF-β/Smad axis.

The study is well-structured, addresses an important therapeutic need in ESCC, and provides commendable transparency regarding raw data and sequencing accessions. However, several major mechanistic, statistical, and conceptual issues must be addressed before the study can meet the rigorous standards required for publication in PLOS ONE.

Major Revisions Required

1. Mechanistic Discrepancy in the In Vivo Xenograft Model

In the in vivo TE-1 xenograft assay, treatment with the selective TGF-β receptor inhibitor SB431542 (10 mg/kg i.p.) achieved a non-significant tumor inhibition rate of only 6.93% relative to the Model group (Table 1; P>0.05). Conversely, SLQG-M achieved a statistically significant inhibition rate of 43.43% (P<0.01).

If pharmacologic blockade of TGF-β signaling via SB431542 fails to significantly attenuate TE-1 tumor growth in vivo, claiming that SLQG inhibits ESCC primarily through the TGF-β/Smad axis is not fully supported by the empirical animal data. This discrepancy raises several questions:

�Is SB431542 adequately bioavailable at the administered dose and route?

�Does the TE-1 xenograft model rely on TGF-β signaling for growth, or are other pathways (EGFR, MET, IGF-1R) more dominant?

�Does SLQG engage parallel pathways that are also regulated by Decorin?

Recommendation: The authors must discuss this discrepancy in depth in the Discussion section. Potential explanations to consider:

�SB431542 may have poor pharmacokinetic properties in vivo (e.g., rapid clearance, protein binding)

�The dose of SB431542 (10 mg/kg) may be insufficient; consider citing literature on effective doses in similar models

�SLQG may act through multiple pathways, with TGF-β/Smad being one component rather than the sole mediator

�DCN's anti-tumor effects may involve receptor tyrosine kinase inhibition (EGFR, IGF-1R, MET) in addition to TGF-β neutralization

The manuscript's assertion regarding the centrality of the TGF-β/Smad axis in vivo should be appropriately nuanced.

2. Non-Monotonic Dose Response and Potential High-Dose Toxicity

The medium dose (SLQG-M) exhibited superior tumor suppression (43.43%) compared to the high dose (SLQG-H, 23.13%).

Figure 3D indicates that mice in the SLQG-H group experienced progressive body weight loss during the treatment period.

In vitro, 15% SLQG-containing serum showed greater efficacy than 20% serum.

This bell-shaped dose-response curve, paired with weight loss at elevated doses, suggests potential systemic toxicity, target saturation, receptor desensitization, or off-target effects at high concentrations. This is a critical pharmacological concern that challenges the interpretation of the results.

Recommendation:

(1) Provide preliminary organ safety/systemic toxicity metrics:

�Serum biochemical parameters (e.g., AST, ALT, BUN, creatinine)

�Representative histopathology of major non-target organs (liver, kidney, heart, lung)

�Organ weight data (liver/kidney/spleen to body weight ratios)

(2) Offer a formal mechanistic interpretation in the Discussion explaining why the high dose yielded reduced antitumor efficacy relative to the medium dose. Potential explanations to consider:

�Saturation of absorption or transport mechanisms

�Receptor desensitization or downregulation at high concentrations

�Presence of both agonistic and antagonistic components in the extract

�High-dose toxicity leading to impaired general health and indirect effects on tumor growth

�Hormetic response or U-shaped dose-response curve

(3) Consider whether repeated-dose pharmacokinetic studies in mice (e.g., measuring plasma concentrations of key compounds at different SLQG doses) would help clarify exposure-response relationships.

(4) Acknowledge this as a limitation that warrants future pharmacokinetic and toxicological investigation.

3. DCN Pathway Specificity and Mechanistic Depth

The siRNA knockdown experiments demonstrate that DCN silencing attenuates SLQG effects (Fig 7L-O), but several important questions remain:

�Are the effects of SLQG mediated primarily through DCN, or is DCN upregulation a secondary consequence of broader pathway modulation? The partial reversal after DCN knockdown (p-Smad2/3 partially restored, BAX and Cyclin D1 changes attenuated) suggests incomplete dependence on DCN.

�Does DCN overexpression alone recapitulate the anti-tumor effects of SLQG? A gain-of-function approach would strengthen the mechanistic argument.

�Why is the effect only partially reversed by DCN knockdown? This may indicate the involvement of additional pathways (identified in network pharmacology) that contribute to SLQG's overall activity.

Recommendation:

�Discuss the "partial reversal" finding more explicitly, acknowledging that SLQG likely acts through multiple targets

�Consider performing DCN overexpression experiments as a complementary gain-of-function approach for future studies

�Address whether other components of the TGF-β/Smad pathway (e.g., Smad4, Smad7, TGFBR1, TGFBR2) were examined in the transcriptomic data

�Discuss the network pharmacology findings in the context of multi-target mechanisms, including other candidate compounds identified (quercetin, luteolin, tanshinone IIA)

4. Rationale for Molecular Docking Against Decorin (DCN)

Small molecules identified in SLQG were docked against the extracellular proteoglycan Decorin (DCN) (Section 2.3.5).

Experimental data confirm that SLQG upregulates DCN mRNA and protein expression levels. Small-molecule natural products typically upregulate extracellular proteins by targeting upstream nuclear receptors, transcription factors, or epigenetic modifiers, rather than binding directly to the secreted mature protein. The biological plausibility of small molecules directly binding to extracellular matrix proteins such as DCN is limited.

Recommendation:

Clarify the biological hypothesis underlying small molecule–DCN binding. Are candidate compounds hypothesized to act as chemical chaperones, stabilize DCN conformation, or alter DCN's binding affinity for TGF-β?

Without direct biophysical binding validation (e.g., surface plasmon resonance [SPR], microscale thermophoresis [MST], or isothermal titration calorimetry [ITC]), clearly state in the text that DCN docking represents exploratory computational modeling rather than validated target engagement.

Consider whether docking to upstream regulators (e.g., transcription factors, epigenetic modifiers) would be more biologically plausible.

5. Single Cell Line Reliance

All cellular mechanisms and xenograft models relied exclusively on the TE-1 cell line.

ESCC exhibits significant histological and molecular heterogeneity. Relying on a single cell line limits the generalizability of the findings and cannot fully recapitulate the histological heterogeneity or organ-specific microenvironment of clinical ESCC.

Recommendation:

Validate key molecular endpoints (e.g., DCN upregulation and p-Smad2/3 suppression) in a second ESCC cell line (e.g., KYSE150, ECA109, KYSE30) to demonstrate generalizability.

If this is not feasible, explicitly acknowledge the single-cell-line limitation in the Discussion, framing the findings as preliminary preclinical evidence requiring validation across a broader panel of ESCC models.

Consider discussing whether future studies using patient-derived organoids or primary ESCC cells would strengthen translational relevance.

6. Statistical Testing and Interpretation Rigor

Standard ANOVA tests were applied, but critical statistical details are missing.

Recommendation:

(1) Explicitly state whether tests for normality (e.g., Shapiro-Wilk) and homogeneity of variances (e.g., Levene's test) were performed prior to parametric testing. If assumptions were violated, describe the corrective measures applied (e.g., data transformation, non-parametric tests).

(2) Clarify the exact post-hoc test used (e.g., Tukey's HSD vs. Dunnett's test) across all figure legends. The Methods section (Section 2.5) states "Tukey's or Dunnett's post hoc test, as appropriate"—specify which test was used for each experiment.

(3) For cell assays involving multiple serum concentrations across time points (e.g., CCK-8 at 5%, 10%, 15%, 20%), ensure appropriate multi-way or repeated-measures ANOVA with multiple comparison corrections (e.g., Bonferroni or Sidak) is applied to prevent Type I error inflation.

(4) Provide a formal sample size justification: While sample sizes are indicated (n=8 for in vivo, n=3 for many molecular assays), no formal power analysis or sample size calculation is provided. Some mechanistic experiments with n=3 may have limited statistical power to detect moderate effect sizes. Consider citing previous studies or standard practice in the field to justify these numbers.

(5) For Western blot quantification, clarify the normalization strategy and whether all samples were run on the same gels.

7. Public Database Analysis Limitations

The authors acknowledge that GEPIA provides ESCA (esophageal carcinoma) data rather than ESCC-specific data. However, the Discussion could more critically address the implications of this limitation. ESCC and esophageal adenocarcinoma (EAC) have distinct molecular profiles, and pooling them in the ESCA cohort may obscure or confound DCN expression patterns specific to ESCC.

Recommendation:

More strongly emphasize that the GEPIA analysis provides supportive but not definitive evidence for DCN dysregulation in ESCC

Consider analyzing ESCC-specific datasets from other sources (e.g., GEO: GSE53625, GSE47404) to strengthen this finding

Discuss the potential for DCN expression to differ between ESCC and EAC subtypes

If possible, filter the GEPIA analysis specifically for the ESCC subtype within the ESCA dataset

8. Western Blot Quantification and Data Presentation

The manuscript states that raw Western blot images are provided in S1 Raw images, which is commendable. However, several details are missing:

Were all samples run on the same gel for each antibody to minimize inter-gel variability?

Was total protein normalization (e.g., stain-free technology) used in addition to housekeeping gene normalization?

Were bands quantified using a consistent exposure time across all samples?

Were loading controls run on the same membranes as target proteins?

Recommendation: Clarify the Western blot quantification protocol in the Methods section, including the normalization strategy and quality control measures.

9. In Vitro Serum Normalization

In Methods sections 2.4.3–2.4.5, the grouping includes combinations such as "5% SLQG-containing serum + 10% blank serum" for the low-dose group and "15% SLQG-containing serum" for the high-dose group.

It is critical to confirm whether total serum concentration was held constant across all wells to ensure that observed effects are due to SLQG components rather than differences in total serum concentration, which can affect cell proliferation and viability.

Recommendation: Explicitly state in the Methods section whether total serum concentration was normalized across experimental groups (e.g., supplementing lower medicated serum percentages with blank control serum to maintain a constant total serum percentage).

Minor Revisions and Language Polish

1. Terminology and Nomenclature Errors

Several botanical names are misspelled or used inconsistently.

Corrections:

Line 120: "Gleiniae Radix" → should be "Glehniae Radix" (as correctly spelled in Line 134)

Line 131: "Rabbosiae Rubescentis Herba" → should be "Rabdosiae Rubescentis Herba" (as correctly spelled in Line 136)

Line 123 vs. 146: "Eupatori Herba" vs. "Eupatorii Herba" → choose one consistent genitive form

Gene vs. Protein Nomenclature:

Ensure consistent formatting: human/mouse gene symbols should be italicized (e.g., DCN, TGFB1, CCND1, CDKN1A), whereas protein symbols should remain in standard Roman font (e.g., DCN, TGF-β1, Cyclin D1, p21).

2. Typographical and Unit Standardizations

Spacing: Standardize spacing between values and units throughout (e.g., "10 mg/kg" instead of "10mg/kg"; "37 °C" instead of "37℃"; "24 h" instead of "24h"; "100 μL" instead of "100μL").

Line 558: "71.50%" → consider consistency in decimal places throughout Table 1 (some values have two decimals, others two).

Line 802: "α-limonene" vs. "D-limonene" — clarify whether these are distinct compounds or stereoisomers.

3. Figure Legends

Some figure legends lack sufficient detail to be self-explanatory. For example, Figure 5 includes multiple panels with different experimental groups (Model, SLQG-L, SLQG-M, SLQG-H, SB431542), but the sample sizes and specific statistical comparisons are not always clearly indicated in the legend.

Recommendation: Ensure all figure legends include:

Sample sizes (n) for each panel

Clear identification of groups and conditions

Explanation of statistical tests and significance indicators (e.g., P < 0.05, *P < 0.01, **P < 0.001)

For immunohistochemistry, specify the magnification and scale bars

4. Miscellaneous Corrections

Line 89-91: Specify the method used for mycoplasma testing (e.g., PCR, culture) and provide the testing date or frequency.

Line 151: "Before this name adjustment" → consider revising to "Prior to this name change" for better flow.

Line 194-196: Clarify whether the xenograft experiment and RNA sequencing were performed concurrently or in separate cohorts.

Line 556-558: The SB431542 group shows only 6.93% tumor inhibition—this warrants more explicit discussion (see Major Concern #1).

Line 627-630: The statement "total Smad2/3 expression showed no significant difference among groups" is important for demonstrating specificity; highlight this finding more prominently.

Line 823-824: The non-linear dose-response warrants a more detailed discussion of potential mechanisms (see Major Concern #2).

Reviewer #3: 1. The study design relies heavily on a single ESCC cell line (TE-1). The authors should consider validating the main findings in at least one additional ESCC cell line to improve the generalizability and robustness of the conclusions.

2.Although DCN knockdown experiments are mentioned, the manuscript should clearly demonstrate the efficiency of knockdown and provide stronger quantitative evidence showing how DCN silencing reverses the effects of SLQG on TGF-β/Smad signaling, apoptosis, and cell-cycle regulation.

3.The authors should provide information on biological replicates, sequencing depth, quality-control results, normalization procedures, and whether multiple-testing correction was applied for DEG analysis. Using only P < 0.05 without clear adjustment for false discovery may increase the risk of false-positive findings.

4.The interpretation of the molecular docking and molecular dynamics results should be more cautious. These computational analyses can support a possible interaction between SLQG-derived compounds and DCN, but they do not prove direct binding or biological activity. The authors should avoid overstating these findings and, if possible, provide experimental validation of the proposed compound–DCN interaction.

5.The clinical relevance and limitations of the study should be discussed more clearly. The findings are based on xenograft and cell-based models, and the public database analysis used the broader ESCA cohort rather than an ESCC-specific dataset. Therefore, the conclusions should be carefully limited to preclinical evidence, and the need for validation in additional ESCC models and clinical samples should be emphasized.

Reviewer #4: This study investigates the antitumor effects and mechanisms of Shenling Qige Granules (SLQG) in esophageal squamous cell carcinoma (ESCC) using TE-1 xenograft mice and cell-based models. The authors integrate transcriptomic sequencing, network pharmacology, molecular docking, molecular dynamics simulations, and public database analysis. The study demonstrates that SLQG inhibits tumor growth, upregulates decorin (DCN) expression, inhibits Smad2/3 phosphorylation, promotes apoptosis, and induces G0/G1 cell cycle arrest. siRNA-mediated DCN knockdown attenuates these effects, supporting DCN-TGF-β/Smad involvement.

Overall assessment:

This is a technically sound and well-conducted study with comprehensive methodology and appropriate controls. The data generally support the conclusions. The manuscript is clearly written and presents interesting findings on the mechanism of SLQG in ESCC. However, several areas require attention before publication.

Major Strengths:

• Comprehensive experimental approach integrating multiple complementary methodologies (transcriptomics, network pharmacology, molecular docking, MD simulations, in vivo and in vitro experiments).

• Mechanistic validation through siRNA-mediated DCN knockdown provides functional evidence for DCN involvement.

• Appropriate positive controls (cisplatin, SB431542) and negative controls are included.

• Transparent data availability with raw sequencing data, Western blot images, and numerical data provided.

• Clear presentation of the interesting non-linear dose-response pattern.

Major Concerns Requiring Revision:

• DCN knockdown experiments need strengthening. While the siRNA data support DCN involvement, the incomplete reversal of SLQG effects after DCN knockdown suggests other pathways may contribute. Please consider discussing this more thoroughly and addressing whether DCN overexpression experiments could confirm DCN sufficiency.

• Public database analysis limitations. The GEPIA analysis used the ESCA cohort (includes both ESCC and adenocarcinoma) rather than ESCC-specific data. Please strengthen the discussion of this limitation and consider analyzing available ESCC-specific datasets if possible.

• Compound-target validation remains in silico. Please clarify that molecular docking and MD simulations are hypothesis-generating rather than confirmatory. Consider addressing the discrepancy between the 46 UHPLC-HRMS-detected compounds and the 123 network pharmacology candidates.

• The non-linear dose-response pattern (medium dose most effective) requires more discussion. Please address possible reasons including saturation, toxicity, or pharmacokinetic effects.

• The unexpected SB431542 in vivo results (only 6.93% tumor inhibition) should be discussed more thoroughly. This finding seems inconsistent with the proposed mechanism and requires explanation.

Minor Concerns:

• Methodology: Justification for network pharmacology screening thresholds (OB ≥ 30%, DL ≥ 0.18; score ≥ 20) is missing.

• Methodology: The 100 ns molecular dynamics simulation time could be justified more clearly.

• Results: The rationale for focusing primarily on SLQG-M for transcriptomic analysis is not clearly stated.

• Results: In Figure 6, the CCK-8 assay shows decreased viability at 20% serum but less effect than at 15% - this pattern should be discussed.

• Discussion: The statement about β-sitosterol, stigmasterol and ursolic acid forming "relatively stable complexes with DCN" should be qualified as computational prediction.

• Figures: Some figure text is small; consider improving readability.

Specific Questions for Authors:

1. Why was the medium dose more effective than the high dose in vivo? Was any toxicity observed at the high dose?

2. Did you observe any correlation between DCN expression and tumor response in individual animals?

3. How do you interpret the SB431542 finding of only 6.93% tumor inhibition in vivo?

4. How do you reconcile the discrepancy between 123 candidate compounds from network pharmacology and 46 compounds detected by UHPLC-Q-Orbitrap HRMS?

5. Have you considered testing additional ESCC cell lines (e.g., KYSE series) to confirm the findings?

Reviewer #5: This is an outstanding piece of scholarly literature; however, there are a few concerns that you should address before publishing.

1. You proposed that SLQG exerts its anti-ESCC effect through a DCN-associated TGF-beta/Smad signalling mechanism. While this correlation was established, there was no mechanistic or evidence-based discussion on whether restoration of DCN expression is sufficient to reproduce, or rescue, the effects attributed to SLQG. You should therefore provide a little bit of functional evidence involving DCN rescue/overexpression and/or complementary pathway manipulation. If there is no available evidence from this study, then you will have to tone down the discussion session relating to the 'effect' of SLQG through the DCN-associated Smad signalling pathway.

The experiment depended on drug-containing rat serum but without description of important details. You should describe how the serum was prepared, the timing of serum collection after SLQG administration, the final serum concentration used in cell experiments, and whether equivalent serum was used for the control groups.

2. There is a potentially confounding finding in your research regarding the non-linear response of SLQG. The median dose was shown to have the highest level of efficacy on the experimental models. However, there was no description of the biological basis as to why this efficacy was lost at the high-dose. Could it be differences in exposure, toxicity, formulation-related effects, pharmacokinetics, or variability between experimental groups? Clarify this, and if there are no data on this, kindly indicate it as a limitation. Also, explicitly acknowledge that the optimal medium dose was empirically observed under the experimental conditions rather than established pharmacologically.

3. There are technical issues with your statistical reporting. Firstly, you need to clarify whether the n = 3 reported for Western blot and RT-qPCR experiments represents three independently treated biological samples or repeated measurements from the same sample pool from the nude mice. This should also be clarified across other cell-based experiments. Also, you continuously reported results as standard deviation with p-values and ANOVA for group comparison; was this the only statistical test used for all experimental groups, including cell-based assays? Kindly provide a complete description of the statistical tests used for each experiment. This will aid in increasing the transparency and replicability of these experiments.

4. Technical terms should be first spelt out in full before being abbreviated including DDP as Cisplatin. Especially in the Abstract and Introduction sections.

5. There are occasional paragraphs that are without citations in the discussion section, and are filled with statements of facts not attributed to you or your previous work.

**********

what does this mean?). If published, this will include your full peer review and any attached files.

If you choose “no”, your identity will remain anonymous but your review may still be made public.

**Do you want your identity to be public for this peer review?**  For information about this choice, including consent withdrawal, please see our Privacy Policy

Reviewer #1: No

Reviewer #2: No

Reviewer #3: **Yes:**  Sakarie Mustafe Hidig,MD

Reviewer #4: No

Reviewer #5: **Yes:**  Chijioke Noris Ezinwa

**********

[NOTE: If reviewer comments were submitted as an attachment file, they will be attached to this email and accessible via the submission site. Please log into your account, locate the manuscript record, and check for the action link "View Attachments". If this link does not appear, there are no attachment files.]

To ensure your figures meet our technical requirements, please review our figure guidelines: https://journals.plos.org/plosone/s/figures

You may also use PLOS’s free figure tool, NAAS, to help you prepare publication quality figures: https://journals.plos.org/plosone/s/figures#loc-tools-for-figure-preparation.

NAAS will assess whether your figures meet our technical requirements by comparing each figure against our figure specifications.

# Author comment: Author response to Decision Letter 1

**DOI:** 10.1371/journal.pone.0359405.r002

26 Aug 2026

Thank you for the opportunity to revise our manuscript. We have carefully addressed all comments raised by the Editor and the five reviewers and have revised the manuscript accordingly. A detailed point-by-point response to all reviewer comments has been provided in the uploaded “Response to Reviewers” document. All revisions in the marked manuscript are highlighted in yellow for ease of reference. We sincerely appreciate the Editor and reviewers for their constructive comments and suggestions, which have helped us improve the clarity, rigor, and overall quality of the manuscript.

Attachment Submitted filename: Response to Reviewers.docx

# Peer review: Decision Letter 1

**DOI:** 10.1371/journal.pone.0359405.r003

**Authors:** Tohru Yamada

15 Sep 2026

Shenling Qige Granules inhibit esophageal squamous cell carcinoma progression in TE-1 xenograft and cell-based models by modulating the DCN–TGF-β/Smad signaling axis

PONE-D-26-31883R1

Dear Dr. Fan,

We’re pleased to inform you that your manuscript has been judged scientifically suitable for publication and will be formally accepted for publication once it meets all outstanding technical requirements.

Within one week, you’ll receive an e-mail detailing the required amendments. When these have been addressed, you’ll receive a formal acceptance letter and your manuscript will be scheduled for publication.

An invoice will be generated when your article is formally accepted. Please note, if your institution has a publishing partnership with PLOS and your article meets the relevant criteria, all or part of your publication costs will be covered. Please make sure your user information is up-to-date by logging into Editorial Manager at Editorial Manager® and clicking the ‘Update My Information' link at the top of the page. For questions related to billing, please contact billing support.

If your institution or institutions have a press office, please notify them about your upcoming paper to help maximize its impact. If they’ll be preparing press materials, please inform our press team as soon as possible -- no later than 48 hours after receiving the formal acceptance. Your manuscript will remain under strict press embargo until 2 pm Eastern Time on the date of publication. For more information, please contact onepress@plos.org.

Kind regards,

Tohru Yamada

Academic Editor

PLOS One

Additional Editor Comments (optional):

Reviewers' comments:

Reviewer's Responses to Questions

**Comments to the Author**

Reviewer #1: All comments have been addressed

Reviewer #2: All comments have been addressed

Reviewer #3: All comments have been addressed

Reviewer #5: All comments have been addressed

**********

2. Is the manuscript technically sound, and do the data support the conclusions?

Reviewer #1: Yes

Reviewer #2: Yes

Reviewer #3: Yes

Reviewer #5: Yes

**********

3. Has the statistical analysis been performed appropriately and rigorously?

Reviewer #1: Yes

Reviewer #2: Yes

Reviewer #3: Yes

Reviewer #5: Yes

**********

4. Have the authors made all data underlying the findings in their manuscript fully available?

The PLOS Data policy

Reviewer #1: Yes

Reviewer #2: Yes

Reviewer #3: Yes

Reviewer #5: Yes

**********

5. Is the manuscript presented in an intelligible fashion and written in standard English?

Reviewer #1: Yes

Reviewer #2: Yes

Reviewer #3: Yes

Reviewer #5: Yes

**********

Reviewer #1: The manuscript has been revised in accordance with the reviewers' comments, and the revised manuscript reads better.

Reviewer #2: (No Response)

Reviewer #3: The authors have fully addressed all of my questions, and I have no further requests. The revisions provided are thorough and completely resolve the points raised. I appreciate the clarity and detail in their responses. Everything is now complete.

Reviewer #5: (No Response)

**********

what does this mean?). If published, this will include your full peer review and any attached files.

If you choose “no”, your identity will remain anonymous but your review may still be made public.

**Do you want your identity to be public for this peer review?**  For information about this choice, including consent withdrawal, please see our Privacy Policy

Reviewer #1: No

Reviewer #2: No

Reviewer #3: **Yes:**  Sakarie Mustafe Hidig,MD

Reviewer #5: No

**********

# Editor report: Acceptance letter

**DOI:** 10.1371/journal.pone.0359405.r004

**Authors:** Tohru Yamada

PONE-D-26-31883R1

PLOS One

Dear Dr. Fan,

I'm pleased to inform you that your manuscript has been deemed suitable for publication in PLOS One. Congratulations! Your manuscript is now being handed over to our production team.

At this stage, our production department will prepare your paper for publication. This includes ensuring the following:

* All references, tables, and figures are properly cited

* All relevant supporting information is included in the manuscript submission,

* There are no issues that prevent the paper from being properly typeset

You will receive further instructions from the production team, including instructions on how to review your proof when it is ready. Please keep in mind that we are working through a large volume of accepted articles, so please give us a few days to review your paper and let you know the next and final steps.

Lastly, if your institution or institutions have a press office, please let them know about your upcoming paper now to help maximize its impact. If they'll be preparing press materials, please inform our press team within the next 48 hours. Your manuscript will remain under strict press embargo until 2 pm Eastern Time on the date of publication. For more information, please contact onepress@plos.org.

You will receive an invoice from PLOS for your publication fee after your manuscript has reached the completed accept phase. If you receive an email requesting payment before acceptance or for any other service, this may be a phishing scheme. Learn how to identify phishing emails and protect your accounts at https://explore.plos.org/phishing.

If we can help with anything else, please email us at customercare@plos.org.

Thank you for submitting your work to PLOS One and supporting open access.

Kind regards,

PLOS One Editorial Office Staff

on behalf of

Dr. Tohru Yamada

Academic Editor

PLOS One
